# Genomic Insights into the Bactericidal and Fungicidal Potential of *Bacillus mycoides* b12.3 Isolated in the Soil of Olkhon Island in Lake Baikal, Russia

**DOI:** 10.3390/microorganisms12122450

**Published:** 2024-11-28

**Authors:** Maria N. Romanenko, Anton E. Shikov, Iuliia A. Savina, Fedor M. Shmatov, Anton A. Nizhnikov, Kirill S. Antonets

**Affiliations:** 1 All-Russia Research Institute for Agricultural Microbiology, 196608 St. Petersburg, Russia; m.romanenko@arriam.ru (M.N.R.); a.shikov@arriam.ru (A.E.S.);; 2 Faculty of Biology, St. Petersburg State University, 199034 St. Petersburg, Russia

**Keywords:** *Bacillus mycoides*, strain isolation, bactericidal properties, fungicidal activity, plant pathogens, biosynthetic gene clusters, Lake Baikal

## Abstract

The dispersal of plant pathogens is a threat to the global economy and food industry which necessitates the need to discover efficient biocontrol agents such as bacteria, fungi, etc., inhibiting them. Here, we describe the *Bacillus mycoides* strain b12.3 isolated from the soil of Olkhon Island in Lake Baikal, Russia. By applying the co-cultivation technique, we found that the strain inhibits the growth of plant pathogens, such as the bacteria *Xanthomonas campestris*, *Clavibacter michiganensis*, and *Pectobacterium atrospecticum,* as well as the fungus *Alternaria solani*. To elucidate the genomic fundament explaining these activities, we leveraged next-generation whole-genome sequencing and obtained a high-quality assembly based on short reads. The isolate bore seven known BGCs (biosynthetic gene clusters), including those responsible for producing bacillibactin, fengycin, and petrobactin. Moreover, the genome contained insecticidal genes encoding for App4Aa1, Tpp78Ba1, and Spp1Aa1 toxins, thus implicating possible pesticidal potential. We compared the genome with the 50 closest assemblies and found that b12.3 is enriched with BGCs. The genomic analysis also revealed that genomic architecture corresponds to the experimentally observed activity spectrum implying that the combination of produced secondary metabolites delineates the range of inhibited phytopathogens Therefore, this study deepens our knowledge of the biology and ecology of *B. mycoides* residing in the Lake Baikal region.

## 1. Introduction

Lake Baikal, the largest (23,015 km^3^) and deepest lake (1642 m) in the world, holds approximately 20% of the world’s freshwater [1]. It encompasses highly diverse biotopes, e.g., hot springs, oil seeps, planktonic flora and fauna, sediments formed by diatomaceous silts, and water columns of varying depths. The lake is known for its unique conditions, namely, low temperature and mineral content, high oxygen concentration, and oligotrophic nature. Given the features mentioned above, it is considered the richest reservoir of endemic biodiversity, including microorganisms [2,3]. Multiple research efforts have been made to characterize microbial communities in Lake Baikal, including studies of ice particles [4], algae [5], sediments [6], littoral zone [7], hot springs [2], oil seeps [8], and water columns [9].

The microbiome of the lake hides industrial and agricultural potential due to multiple functional activities found in its residential isolates. Endemic representatives from distinct ecotopes were shown to produce a variety of broad-spectrum bactericidal metabolites [5,6,7,10]. Among prospective strains, the genus *Bacillus* occupying sediments and coastal surfaces was demonstrated to carry the highest fraction of NRPS (non-ribosomal peptide synthetases) genes in the whole gene pool [5,6]. The genus belongs to the phylum Firmicutes, which is sporadically detected in water [11], while constituting a substantial part of the sediments’ microbiome [6,12]. One notable specimen of sedimental bacilli was *B. mycoides* strain BS2-15 displaying strong algicidal activity [13]. At the same time, studies reporting the strains isolated in the Baikal region belonging to this species remain scarce [13,14,15].

*B. mycoides* is a Gram-positive spore-forming bacterium typically residing in soil. From a taxonomic perspective, it belongs to the *Bacillus cereus sensu lato* group. After genome-wise reclassification, the bacterium was named Genomospecies VI, combining both *B. weihenstephanensis* and *B. mycoides* [16]. We still lack a detailed understanding of *B. mycoides*’ ecology; however, the available data suggest its ubiquity as well as its prominent role in ecological networks due to genetic plasticity [17]. The members of the species are frequently associated with plants as endophytic, rhizospheric, or epiphytic residents [18,19,20]. Colonization is often beneficial for host plants since *B. mycoides* strains could act as plant growth-promoting bacteria (PGPB) via a broad assortment of mechanisms ranging from biofertilization [19] to protection from plant pathogens [21,22,23]. The antipathogenic properties stem from the ability to synthesize bioactive bactericidal and fungicidal metabolites such as bacteriocins [24], volatile organic compounds (VOCs) [25], lipopeptides [26], and others.

Active compounds are synthesized by enzymes encoded by biosynthetic gene clusters (BGCs). Therefore, genomic screening for such loci could ease the preliminary selection of potential biocontrol agents, biofertilizers, and PGP. The latter could be achieved through direct or indirect mechanisms [27]. The first implies the production of plant phytohormones regulating plant development [28] and siderophores which ameliorate Fe^3+^ uptake from soil [27] and sequester heavy metals controlling the phytoremediation process in contaminated soils [29]. Indirect effects, in turn, primarily lie in inhibiting plant pathogens and increasing host adaptation to multiple environmental stressors [27,29,30,31]. By current reckonings, the known BGCs amount to only circa 20% of the present diversity [32]. Taking into account the multi-faced action of synthesized secondary metabolites and the emergence of plant pathogens resistant to commonly used pesticides [33], which can be circumvented by applying biopreparations instead of synthetic pesticides or with them [34], there is a need to assess the most efficient combinations of BGCs and understand which factors delineate specific antipathogenic activities of prospective agricultural strains.

The ramifications of plant diseases are detrimental to the world’s food production, especially in developing countries [35]. By current reckonings, plant-associated pathogens reduce crop yield by up to 30% [36]. Bacteria and fungi account for 27% and 42% of them, respectively [35]. Designing strategies to address this issue is a daunting challenge due to the emergence of resistant populations. The leaf blight-causing agent, *Alternaria solani*, has become a major problem in agriculture of northwestern Europe due to the dissemination of strains with low sensitivity to commercial fungicides [37]. Similarly, losses of pepper and potato plants provoked by *Xanthomonas campestris* and *Pectobacterium atrosepticum* reached 23–44% [38] and 45% [39], respectively. Given the necessity to alleviate pathogen-induced damage, the application of biocontrol agents is considered a viable solution. In this context, effective methods for screening prospective strains are needed.

Despite the wide array of microbiological and metagenomic studies using samples from distinct niches of Lake Baikal, little is known about the microbiota of soil in the islands within the lake’s water basin. On that account, we selected one spore-forming strain, b12.3, identified as *B*.*mycoides*. This study aims to describe the first *B. mycoides* occupying the soil ecological niche in the Lake Baikal region using experimental identification of its antipathogenic activity and further reveal the associations between BGCs and the spectrum of inhibited phytopathogens by examining its genome, obtained via next-generation sequencing.

## 2. Materials and Methods

### 2.1. Isolation of the Strain from a Soil Sample

The strain was isolated from a soil sample collected on a hillside on Olkhon Island (53.31898 107.74190, Lake Baikal, Russia) in June 2020. A total of 6 strains were isolated from this sample, including strain b12.3, presented in this work. The strain b12.3 was deposited in the joint Russian Collection of Agricultural Microorganisms (RCAM) at the All-Russia Research Institute for Agricultural Microbiology in Saint Petersburg (http://62.152.67.70/cryobank/login.jsp, accessed on 14 August 2024) in December 2022. The registration number is RCAM06147.

**Figure 1:**
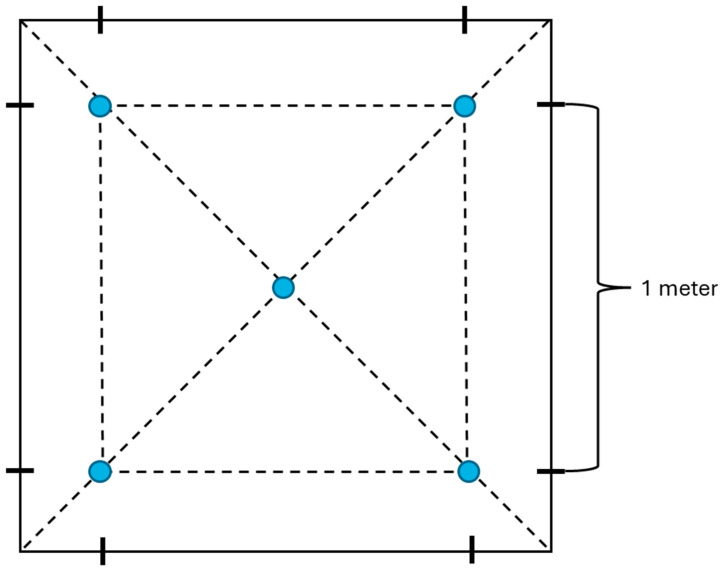
Protocol for sampling using the “envelope” method. Blue circles indicate sampling areas.

The sampling was carried out according to the following scheme. Soil from a depth of 3–5 cm was removed with a sterile mini garden shovel and placed in a clean zip bag. Samples from each location were collected at a distance of about 1 m from one another using the “envelope” method (Figure 1).

To retrieve the strain, we first suspended individual soil portions (0.2 g) in 2 mL of sterile water and homogenized them by vigorous stirring for 10 min. The resulting homogenate was then placed in Eppendorf tubes, 1 mL each, and heated in a water bath (80 °C) for 30 min to destroy non-spore-forming microorganisms and vegetative *Bacillus* cells [40]. After that, serial dilutions of 10, 100, and 1000 times (the first, second, and third dilutions, respectively) were prepared, and 200 μL of each dilution was inoculated onto Petri dishes with selective T3 agar nutrient medium (tryptone 3 g/L; tryptose 2 g/L; yeast extract 1.5 g/L; 270 NaH_2_PO_4_·H_2_O 6.9 g/L; MnCl_2_·4H_2_O 0.008 g/L; agar 15 g/L; pH 6.8) [41] to induce the sporulation process. The obtained isolates were incubated for 72 h at 28 °C. Thereafter, the colonies of interest meeting the expected morphological criteria were picked with subsequent repetitive transfers to fresh T3 agar nutrient medium [41] until a pure culture of the strain was obtained.

### 2.2. Characterization of the Strain by Microscopy and Colony Morphology

The morphology of vegetative cells and spores stained with Coomassie Brilliant Blue was inspected using a light microscope. Bacteria were grown on CCY agar medium (0.5 mM MgCl_2_ 6H_2_O; 0.01 mM MnCl_2_ 4H_2_O; 0.05 mM FeCl_3_ 6H_2_O; 0.05 mM ZnCl_2_; 0.2 mM CaCl_2_ 6H_2_O; 13 mM KH_2_PO_4_; 26 mM K_2_HPO_4_; 20 mg/L glutamine; 1 g/L acid casein hydrolysate; 1 g/L enzymatic casein hydrolysate; 0.4 g/L enzymatic yeast extract; 0.6 g/L glycerol; agar 20 g/L) [42] for 2 days to reach the onset of sporulation. One or two colonies (approximately 10 μL) were placed in a drop of sterile water on a glass slide, suspended, dried, and dyed in a container for 1–15 min accordingly. The preparations were then rinsed with distilled water, dried, and viewed at 1000× magnification.

To scrutinize the morphological features of the colonies, bacteria were grown on Petri dishes with LB (Luria–Bertani, Miller, VWR International Inc., Solon, OH, USA) agar medium for 1 day at 28 °C. The colonies were described morphologically in terms of their surface, profile, optical properties, color, edge shape, colony shape, and consistency.

### 2.3. Identification of Isolates Based on the gyrB Locus Analysis

To extract DNA for the *gyrB* gene sequencing, the strain was incubated on Petri dishes with LB medium at 28 °C for 16–18 h. Then, bacterial cells were suspended in Tris-EDTA (TE) buffer (pH 7.5), heated for 10 min at 99 °C, and centrifuged at 15,000× *g* for 15 min at +4 °C to eliminate cellular debris. The supernatant was transferred to new tubes and stored at −20 °C [43,44].

The isolated DNA was used to set up PCR under the following conditions. Each reaction volume (20 µL) contained 0.8 µL bacterial DNA, 10 µL Fermentas DreamTaq green PCR master mix (Thermo Fisher Scientific, Waltham, MA, USA), and 0.3 µL of each primer (concentration 100 pmol/µL) (Table S1).

The PCR program included an initial denaturation of 3 min at 94 °C, followed by 30 cycles of denaturation for 30 s at 95 °C, annealing for 30 s at 52 °C, and elongation at 72 °C for 1 min 30 s. A final elongation was performed for 7 min at 72 °C, and the program was completed with a storage step at 12 °C. PCR products were verified by electrophoresis in 1% agarose gel stained with 0.002% ethidium bromide by comparison with the λ DNA/HindIII marker (Thermo Fisher Scientific, Inc., Waltham, MA, USA).

The nucleotide sequence of the *gyrB* gene was determined using the Sanger sequencing method [45]. At the first stage of sample preparation for sequencing, they were purified from primer residues and deoxynucleotide phosphates not included in the chain. The reaction mixture included 5 μL of PCR product, 0.5 μL of exonuclease 1 (Exo I), and 1 μL of FastAPTM thermosensitive alkaline phosphatase. The mixture was then thoroughly mixed and incubated for 15 min at 37 °C. The reaction was stopped by heating the mixture to 85 °C for 15 min [46].

Next, 1.5 µL of primer (concentration 100 pmol/µL) and 1.5 µL of sterile water were added to 2 µL of purified PCR product. The sequencing was performed using equipment of the Core Centrum ‘Genomic Technologies, Proteomics and Cell Biology’ in ARRIAM.

### 2.4. Assessing the Bactericidal and Fungicidal Activity of the Strain Against Plant Pathogens of Agricultural Crops

The list of bacteria and fungi used for screening for fungicidal and bactericidal activities is provided in Table 1. The bactericidal activity of the strain was tested using the cross-streak method [47] on Petri dishes with a diameter of 60 mm with 2YT agar nutrient medium (16 g/L tryptone, 10 g/L yeast extract, 5.0 g/L NaCl, 20 g/L agar) [48]. In the first stage, strain b12.3 was placed centrally on Petri dishes along the entire dish and incubated at +28 C for 2 days. Once incubated, single streaks of plant pathogenic bacteria were inoculated perpendicular to the strain. There was one phytopathogen per dish, and each phytopathogenic strain-containing dish was examined in 10 replicates. Microbial interactions were assessed by measuring the size of the inhibition zone.

The fungicidal properties of the strain were assessed using the co-cultivation method previously described by López-Gonzálezet al., 2021 [49]. At 1–2 cm from the edge of a Petri dish (diameter 94 mm) with agarized (2 g per 100 mL) potato dextrose broth (PDB) nutrient medium (Himedia Laboratories Pvt. Ltd., Dindori, Maharashtra, India), the mycelium of plant pathogenic fungi was inoculated. After the cultivation for two days at +28 °C, strain b12.3 was moved to the fungus-containing dishes. More specifically, it was placed 1–2 cm from the edge of the dish on the side opposite the fungi. The plates with both the fungi and bacteria were cultivated at +28 °C. The cultivation lasted until the fungi reached a growth stage suitable for visually identifying the extent of inhibition.

Strain c8.2, deposited in RCAM at the All-Russia Research Institute for Agricultural Microbiology in Saint Petersburg (http://62.152.67.70/cryobank/login.jsp, accessed on 6 September 2024), was used as a negative control. According to genomic analysis, the strain belongs to the *Bacillus mycoides* species. The registration number is RCAM06119.

### 2.5. DNA Extraction and Quality Control

Total DNA was extracted according to the protocol described by Romanenko et al., 2023 [50], with slight modifications. First, the bacterial culture was grown for 12 h in a liquid Spizizen nutrient medium [51,52] with aeration at +28 °C and then centrifuged at +4 °C. The cells were then extracted from the nutrient medium by washing the culture three times with buffer (EDTA 0.01 M, NaCl 0.15 M pH 8.0). Next, 500 μL of the buffer and 15 μL of RNase A (10 mg/mL) were added to the washed cells. The samples were incubated for 60 min at +37 °C with the addition of 10 μL of lysozyme (20 mg/mL), as well as 5 μL of mutanolysin (1 mg/mL). Then, 3 μL of proteinase K was added to the cell lysate and incubated for 30 min at +37 °C. After this, 10% sodium dodecyl sulfate was added in a volume of 50 µL, and the mixture was again incubated for 10 min at +65 °C. After incubation with 10% sodium dodecyl sulfate, DNA purification was carried out with the addition of 200 μL of Protein Precipitation Solution (Qiagen, Venlo, the Netherlands), gentle mixing, and incubation on ice for 60 min. The samples were then centrifuged at 16,000× *g*, and the supernatant was transferred to clean tubes. DNA was precipitated with isopropanol, washed three times with 70% freshly prepared ethanol, and finally dissolved in 30 μL of Tris-EDTA (TE) buffer (pH 8.0). The eluate was left at 4 °C for 18–24 h to dissolve.

The concentration of the extracted genomic DNA was measured using a Qubit^®^ 3.0 fluorometer and the Qubit dsDNA BR Assay kit (Life Technologies, Eugene, ON, USA). To evaluate contamination with proteins, phenol, or other substances, absorbance ratios at 260 nm/280 nm and 260 nm/230 nm were used. The cutoff of ≥1.8 was set as an indicator of the sample’s purity. These measurements were conducted on a CLARIOstar Plus multimodal reader (BMG Labtech, Ortenberg, Germany). The DNA samples underwent further qualitative and quantitative analysis through electrophoresis on 1% agarose gel stained with 0.002% ethidium bromide, and the results were compared to the λ DNA/HindIII marker (Thermo Fisher Scientific, Inc., Waltham, MA, USA).

### 2.6. Genome Assembly and Annotation

The whole-genome sequencing was carried out on the Illumina HiSeq X platform in paired-end mode with a read length of 2 × 100 bp by Novogene Co., Ltd. (Beijing, China). Adapter sequences were removed with fastp v0.23.2 [53], followed by visual inspection for quality control on the trimmed short reads using the program’s reports. An additional assessment was performed with FastQC v0.12.1 [54] to prove uniform distributions of quality scores and GC content. Trimmed and quality-checked short reads were assembled with SPAdes v3.15.4 [55] in the “--careful” mode with the list of k-mers for read corrections of 21, 33, 55, and 77. The general properties of the assembly were inspected by QUAST v5.2.0 [56]. To verify completeness in terms of taxonomic attribution, we calculated the percentage of single-copy orthologs included in the “Bacillales_odb10” and “Bacilli_odb10” databases using BUSCO v5.4.2 [57]. Completeness and contamination levels were evaluated with the CheckM v1.2.2 [58] tool in the “lineage_wf” mode. The assembly was deposited in NCBI databases under accession numbers PRJNA1127832 (BioProject), SAMN42021048 (BioSample), SRR29530170 (SRA), and GCF_040567675.1 (Genome).

To perform gene annotation, we first calculated pair-wise ANI (average nucleotide identity) values with all genomes belonging to the order of Bacillales and deposited in the RefSeq database [59] using fastANI v1.33 [60] with a k-mer size of 16 specified. Only non-anomalous assemblies—i.e., devoid of frameshifted proteins, showing low contamination levels, and matching the claimed taxonomic attributions—were selected. To improve gene identification, we reconstructed the gene prediction model with Prodigal v2.6.3 [61] based on the concatenated sequence of the 10 closest reference genomes with the highest ANI estimates. The model was then utilized for gene annotation with Prokka v1.14.6 [62] launched with the following parameters: “--addgenes”, “--genus ‘Bacillus’”, “--gffver ‘3’”, and “--usegenus”. To increase the quality of annotation, we created an in-house database of protein sequences from reference proteomes included in the BUSCO database of strains belonging to the order of Bacillales in addition to the default Prokka database.

After finishing the general annotation, we searched for specific loci contributing to the biological activity of the strain. Toxin-encoding genes were mined with BtToxin_Digger v1.0.10 [63] and CryProcessor v1.0 [64]. The former program was applied to identify a broad range of toxins while the latter was utilized for the specific identification of *cry* loci since certain representatives of the species could synthesize Cry toxins [16]. The host range for known homologs of the toxins was taken from the BPPRC (Bacterial Pesticidal Protein Resource Center) database [65] (https://www.bpprc-db.org/, accessed on 24 July 2024). The validity of the metadata was checked by examining the references provided by the resource. To identify BGCs, antiSMASH v7.0 [66] and DeepBGC v0.1.30 [67] were utilized. The antiSMASH utility was applied to reveal known BGCs. The underlying algorithm of DeepBGC is based on the neural network, thus allowing the prediction of putative clusters potentially missed by the approach based on the similarity between sequences. Therefore, we combined these methods for better characterization of the strain from a genomic perspective.

### 2.7. Descriptive Analysis of Reference Genomes

To understand if and how our strain differs from the closest strains within the *B. mycoides* species, we extended the set of examined genomes using the ANI-based approach described above. We gathered 50 reference genomes from the same dataset of all genomes belonging to the order of Bacillales. We then gathered the metadata of these strains from NCBI RefSeq [59] and BioSample databases [68]. To summarize the genomic features within the dataset, we performed a downstream analysis analogous to what was made to annotate strain b12.3 after the reads’ quality checks and assembly.

All the plots were visualized with the ggplot2 v3.3.5 package [69]. To reconstruct the phylogeny, we first built the pangenome with Panaroo v1.2.8 [70] with a 95% identity threshold to select core genes and the “--remove-invalid-genes” parameter specified. MAFFT v.7 [71] was used for independent alignments of the extracted core genes, which were then concatenated in a single sequence for further analysis. The optimal evolutionary model was picked in agreement with the BIC (Bayesian information criterion) estimates provided by ModelTest-NG v0.1.7 [72] launched in the “ml” mode. We extracted core SNPs (single-nucleotide polymorphisms) from the alignment using SNP-sites v2.5.1 [73] and utilized RAxML-NG v1.1.0 [74] with 1000 bootstrap replicates to reconstruct the ML (maximum likelihood) phylogeny specifying the best evolutionary model.

## 3. Results

### 3.1. Morphological Characterization of b12.3 Strain

**Figure 2:**
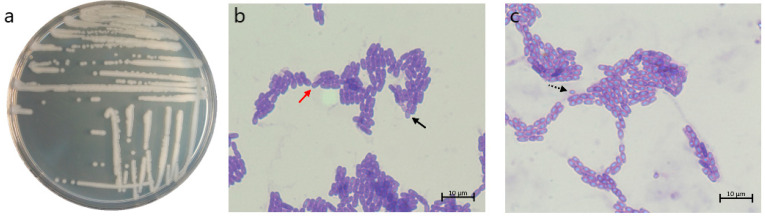
The morphology of the b12.3 strain’s colonies after 24 h of cultivation on LB medium (**a**) and transition from vegetative to sporulating stage after 24 h (**b**) and 48 h (**c**) of the growth on CCY medium stained with Coomassie Brilliant Blue (1000 magnification). The red solid arrow shows the vegetative cell, the black solid arrow shows the spore inside the cell, and the black dotted arrow shows the spore.

We first inspected the morphology of the isolate from the soil sample. The strain’s colonies after 24 h of cultivation on the LB nutrient medium were round, flat, matte, yellowish-white colonies with undulate margins and rough surfaces (Figure 2a). On the first day of cultivation on the CCY medium, it formed short rod-shaped vegetative cells collected in short chains with centrally located spores (Figure 2b). On the second day of cultivation, the sporulation stage with clearly visible elliptical spores was observed (Figure 2c).

### 3.2. Strain Identification by the gyrB Gene Sequence

We first assessed the taxonomic attribution of the isolated strain by sequencing the *gyrB* gene chosen as a common marker for taxonomic delineation. The gene of strain b12.3 was found to be of the highest similarity (99.76%), with the strain BPN37/2 (NCBI GenBank accession number CP036004.1) attributed to the *Bacillus mycoides* species. Such a high score exceeds the average rates within the *B. cereus sensu lato* group [75], thus allowing us to consider the strain b12.3 *B. mycoides*, despite its morphology without typical filamentous colonies of *B. mycoides* [76].

### 3.3. Screening of Strain b12.3’s Antagonistic Activity Against Bacterial Pathogens of Agricultural Crops

The strain’s activity was screened against 13 bacterial pathogens of agricultural crops (Table 2). Of the 13 strains, growth suppression during cross-streak screening was detected for 5 pathogen strains.

**Figure 3:**
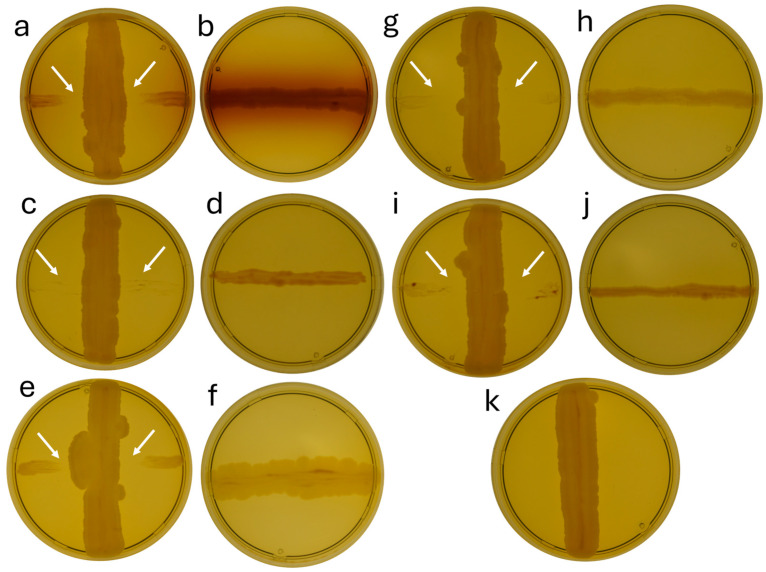
The figures show pairs of Petri dishes demonstrating the growth of pathogens with and without the addition of a vertical streak of the b12.3 strain: (**a**,**b**) *X. campestris* 23; (**c**,**d**) *X. campestris* 01002; (**e**,**f**) *C. michiganensis* 5351; (**g**,**h**) *P. atrosepticum* 01726; (**i**,**j**) *P. atrosepticum* 5128; (**k**) single streak of the b12.3 without pathogens. The white arrows indicate the zones of pathogen growth inhibition.

Figure 3 demonstrates that strain b12.3 exhibits a visible antagonism with *X. campestris*, *C. michiganensis,* and *P. atrosepticum* (Figure 3c,d). The inhibitory effect on *X. campestris* 01002 and *P. atrosepticum* 01726 caused almost complete suppression of pathogen growth. In contrast, for *C. michiganensis* 5351, *X. campestris* 23, and *P. atrosepticum* 5128, the inhibition zone ranged from 5 to 9 mm. Thus, we detected the bactericidal activity of strain b12.3 against five strains of agricultural crop pathogens.

### 3.4. Testing the Fungicidal Activity of the Strain Against Fungal Plant Pathogens

Fungicidal activity was tested against eight phytopathogenic fungi. To perform bioassays, we used four strains of genus *Fusarium*, two strains of *A. solani*, and *B. sorokiniana* with *Botrytis* sp. as well (Table 3).

**Figure 4:**
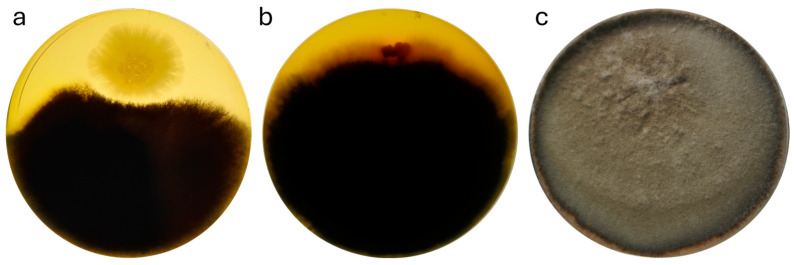
Fungicidal effect of strain b12.3 against the plant pathogen *A. solani* 4601. (**a**) Fungicidal activity after 10 days of co-cultivation of the tested strain with the plant pathogen fungus. (**b**) Negative control on the tenth day of co-cultivation of the *B. mycoides* strain c8.2 with *A. solani* 46011. (**c**) Normal growth of *A. solani* 46011 after two weeks of cultivation on PDA nutrient medium.

Having visually inspected the co-cultivated Petri dishes, we noticed a noticeable inhibition of *A. solani* 46011 by strain b12.3 with the size of the inhibition zone exceeding cm (Figure 4a). It is worth noting that another *B. mycoides* strain, c8.2, also deposited in our collection, lacks the respective activity (Figure 4b). The strength of strain b12.3’s antagonism is more visible if we match the image of the co-cultivation with that of the normal growth of *A. solani* 46011 on the PDA medium (Figure 4c).

According to the obtained results, we have identified the fungicidal activity of the b12.3 strain against *A. solani*, an important phytopathogenic fungus infecting many plant species [77,78,79,80]. We, therefore, might consider the strain a potential biocontrol agent.

### 3.5. The Genomic Properties of the Strain b12.3

#### 3.5.1. The Basic Features of the Genome Assembly

To reveal genomic loci contributing to the strains’ experimentally verified antipathogenic properties and predict other putative activities, we performed whole-genome sequencing. The draft genome consisted of 83 contigs with a total length of 5,773,031 b.p. The genome coverage reached 188 reads per base pair on average. The GC content reached 35.14%. The assembly was characterized by low contamination (0.65%), and it almost fully represented the genome content (99.01% completeness) as well. The completeness and low misassembly rate were further proved by the software BUSCO v5.4.2 [57] with the bacillales_odb10 database specified, indicating that 445 out of 450 (98.9%) indicator loci were single-copy orthologs. The annotated genome contained 5742 CDSs (coding sequences), with 700 of them (12.2%) coding for proteins of unknown function (Data S1). Taken together, the draft assembly of strain b12.3 sufficed to provide near-complete genome content with a low contamination level.

#### 3.5.2. Biosynthetic Gene Clusters and Insecticidal Loci Detected in the Strain b12.3

We went on to trace agriculturally important loci in the genome of the studied strain, focusing on genes encoding BGCs to examine its metabolic capabilities. The tool antiSMASH v7.0 reported 13 BGCs (Table S2), while DeepBGC v0.1.30 (Table S3) reported 38 putative BGCs. Notably, two known clusters with the highest similarity were those responsible for producing Bacillibactin and Petrobactin corresponding to the similarity scores of 85.71% and 100%, respectively. The DeepBGC utility could predict the probable functional action of the synthesized compounds. These included 30 bactericidal BGCs and one cluster with a joint bactericidal and fungicidal effect, which might explain its experimentally evaluated ability to inhibit the growth of fungal and bacterial plant pathogens. Two antiSMASH-detected BGCs were attributed to antibiotic activity as well (Table 4). Therefore, the genetic landscape of strain b12.3 indicates its possible insecticidal efficacy and sheds light on its fungicidal and bactericidal activities stemming from the arsenal of BGCs. To explore whether the genomic composition of the strain explains its effect on the bacterial pathogens and to assess how unique its gene content is, we went on to compare its genomic makeup with deposited assemblies.

Having located BGCs, we proceeded with predicting the strain’s pesticidal potential by revealing virulence loci. The strain is unable to synthesize Cry toxins as revealed by CryProcessor v1.0 [64]. However, it could produce App4Aa1, Tpp78Ba1, and Spp1Aa1 (Table 5). The two latter toxins are suspected to exhibit activity against the orders of Hemiptera, Lepidoptera, and Blattodea, thus reflecting the possible insecticidal potential of the isolate to be tested further.

### 3.6. Genomic Comparisons with Closest Reference Assemblies

#### 3.6.1. Reference Strain Selection and Description

**Figure 5:**
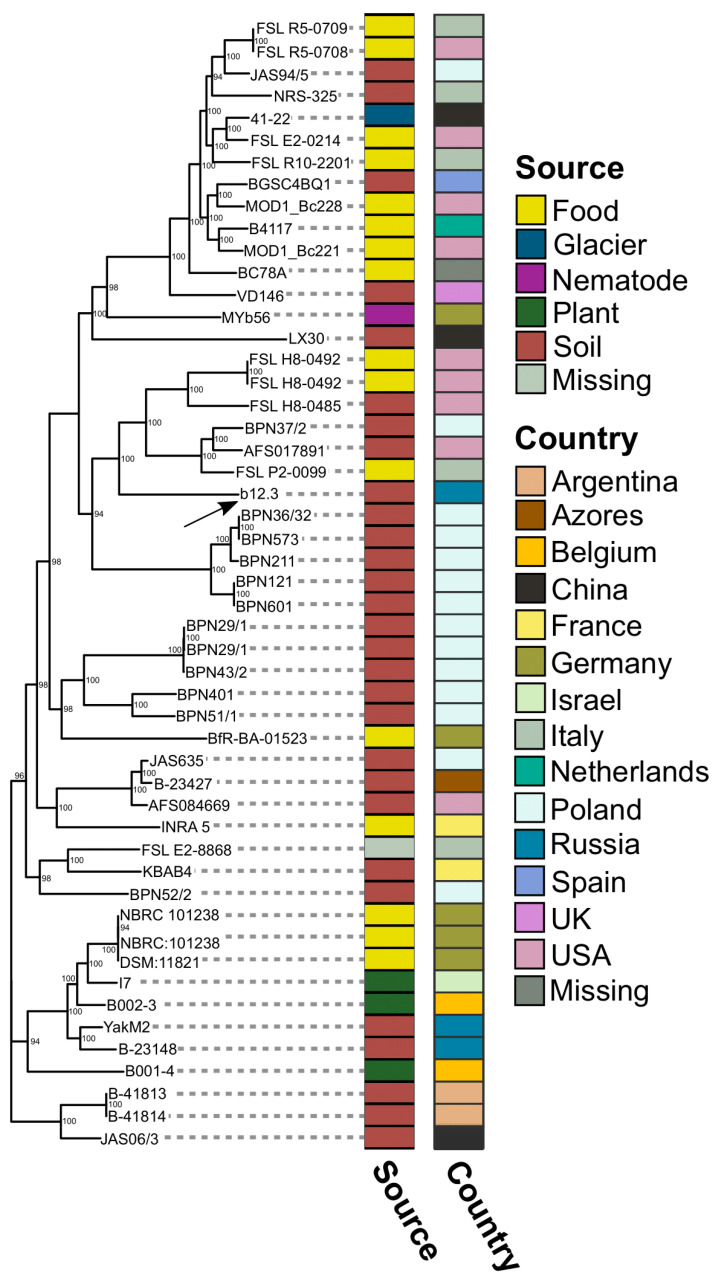
Maximum likelihood phylogeny based on core SNS in the 50 closest reference genomes coupled with the assembled genome of the strain b12.3. The numbers near the branches of the tree represent support estimates using 1000 bootstrap replicates. The black arrow points to the strain b12.3. The right stripes adjacent to the tree leaves represent the isolation source and geographic origin of the strains.

We opted for the 50 closest genomes (Table S4), with the vast majority of them being attributed to *B. mycoides* (Table S5), thus allowing us to assign the strain b12.3 to this species, despite its atypical morphology. The strains were isolated from five sources, and the most abundant of them were food-borne microorganisms and soil dwellers. Regarding geographic origin, there were 14 countries; two isolates were from Russia. To characterize phylogenetic relationships, we reconstructed ML phylogeny based on core SNPs (Figure 5). Notably, the studied strain fell into one category, with genomes assigned to Poland and the USA, whilst two reference strains (YakM2 and B-23148) attributed to Russia were grouped in a single two-leaf branch in the tree (Figure 5). The phylogeny showed other strains from the same country to be dispersed among different clades. Therefore, the data suggested that geography per se, in general, does not reflect relationships between *B. mycoides* genomes. On this account, we proceeded with an examination of whether phylogeny corresponds to the presence/absence patterns of BGCs and virulence factors.

#### 3.6.2. The Distribution of BGCs and Toxin-Encoding Loci in the Genomic Dataset

**Figure 6:**
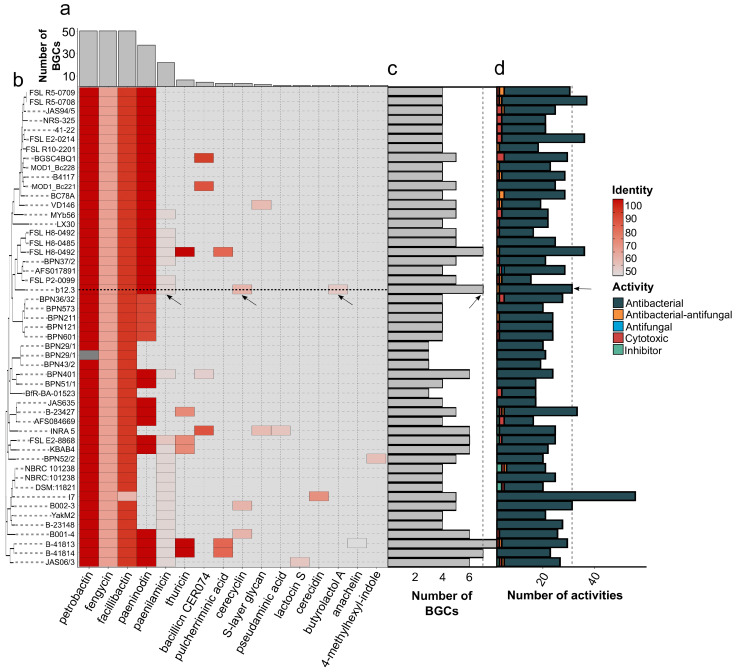
The presence/absence patterns of BGCs in the examined dataset. (**a**) The abundance of BGCs found in the studied genomes. (**b**) A heatmap displaying the distribution of BGCs in the dataset identified with antiSMASH v7.0 [66]. The rows are ordered in agreement with the reference ML phylogeny, and the tiles are colored according to the identity with the known cluster. The black arrow and grey dotted line highlight strain b12.3. (**c**) The total amount of known BGCs present in the genomes of the respective strains. (**d**) The number of putative BGCs detected with the DeepBGC v0.1.30 [67] utility. The colorized blocks within the bar plot represent DeepBGC-predicted biological activities of the metabolites.

We proceeded with a comparative genomic analysis of the closest strains starting with summarizing BGCs in their genomes (Tables S6 and S7). All of the isolates bore metabolic clusters responsible for the biosynthesis of bacillibactin, fengycin, and petrobactin (Figure 6a, Table S8). Two other abundant clusters were related to paeninodin (38 BGCs) and paenilamicin (22 BGCs). There were 16 compounds in the aggregate (Table S8). Similar to insecticidal loci, there were no explicit links between groups of the reference tree and the presence of the BGCs (Figure 6b). It is noteworthy that our strain was enriched with known BGCs (Figure 6c) and putative biological activities (Figure 6d) in comparison to other isolates, highlighting its promising applicability in practice.

**Figure 7:**
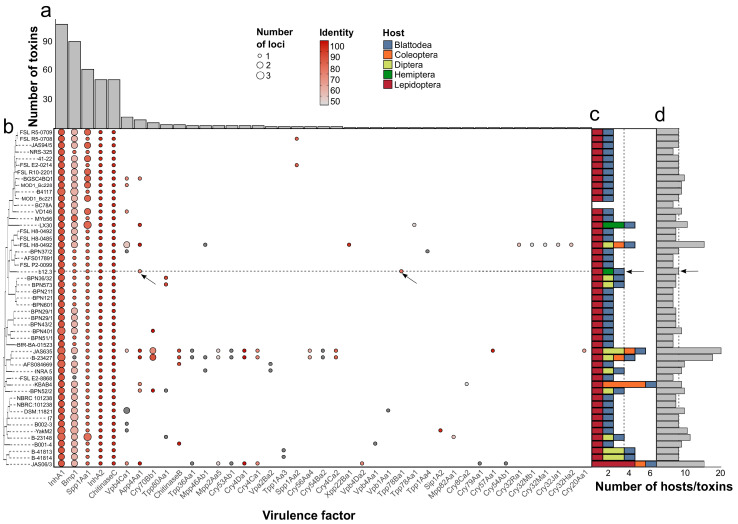
The distribution of loci coding for virulence factors in the reference dataset. (**a**) The total number of certain virulence loci in the genomes. In the bar plots, paralogs are considered as well. (**b**) The presence/absence patterns of virulence genes in 51 *B. mycoides* genomes. The heatmap is ordered as per the reference phylogeny. The size of the dots is proportional to the number of paralogs, whilst the colors represent the identities with known homologs. The strain b12.3 is marked with the black arrow. (**c**) The predicted host orders for each strain. The data were taken from the BPPRC database [66] for the respective homologs. The size of the bars illustrates the number of species from a certain order. (**d**) The total number of insecticidal genes in the analyzed genomes regarding paralogs.

After examining the insecticidal genes, we continued with mining the virulence factors (Tables S5 and S6). All genomes without exceptions housed loci encoding for InhA1/InhA2, Bmp1, Spp1Aa1, and ChitinaseC (Figure 7a,b, Table S9). There were 41 distinct virulence determinants found in total. Notably, multiple entities were encoded by several paralogs with different identity estimates with known homologs. The most frequent of them were genes coding for InhA1 (109), Bmp1 (91), and Spp1Aa1 (62) (Figure 7a, Table S9). Only seven strains contained *cry* genes of various families, with most of them being present in one copy (Figure 7a,b, Table S9). There were five host orders putatively susceptible to the bacteria (Figure 7c). Importantly, the strain b12.3 was predicted to exert a toxic effect on three orders, thus probably being of higher insecticidal potency than the large portion of other isolates, while the total number of pesticidal loci reaching eight was close to the average (Figure 7d). The presence/absence patterns of the virulence genes seem to be largely independent of phylogeny except for genomes of the same strains (Figure 7b).

### 3.7. Identifying Determinants of B. mycoides b12.3 Specificity Using Available Metadata

To explain the correspondence between the activities of the isolated strain and its observed bactericidal and fungicidal activities, we collected available data for performing comparative analysis. We first went on to analyze the closest reference isolates to assess the similarity between them and b12.3 in terms of biological activities. Nevertheless, according to the available data, none of these strains were tested against bacteria and fungi (Table S10). The works mentioning these isolates are mostly focused on examining virulence and safety [84,85,86] since *B. mycoides*, being a member of the *B. cereus sensu lato* group, is often isolated from food sources [87].

We then gathered data on the species regarding fungicidal/bactericidal effects (Table S11). Surprisingly, the bactericidal and fungicidal potential of *B. mycoides* remains poorly studied. Certain reports describe contrasting activities. For example, *B. mycoides* PR04 is capable of inhibiting Gram-positive bacteria, such as *Staphylococcus aureus* and *Listeria monocytogenes,* albeit exhibiting no activity against Gram-negative microorganisms, namely, *Pseudomonas aeruginosa* and *Salmonella enterica* serovar Typhimurium [88]. At the same time, *B. mycoides* DFC 1 affected both groups of bacteria [89], while *S. aureus* remained intact when cultivated with strain QAUBM19 [90]. Similarly, isolates MF377573 and MF377556 exerted effects on the fungus *Fusarium euwallaceae* but not *Graphium* sp. [91]. It is noteworthy that there are strains able to kill *F. oxysporum* [25,92] and *B. cinerea* [93,94], unlike the isolate b12.3 described here. Unfortunately, there are only a few genomes of these bacteria, and the spectrum of produced secondary metabolites is unknown as well (Table S11), which does not allow us to perform a thorough comparative genomic analysis.

**Figure 8:**
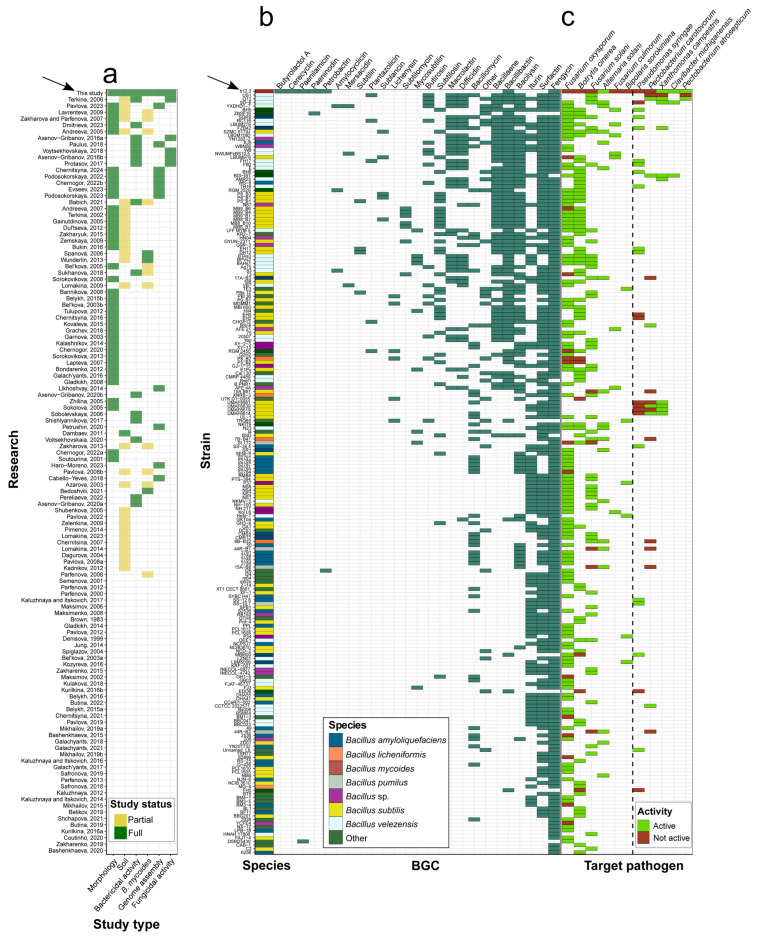
Visualization of the data presented in articles devoted to examining the microbiome of Lake Baikal and surrounding territories and in studies aimed at screening antiphytopathogenic activities of strains containing BGCs (biosynthetic gene clusters) found in *B. mycoides* strain b12.3. (**a**) The types of research articles related to bacteria from the Lake Baikal region. Plotted on the *y*-axis are the references [4,5,6,7,8,9,10,11,13,14,15,95,96,97,98,99,100,101,102,103,104,105,106,107,108,109,110,111,112,113,114,115,116,117,118,119,120,121,122,123,124,125,126,127,128,129,130,131,132,133,134,135,136,137,138,139,140,141,142,143,144,145,146,147,148,149,150,151,152,153,154,155,156,157,158,159,160,161,162,163,164,165,166,167,168,169,170,171,172,173,174,175,176,177,178,179,180,181,182,183,184,185,186,187,188,189,190,191,192,193,194,195,196,197,198,199], while the *x*-axis represents the experimental approaches incorporated in the current work, which is marked with a black arrow on the graph. The tiles display whether any of the procedures were applied in the respective articles. The colors indicate the level of similarity with our study, i.e., if the representative of the genus *Bacillus* of unknown species, attributions of bottom sediments but not soil were described, then the status is considered partial. Short summaries of the included studies are presented in Table S12. (**b**) The distribution of BGCs in the strains from the *Bacillaceae* family exhibits the ability and/or inability to inhibit plant pathogens tested in the current research. The adjacent left strip visualizes the species to which a certain strain belongs. The species and BGCs with fewer than three representatives are grouped into the “Other” category. The black arrow highlights the strain b12.3 isolated in the current study. The data for the graphical representation are taken from Table S13. (**c**) The spectrum of activities against target species on which the isolates plotted on the *x*-axis were tested. Tiles are colorized according to the observed activity (present/absent). The vertical dashed line delineates fungal (left) and bacterial (right) phytopathogens.

Given that direct comparisons with close relatives and species’ representatives are not informative enough due to the lack of data, we proceeded with the analysis of bacteria residing in the Lake Baikal region. To reveal how the geographic region and/or ecological niche where the strains were isolated influenced the range of affected bacteria/fungi, we collected the data on microbiological studies of Lake Baikal and surrounding territories (Figure 8a, Table S12). While there are multiple research items related to the topic, the present knowledge is too scarce to draw any conclusions. The vast majority of studies aimed to describe either biochemical features of microbial communities [95,96,97,98] and/or general taxonomic distributions of microorganisms from different sample sites [99,100,101]. Several research efforts have been made to characterize the bactericidal [102,103] and fungicidal [104,105,106] effects of the isolated bacteria. At the same time, reports on *B. mycoides* remain scarce and do not include genome assembly and screening for bactericidal/fungicidal activities [13,14,15]. Interestingly, while the representatives of the *Bacillus* genus, including *B. mycoides*, are frequently found in bottom sediments [14,15,107,108,109], soil from the islands and shores of Lake Baikal remains almost unexplored [110]. To sum up, there are not enough data to prove any relationship between geography and the biological activities of *B. mycoides* isolates from the Baikal region, which highlights the importance of our study and emphasizes the necessity to conduct research employing multiple approaches (morphological description, bioactivity screening, and genome analysis) of soil isolates from the Lake Baikal zone with fundamental and practical objectives.

Since the current body of research is not sufficient to reveal the determinants of bioactive properties for representatives of *B. mycoides*, we proceeded with inspecting BGCs identified in bacteria tested against the same phytopathogens as have been screened here (Figure 8b,c, Table S13). The BGCs with the highest frequency were those related to the synthesis of fengycin, Surfactin, Iturin, Bacilysin, Bacillibactin, and Bacillaene (Figure 8b). The species mostly belonged to the Bacillus genus with certain other members of the Bacillaceae family (Table S13). Notably, BGGs for the production of cerecyclin and butyrolactol A were unique for b12.3. Phytopathogenic fungi were tested more often than bacterial pathogens. The most abundant amount of data belonged to *F. oxysporum*, *B. cinerea*, and *F. solani* as well as *P. syringae* and *P. carotovorum* (Figure 8c). Although the data are still limited, we observe that the ability to suppress certain fungal phytopathogens relies on the joint presence of fengycin and iturin/surfactin.

Taken together, we found that not only does our strain contain more BGCs than its closest references on average but its genome also hides certain insecticidal loci, making the strain b12.3 a promising candidate as an efficient and multi-functional biocontrol agent. While possessing strong fungicidal activity towards *A. solani* only, it was able to inhibit a wide range of bacterial species.

## 4. Discussion

In this research, we isolated *B. mycoides* b12.3 and explored its biological activities, conducted a comprehensive genome survey to assess its antagonism towards plant pathogens, and provided a comprehensive description of its morphology and genomic characteristics. The morphological features of the strain were quite unusual. Normally, wild-type *B. mycoides* representatives form filamentous colonies curving clockwise or counter-clockwise on agar plates [76]. The b12.3 strain fell short of this canonical pattern, displaying round compact colonies instead. Spontaneous mutants with similar morphology were previously reported for *B. mycoides* [76]. The observations made suggested that the regulation of cell separation appears to be modified in mutants that have lost their regular rhizoidal colony shape [76]. Still, the exact genes or gene clusters delineating this morphotype are unknown so far.

By using the co-cultivation technique, we showed the capabilities of strain b12.3 to inhibit the growth of plant pathogens of bacterial and fungal nature. According to bioassays, strain b12.3 was found to have a visible effect on the agents of plant diseases, including bacteria (*X. campestris*, *C. michiganensis*, and *P. atrospecticum*) and the fungus *A. solani* (Table 3 and Table 4). Being a plant growth-promoting bacterium, *B. mycoides* could elicit systemic response [200] and oxidative burst [201], boosting the immune reactions of the host. In addition, it could directly compete with pathogens. While bactericidal properties of the species have not been studied extensively, in vitro experiments have revealed that certain *B. mycoides* strains act as antagonists of food-borne human pathogens [90,202]. In contrast, the fungicidal activity of the species is much better understood. When analyzed in greenhouse conditions, it suppressed *Botrytis cinerea* [94] and *Pythium aphanidermatum* [23]. Analogous results were obtained while studying its competition with *Phytophthora cinnamomi* [91], *Colletotrichum gloeosporioides* [203], *F. oxysporum* [25], and *Sclerotinia sclerotiorum* [204]. Therefore, the activities found for strain b12.3 have not been described elsewhere for *B. mycoides,* implying that it is a novel prospective biocontrol agent.

To uncover the genetic fundament upon which rests the strain’s efficacy in combating plant pathogens and to predict other biological features of high potential, we assembled and annotated its genome. Genome mining revealed *B. mycoides* b12.3 to harbor a wide array of known and putative BGCs orchestrating the metabolism of bioactive compounds (Figure 6b,c, Table 4). One such instance is petrobactin, a siderophore of the catecholate’s chemical nature. Initially perceived as a virulence factor required for maintaining the course of *B. anthracis* infection through accumulating hosts’ iron [205], it was later detected in various strains within the *Bacillus* genus [206]. In the case of plant-associated isolates, the chelating properties of siderophores are, in fact, beneficial for their hosts as iron becomes sequestered from plant pathogens [207]. Another catechol-type siderophore was bacillibactin, which acts like petrobactin, enabling the compartmentalization of iron and hence providing a competitive advantage [208]. For example, it was reported to suppress the growth of diverse MDR (multidrug-resistant) human pathogens [209], *P. syringae* pv. *tomato* [210], *X. campestris*, and *C. michiganensis* [211]. Apart from siderophores, we found that the strain harbors loci enabling the biosynthesis of lipopeptides by NRPS. Of them, fengycin is a prominent fungicidal moiety that exhibited strong activity against *F. solani* [212] and *A. solani* [213]. Paenilamicin is a chemical hybrid of non-ribosomal peptides and polyketides produced by NRPS and PKSs (polyketide synthases) first isolated from *Paenibacillus larvae*, a bee pathogen [214]. Not only does it increase competitive ability through a wide range of activities against fungi and bacteria, but it also exhibits a direct cytotoxic insecticidal effect on bees [215]. However, we should note that the respective cluster in the strain b12.3 genome was of the lowest similarity, thus meaning that the bioactivity of the metabolite would differ significantly. The Cerecyclin gene cluster was discovered recently, and the properties of the compound have been less studied so far; however, it displayed a strong inhibitory effect on Gram-positive bacteria [216]. Paeninodin is a lasso peptide belonging to the class of RiPPs (ribosomally synthesized and post-translationally modified peptides) [217]. However, showing no inhibitory effect on several bacterial species while applied per se [217], the substance was found in bioactive *Paenibacillus* plant growth-promoting strains which do not exclude its possible activity when present in the mix of bactericidal and/or fungicidal metabolites [218]. Unlike the compounds mentioned above, butyrolactol A represents a polyketide with an uncommon tert-butyl starter. It was proven to be a strong fungicide with a wide host range [219,220].

In addition to enrichment with BGCs, the resulting assembly suggests that the b12.3 isolate is capable of forming within-spore and vegetative insecticidal toxins (Figure 7b, Table 5). The vegetative toxin Spp1Aa, formerly termed sphaericolysin, was discovered in *Lysinibacillus sphaericus* A3-2 [83]. It belongs to the family of cytolysins with a cholesterol-binding motif enabling damage to ganglial cells in *Blattela germanica* and *Spodoptera litura* [83]. The App4Aa toxin, unlike most of the crystalline pore-forming counterparts, structurally resembles hemolysins of the HlyE family, consisting of alpha helices instead of beta sheets [221]. Before the domain-wise reclassification of pesticidal proteins by Clickmore et al. [65], these toxins were recognized as the Cry6 group, with certain representatives causing pore formation in nematodes [221]. Nevertheless, App4Aa was proven to affect *Plutella xylostella* [222]. Another previously affiliated with Cry toxins protein Tpp78Ba is a part of the Tpp family encompassing proteins with pore-forming Toxin_10 and Ricin_B_lectin domains [82]. The representatives of this protein class were reported to hold substantial mosquitocidal activity especially when acting synergistically with Cyt1 toxins [223,224]. However, App4Aa (formerly known as Cry78Ba) was displayed to induce high mortality in *Laodelphax striatellus* when administrated by feeding [82].

The comparative analysis with the phylogenetically closest genomes of *B. mycoides* strains demonstrated that all of them carried non-specific virulence factors with pesticidal effects (Figure 7b). Those loci included genes coding for chitinase C conducting disintegration of the peritrophic membrane [225], InhA1/InhA2 metalloproteases responsible for proteolytic degradation of antibacterial peptides excreted by host immune cells [226], and Bmp1 exerting toxicity to nematodes by degrading intestinal tissues [227]. Although generally considered non-specific, the accumulated evidence supposes these proteins contribute to host preference [228]. Several strains were able to produce three-domain Cry toxins (Table S6). While being relatively rare, it is known that *B. mycoides* representatives synthesize crystals with Cry toxins encrusted. These strains, previously assigned to a non-taxonomic category *B. thuringiensis*, are now termed biovar Thuringiensis within the *B. mycoides* genomospecies [16]. It is worth noting that genomes harboring *cry* loci generally carry more *cry* types and other insecticidal genes, likely reflecting that the propagation of Cry-encoding genes is frequently associated with the HGT (horizontal gene transfer) phenomena leading to the acquisition of genetic material [229]. Furthermore, the presence of Spp1Aa homologs coupled with other secretive and spore-encrusting toxins suggests *B. mycoides* holds promising pesticidal capabilities. In spite of the findings observed, we should mention that the presence of insecticidal loci, being identified with bioinformatic procedures, might not indicate the presence of in vitro activity as well as the efficacy when tested in field conditions. Therefore, further research is needed to assess the predicted pesticidal potential of *B. mycoides* strains.

Regarding the BGCs present in the dataset, we discovered that all the genomes carried clusters related to the production of siderophores, namely, bacillibactin and petrobactin, as well as lipopeptide fengycin (Figure 6b). Given that these clusters were present in our strain as well, one could expect similar activities. We should note the notable observation that, despite possessing fengycin, known for inhibiting action against *F. oxysporum* [230], strain b12.3 was able to suppress the growth of *A. solani* while exerting no effect on *F. oxysporum*. We propose several hypotheses explaining this result. We suggest that the spectrum of activities is delineated by (i) geographic origin and adaptation to certain pathogens residing in the occupied ecological niche; (ii) phylogenetic position of strains; and (iii) the combination of BGCs present in the genome.

After performing an in-depth analysis of current studies, we have to admit that there are not enough data to verify the impact of geography and phylogeny on the specificity of beneficial strains on phytopathogens. Having inspected the distributions of BGCs and affected pathogens (Figure 8b,c), we noticed several patterns probably determining the antagonistic action. For instance, the ability to repress *F. oxysporum,* which was intact when co-cultivated with our strain, is observed in isolates producing fengycin in combination with surfactin and/or iturin [231,232,233,234], while those synthesizing fengycin only were unable to cause inhibition of the phytopathogen [235,236,237]. Nevertheless, there are examples of active non-producers [218,238,239] of this mix of metabolites and unactive producers as well [235,240,241]. The patterns related to the activities toward *B. cinerea*, *F. solani*, and *A. solani* fit in the trend described above. Notably, strains 44R-B1 and 44R-B2, belonging to the *Bacillus pumilus* species and containing BGCs related to fengycin but not surfactin and iturin, were shown to be active against *A. solani* and exerted no effect on *F. solani* as was the case with our strain [242]. The evidence on bactericidal effects is too limited for us to inspect common patterns. Given all the data mentioned above, we can conclude that the spectrum of activities in agriculturally important isolates is certainly associated with the combinations of produced secondary metabolites; however, to find the exact associations for predicting activities by mining genomes for BGCs, further research employing genomic and experimental techniques is required.

To assess the practical applicability of strain b12.3, we compared its properties with patented strains and commercial biopreparations (Tables S13 and S14). The most commonly used commercial strain of *B. mycoides* is the isolate J (BmJ), an effective biocontrol agent against *Cercospora* sp. causing leaf spot diseases [200]. In general, the amount of patented *B. mycoides* strains is small in contrast to other members of the genus, although certain strains were claimed to reduce the plant lesions caused by *Alternaria alternata* [243], *Sphaerotheca fuliginea* [244], *Erwinia aroideae* [245], etc.

Summarizing the strains that inhibit the same pathogens as b12.3 revealed two major groups of patented bacteria, namely, those with predominantly fungicidal or bactericidal characteristics (Table S14). Our strain belonged to the second group resembling *Pseudomonas* sp. in terms of their activities [246,247]. It also partially resembles commercial biopreparations based on *Bacillus* strains to combat *Xanthomonas campestris* and *Clavibacter michiganensis* (Table S15). All things considered, *B. mycoides* strain b12.3 has an agricultural potential as an agent of wide action comparable with registered preparations. Moreover, the joint application of b12.3 with strains producing iturins and surfactins could result in a formulation of broad action against bacterial and fungal pathogens.

## 5. Conclusions

*B. mycoides* is prevalent in various ecosystems, especially in soil, although its biology, genomics, and ecology remain understudied. In this research, we provided insights into its bactericidal and fungicidal activities by combining co-cultivation methods and NGS to thoroughly examine strain b12.3. To the best of our knowledge, the strain described in this research is the first *B. mycoides* isolate found in Olkhon Island in Lake Baikal. The isolate exhibited the ability to inhibit the growth of fungal and bacterial plant pathogens yet unreported in studies related to *B. mycoides*. The genomic survey revealed the b12.3 genome to be enriched with metabolic clusters as compared to its closest reference assemblies. Genome mining determined that a distinctive combination of BGCs could explain the results obtained as well. Another notable finding is the prevalence of pesticidal loci among *B. mycoides,* implying their potential to control pests. To sum up, leveraging full genome sequence analysis coupled with a comprehensive comparison with deposited assemblies provides insights into which loci delineate the biological activities of the strains. Therefore, applying NGS with a special focus on featured loci related to agriculturally important properties could ease the screening for promising biocontrol agents.

## Figures and Tables

**Table 1:** List of plant pathogens used in this work.

Pathogen Type	Species	Strain(s)/Pathovar(s)	Provided by *
	*Pseudomonas* sp.	8949	Dr. A.M.Lazarev, FSBSI VIZR
Bacteria	*Pectobacterium atrosepticum*	5128	
	*Clavibacter michiganensis*	5351	
	*Xanthomonas campestris*	*23*	
	*Pseudomonas syringae*	03278 and 00904	RCAM, ARRIAM
	*P. atrosepticum*	01726 and 01000	
	*Pectobacterium carotovorum*	01001	
	*X. campestris*	01002	
	*C. michiganensis*	VIZR	Dr. Antonova I.A., FSBSI VIZR, deposited in State Collection of Microorganisms, FSBSI VIZR
	*P. syringae*	pv. *maculicola* and *tomato*	
Fungi	*Fusarium oxysporum*	01187	RCAM, ARRIAM
	*Alternaria solani*	03318	
	*Fusarium culmorum*	58800	Dr. T. Yu. Gagkaeva, FSBSI VIZR
	*F. oxysporum*	60521	
	*Fusarium solani*	46011	
	*A. solani*	46011	
	*Bipolaris sorokiniana*		
	*Botrytis* sp.		Mrs. A.V.Erofeeva, ARRIAM

* ARRIAM—All-Russia Research Institute for Agricultural Microbiology (Saint Petersburg, Russia); RCAM—Russian Collection of Agricultural Microorganisms; FSBSI VIZR—All-Russia Institute of Plant Protection (Saint Petersburg, Russia).

**Table 2:** Screening of antagonistic activity of strain b12.3 against bacterial pathogens of plants.

Species	Strain/Pathovar	Activity Intensity *
*P. syringae*	03278	-
	00904	-
	pv. *maculicola*	-
	pv. *tomato*	-
*P. atrosepticum*	01726	Lack of growth
	5128	9 ± 1.1 mm
	01000	-
*C. michiganensis*	VIZR	-
	5351	6 ± 0.75 mm
*X. campestris*	01002	Lack of growth
	23	5 ± 0.67 mm
*P. carotovorum*	01001	-
*Pseudomonas* sp.	8949	-

* Average length of pathogen growth inhibition zone among 10 replicates.

**Table 3:** Screening of antagonistic activity of strain b12.3 against fungal pathogens of plants.

Species	Strain	Activity Intensity *
*F. oxysporum*	01187	-
	60521	-
*F. solani*	46011	-
*F. culmorum*	58800	-
*A. solani*	46011	+
	03318	-
*B. sorokiniana*		-
*Botrytis* sp.		-

* The plus sign denotes the presence of activity, while a dash indicates the absence of activity. The strength of inhibition against *A. solani* was remarkably high since the inhibition zone exceeded 1 cm.

**Table 4:** BGCs found within the b12.3 strain’s genome. Listed are the coordinates of the cluster, the names of the most similar known clusters as determined by antiSMASH v7.0 [66], and the suspected chemical type. Putative biological activity was predicted with the DeepBGC v0.1.30 [67] software. The “-” symbol marks whether the feature is unknown and/or not applicable.

Contig	Genomic Coordinate (b.p.) *	Known Substance	Identity	Chemical Class	Predicted Activity
1	12,670–64,422 (51,752)	Bacillibactin	85.71%	NRPS	-
1	230,027–255,265 (25,238)	Fengycin	40%	Betalactone	-
1	275,519–322,529 (47,010)	-	-	NRPS	-
1	345,867–356,142 (10,275)	-	-	RiPP-like	-
2	187,808–211,342 (23,534)	-	-	LAP	Bactericidal
9	1741–87,617 (85,876)	Butyrolactol A	20%	Polyketide	-
15	4606–48,187 (43,581)	-	-	NRPS-like	-
16	6269–19,986 (13,717)	Petrobactin	100%	Siderophore	-
16	57,070–112,887 (55,817)	Paenilamicin	14.29%	NRP + Polyketide	Bactericidal
18	53,670–63,993 (10,323)	Cerecyclin	30%	RiPP	-
19	70,740–92,593 (21,853)	-	-	Terpene	-
26	22,545–46,459 (23,914)	Paeninodin	30%	RiPP	-
29	42–65,910 (65,868)	-	-	NRPS	-

* The coordinates are given relative to the lengths of the contigs. Cumulative lengths are provided in parentheses.

**Table 5:** A list of insecticidal toxins encoded by loci within the genome of strain b12.3 with the respective predicted host range of the toxins. The specificity data are taken from the BPPRC database [66] for the closest known reference moiety.

Encoded Toxin	Similarity Percent	Affected Host Order	Affected Host Species	References
App4Aa1	68%	Lepidoptera	*Plutella xylostella*	[81]
Tpp78Ba1	75.2%	Hemiptera	*Laodelphax striatellus*	[82]
Spp1Aa1	80.1%	Lepidoptera	*Spodoptera litura*	[83]
		Blattodea	*Blattella germanica*	

## Data Availability

The results of the genome annotation and comparative genomic analysis are provided in the Supplementary Materials of this article. The assembly is available in NCBI databases under accession numbers PRJNA1127832 (BioProject), SAMN42021048 (BioSample), SRR29530170 (SRA), and GCF_040567675.1 (Genome).

## Supplementary Materials

The following supporting information can be downloaded at https://www.mdpi.com/article/10.3390/microorganisms12122450/s1, Data S1: A GBK-formatted result of genome annotation with Prokka v1.14.6; Table S1: Forward and reverse primers used for the *gyrB* gene sequencing; Table S2: The list of biosynthetic gene clusters detected by antiSMASH v7.0 software; Table S3: Biosynthetic gene clusters found in the genome of the strain b12.3 using the DeepBGC v0.1.30 utility; Table S4: The closest 50 reference genomes from the RefSeq database with respective ANI values relative to the genome of the strain b12.3; Table S5: The metadata of the closest reference strains deposited in the RefSeq database; Table S6: Main genomic features (toxins and biosynthetic gene clusters) in the analyzed dataset (strain b12.3 with the 50 closest reference genomes according to ANI estimates); Table S7: Feature-wise characteristics of genomes in the context of predicted toxins, BGCs, and biological activities; Table S8: The abundance of loci encoding virulence factors; Table S9: The cluster-wise total amount of biosynthetic gene clusters in the genomes within the studied dataset; Table S10: The existing information on the 50 closest (relative to the strain b12.3) *B. mycoides* isolates selected according to ANI values; Table S11: Structured information from articles in the NCBI PubMed database related to *B. mycoides* isolates; Table S12: Short summaries from studies related to the microbiota of Lake Baikal and its surrounding territories; Table S13: The spectrum of bactericidal and/or fungicidal activities of strains from the *Bacillaceae* family; Table S14: The list of patented biopreparations using *B. mycoides* strains and patented biopesticides against species inhibited by the strain b12.3 described in the current study; Table S15: Available commercial formulations based on active beneficial microorganisms. Refs. [111,112,113,114,115,116,117,118,119,120,121,122,123,124,125,126,127,128,129,130,131,132,133,134,135,136,137,138,139,140,141,142,143,144,145,146,147,148,149,150,151,152,153,154,155,156,157,158,159,160,161,162,163,164,165,166,167,168,169,170,171,172,173,174,175,176,177,178,179,180,181,182,183,184,185,186,187,188,189,190,191,192,193,194,195,196,197,198,199,248,249,250,251,252,253,254,255,256,257,258,259,260,261,262,263,264,265,266,267,268,269,270,271,272,273,274,275,276,277,278,279,280,281,282,283,284,285,286,287,288,289,290,291,292,293,294,295,296,297,298,299,300,301,302,303,304,305,306,307,308,309,310,311,312,313,314,315,316,317,318,319,320,321,322,323,324,325,326,327,328,329,330,331,332,333,334,335,336,337,338,339,340,341,342,343,344,345,346,347,348,349,350,351,352,353,354,355,356,357,358,359,360,361,362,363,364,365,366,367,368,369,370,371,372,373,374,375,376,377,378,379,380,381,382,383,384,385] can be found in Supplementary Materials.

## Abbreviations

ANI	Average nucleotide identity
BIC	Bayesian information criterion
BPPRC	Bacterial Pesticidal Protein Resource Center
CDS	Coding sequence
HGT	Horizontal gene transfer
LB	Luria–Bertani
MDR	Multidrug-resistant
ML	Maximum likelihood
NRPS	Non-ribosomal peptide synthetases
PDB	Potato dextrose broth
PGPB	Plant growth-promoting bacteria
PKSs	Polyketide synthases
RCAM	Russian Collection of Agricultural Microorganisms
RiPPs	Ribosomally synthesized and post-translationally modified peptides
SNPs	Single-nucleotide polymorphisms
