# Supplementary material for: Genomic Insights into the Bactericidal and Fungicidal Potential of Bacillus mycoides b12.3 Isolated in the Soil of Olkhon Island in Lake Baikal, Russia
Source: Microorganisms. 2024 Nov 28;12(12):2450. doi: 10.3390/microorganisms12122450 (PMC11676374; doi:10.3390/microorganisms12122450)
Supplement: Supplementary file 1 [file microorganisms-12-02450-s001.zip › Supplementary_tables_AS.pdf]

Supporting Information for

Genomic insights into the bactericidal and fungicidal potential of *Bacillus mycoides* b12.3 isolated in the soil of Olkhon Island in Lake Baikal

Maria N. Romanenko[1,2], Anton E. Shikov[1,2], Anton A. Nizhnikov[1,2], Iuliia A. Savina[1], Fedor M. Shmatov[1], Kirill S. Antonets[1,2,*]

[1]Laboratory for Proteomics of Supra-Organismal Systems, All-Russia Research Institute for Agricultural Microbiology (ARRIAM), 196608 St. Petersburg, Russia;
[2]Faculty of Biology, St. Petersburg State University (SPbSU), 199034 St. Petersburg, Russia.
*** Correspondence: Kirill S. Antonets,** k.antonets@arriam.ru

This file includes:
Tables S11-S15

Descriptions of Tables S1 to S15

Supporting Tables

Table S11. The structured information from articles in the NCBI Pubmed database related to *B. mycoides* isolates in the context of produced metabolites and their activities (searching term – *Bacillus mycoides*). Lusted are the tested pathogens (bacteria/fungi) and BGCs presented in the strains' genomes and/or identified by chromatography methods. The presence/absence of the activity is marked with "+" and "-" symbols, respectively. In case the feature was not reported, the cell contains "NT". The green background highlights BGCs/pathogens that were studied in the current work.

Strain	BGCs	Pathogen	Pathogen type	Activity	References
Bacillus mycoides A1	NT	*Colletotrichum gloeosporioides*	Fungi	+	[114]
Bacillus mycoides ATCC 21929 (patented strain) -> *Bacillus clarus*	Paeninodin, Bacillibactin, Fengycin, Iturin, Puwainaphycin, antibiotic 60-6 (cerexin A – not found), using whole genome analysis	*Bacillus subtilis* PCI 29	Bacteria	+	[159,160]
		Bacillus anthracis			
		Staphylococcus aureus 209 P			
		Streptococcus pyogenes C-203			
		Diplococcus pneumoniae type I			
Bacillus mycoides strain BM103	NT	"*Candidatus phytoplasma*"	Fungi	+	[161]
Bacillus mycoides	NT	*Escherichia coli*	Bacteria	-	[162]
		Staphylococcus aureus			
		Pseudomonas aeruginosa			
		Klebsiella pneumoniae			
		Acinetobacter baumanii			
Bacillus mycoides BM02	No BGCs, only Phenylacetic acid, and methyl phenyl acetate volatile compounds	*Fusarium oxysporum* f. sp. *lycopersici*	Fungi	+	[25]
Bacillus mycoides isolate J (*Bm*J), commercial	NT	*Erysiphe necator*	Fungi	+	[163]
Bacillus mycoides isolate MF377573	NT	*Phytophthora cinnamomi*	Fungi	-	[91]
		Graphium euwallaceae			
		Graphium sp.			
		Fusarium euwallaceae		+	
Bacillus mycoides isolate MF377556		*Erysiphe necator*		-	
		Phytophthora cinnamomi			
		Graphium euwallaceae			

		Graphium sp.			
		Fusarium euwallaceae		+	
Bacillus mycoides 4079	Surfactins, bacillomycin D, iturin A, fengycin (using MALDI-TOF and PCR)	*Sclerotinia sclerotiorum*	Fungi	+	[164]
Bacillus mycoides S	Zwittermicin A (with PCR)	*Sclerotinia sclerotiorum*	Fungi	+	[115]
Bacillus mycoides isolate J (*Bm*J), commercial	NT	*Erwinia amylovora*	Bacteria	+ (worse than other biopesticides)	[165]
Bacillus mycoides ATCC 21929 (patented strain) -> *Bacillus clarus*	Cerexin A1 (using HPLC and MS/MS)	*Staphylococcus aureus* ATCC 6538	Bacteria	+	[113]
Bacillus mycoides A10	NT	*Vibrio mimicus*	Bacteria	+	[166]
Bacillus mycoides 50-12	NT	*Helminthosporium solani*	Fungi	+	[167]
Bacillus mycoides strain SU-23	NT	*Pythium mamillatum*	Fungi	+	[22]
B. mycoides K92/2	NT	*Alternaria helianthi*	Fungi	+ (no effect on infection after 24 h inoculation with the pathogen)	[168]
B. mycoides isolate	NT	*Fusarium oxysporum*	Fungi	+	[92]
Bacillus mycoides strain R2	Styrene (identified by Gas chromatography)	*Meloidogyne incognita*	Nematode	+	[169]
		Caenorhabditis elegans			
Bacillus mycoides isolate J (*Bm*J), commercial	NT	*Glomerella cingulata* var. *orbiculare*	Fungi	+	[21]
Bacillus mycoides B23	NT	*Alternaria alternata*	Fungi	+	[170]
Bacillus mycoides PR04	NT	*Pseudomonas aeruginosa*	Bacteria	-	[88]
		Salmonella typhimurium			
		Staphylococcus aureus		+	

		Listeria monocytogenes			
		Candida albicans	Fungi	+	
Bacillus mycoides 7-B7	NT	*Aspergillus carbonarius*	Fungi	+	[171]
		Aspergillus niger			
		Aspergillus flavus			
Bacillus mycoides DFC 1	Polyhydroxybutyrate (no genome sequence, only produced vanillin-incorporated PHB films were tested)	*Staphylococcus aureus* MTCC 737	Bacteria	+	[89]
		Salmonella typhimurium MTCC 98			
		Escherichia coli MTCC 23058			
		Shigella flexneri MTCC 1457			
		Aspergillus flavus MTCC 277	Fungi	+	
		Aspergillus fumigatus MTCC 15066			
		Aspergillus niger MTCC 478			
		Aspergillus parasiticus MTCC 8189			
		Aspergillus ochraceus MTCC 1877			
		Penicillium viridicatum MTCC 4973			
		Penicillium clavigerum MTCC 9182			
Bacillus mycoides HF-1	NT	*Alternaria ricini*	Fungi	+	[172]
Bacillus mycoides CA1	NT	*Sporisorium scitamineum*	Fungi	+	[173]
		Ceratocystis paradoxa			
Bacillus mycoides (putative)	NT	*Listeria monocytogenes*	Bacteria	+	[174]
Bacillus mycoides JC192	NT	*Gaeumannomyces graminis* var. *tritici*	Fungi	+	[175]
Bacillus mycoides K184					
Bacillus mycoides SelTE01	Selenium nanoparticles produced by the bacterium	*Pseudomonas aeruginosa* NCTC 12934	Bacteria	+ (NPs tested solely)	[176]
		Staphylococcus aureus ATCC 25923			

Bacillus mycoides B16	NT	*Botrytis cinerea*	Fungi	+	[93]
Bacillus mycoides A10	NT	*Vibrio mimicus*	Bacteria	+	[177]
Bacillus mycoides B16	NT	*Botrytis cinerea*	Fungi	+	[178]
Bacillus mycoides isolate J (*Bm*J), commercial	NT	*Cercospora beticola*	Fungi	+	[111]
Bacillus mycoides isolate J (*Bm*J), commercial	NT	*Cercospora beticola*	Fungi	+	[112]
Bacillus mycoides QAUBM19	NT	*Escherichia coli* ATCC 25922	Bacteria	+	[90]
		Streptococcus pneumoniae ATCC 49619			
		Pseudomonas aeruginosa ATCC 27853		-	
		Salmonella enterica ATCC 27870			
		Staphylococcus aureus ATCC 6538			
B. mycoides QAUBM1901		*Escherichia coli* ATCC 25922		+	
		Streptococcus pneumoniae ATCC 49619		-	
		Pseudomonas aeruginosa ATCC 27853			
		Salmonella enterica ATCC 27870			
		Staphylococcus aureus ATCC 6538			
Bacillus mycoides B16	NT	*Botrytis cinerea*	Fungi	+	[94]
Bacillus mycoides BM02	Biosurfactants (putatively kurstakins, surfactins, and iturins)	*Pythium aphanidermatum*	Fungi	+	[23]
Bacillus mycoides NP02					
Bacillus mycoides CHR003				-	
Bacillus mycoides WT15					

Table S12. Short summaries from studies related to the microbiota of Lake Baikal with surrounding territories. Listed are isolation sites of the bacteria, taxonomic assignments of selected microorganisms, and the types of research. The green color highlights the same categories as those conducted in this research whereas the yellow color represents partial similarity, i.e., isolation from sediments (but not soil) and examining *Bacillus* sp. of unknown species.

Location	Organism	Research Type	Reference

Lake Baikal, bottom ice surface and in the water column under the ice	Proteobacteria (*Burkholderiaceae*), Actinobacteria (*Sporichthyaceae* and *Ilumatobacteraceae*) and Verrucomicrobia (*Methylacidiphilaceae*), Bacteroidetes (*Flavobacteriaceae*), Verrucomicrobia (FukuN18_freshwater_group, *Methylacidiphilaceae*), and cyanobacteria (Cyanobiaceae)	Comparison of particle-associated and free-living organisms	[4]
Freshwater endemic alga *Draparnaldioides baicalensis* near the village Bolshie Koty	*Streptomyces*, *Saccharopolyspora*, *Nonomuraea*, *Rhodococcus*, and *Micromonospora*	Taxonomic classification and assessing the bactericidal activity of the isolated strains	[5]
The bottom sediments of Lake Baikal, sampled in the coastal zone (Slyudyanka, Listvyanka, Kultuk)	*Pseudomonas* spp., *Bacillus megaterium*, *Micrococcus*, and *Acinetobacter*	Antibacterial activity screening against human pathogens, isolation of antimicrobial peptides (undefined)	[6]
Epilithic biofilms from the littoral zone of Lake Baikal near the settlement of Listvyanka	*Bacillus*, *Paenibacillus*, *Pseudomonas*, *Aeromonas*, *Serratia*, *Rhizobium*, *Devosia*, *Yersinia*, *Iodobacter*, *Kocuria*, *Pseudoclavibacter*, *Microbacterium*, *Brachybacterium*, and *Flavobacterium*	Mining for bactericidal genes (PKS and NRPS) using PCR and testing enzyme activity	[7]
Oil seeps near the Gorevoy Utes, and near the mud volcano Bolshoy	*Cyanobacteria*, *Alphaproteobacteria*, and *Gammaproteobacteria*	Studying taxonomic distribution and phylogenetic relationships	[8]
Sub-ice samples at the station of the ice camp, which was located 7 km from the Listvyanka settlement in the water column	Verrucomicrobiota, Actinobacteriota, Bacteroidota, and Cyanobacteria	Characterizing dynamics in alterations of taxonomic composition, and characterizing bacteriophages	[9]
Gastrointestinal tract of Baikal phytophagous amphipods	*Streptomyces* sp., *Rhodococcus* sp., *Microbacterium* sp.	Antibacterial activity screening against type strains, estimation of natural products using LC-MS	[10]
Water columns in the central station of the Ukhan–Tonkii transect (the central Baikal basin)	Proteobacteria, Actinobacteria, Chloroflexi, Bacteroidetes, Firmicutes, Acidobacteria and Cyanobacteria	Comparison of taxonomic composition	[11]
Subglacial sample taken at Lake Baikal near Bolshie Koty settlement	*B. mycoides* BS2-15	Evaluation of algicidal activity	[13]

Water and bottom sediments of the Selenga River and Lake Baikal	*Bacillus*, *Pseudomonas*	Characterization of degradation of bis(2-ethylhexyl)phthalat	[179]
Sub-ice samples at the station of the ice camp, which was located 7 km from the Listvyanka settlement in the water column	*Verrucomicrobia*, *Actinobacteria*, and *Proteobacteria*	Characterizing microbial communities, genome assemblage from metagenomic data, and phylogenetic analysis	[180]
Kotelnikovsky Hot Springs (Lake Baikal)	*Bacillus* spp.	Morphological and biochemical analysis of the isolated strains	[181]
Lake Baikal sediments (unspecified)	*Geobacillus*, *Bacillus* spp (including *B. mycoides*), *Clostridium*	Analyzing the diversity and phylogeny based on the *spoA* gene	[14]
Lake Baikal sediments (unspecified)	*B. mycoides*	Testing PCR efficiency using magnetic polymer microspheres	[15]
Bottom sediments collected at the places of natural oil seeps at Lake Baikal close to the Zelenovskaya River mouth	*Pseudomonas*, *Bacillus*, *Rhodococcus*, and *Micrococcus*	Assessing the ability of the isolated strains to metabolize alkanes	[182]
Baikal sponges in the coastal waters of Lake Baikal	*Pseudomonas*, *Bacillus*, *Micrococcus*, *Sarcina*, *Flavobacterium*, *Arthrobacter*, *Acinetobacter*, *Streptomyces*, and *Micromonospora*	Identifying taxonomic diversity of sponge microbiome	[183]
Low-temperature surface sediments near the Posolsk Bank methane seep, Lake Baikal	*Thermaerobacter* PB12/4term	Phenotypical and biochemical characterization of the novel strain, whole genome sequencing, and annotation	[184]
Surface coastal water of Southern Baikal	Actinobacteria, Proteobacteria, and Bacteroidetes	Characterizing bacterial and viral composition	[185]
The bathypelagic water column of Lake Baikal at 1600 m	*Candidatus* Patescibacteria	Genome assembly from metagenomic reads, phylogenetic survey of the candidate bacterial group	[186]
Hot spring near Goryachinsk in Baikal Lake region	*Fontisphaera persica* gen. nov. strain B-154	Phenotypical characterization of the novel strain, whole genome sequencing, annotation, and phylogenetic analysis	[187]
South Baikal from the Central Basin	*Enterobacter* and *Pseudomonas*	Morphological description of the strains and revealing factors determining motility	[188]
The underwater part of the wooden pier located in the area of the Scientific Research Station “Bolshiye Koty” of the Limnological	*Tychonema* sp. BBK16	Phenotypical characterization of the novel strain, whole genome sequencing, annotation, phylogenetic analysis, and examination of prophages	[189]

Institute (Bolshiye Koty Settlement)			
Diseased *L. baicalensis* sponge collected from Lake Baikal	*Janthinobacterium* sp. SLB01	Morphological and physiological description of the novel strain	[190]
Upper sediment layer in Southern Baikal	Proteobacteria, Actinobacteria, Acidobacteria, Cyanobacteria, Bacteroidetes, Firmicutes (including *Bacillus* sp.)	Populational analysis of bacterial communities	[191]
Mollusks *Benedictia baicalensis* from Lake Baikal near Bolshiye Koty village	*Streptomyces* sp. IB201691-2A	Screening for antibacterial activity of novel angucyclines Baikalomycins A-C	[102]
Diseased *L. baicalensis* sponge collected from Lake Baikal	*Janthinobacterium* sp. SLB01	Morphological description, genome sequencing, and annotation	[192]
Mollusks *Eulimnogammarus verrucosus* collected in the littoral zone of Lake Baikal	*Pseudomonas* sp	Isolation, taxonomic identification, and cultivation of strains to test the immune response of the hosts	[193]
Sponges *L. baicalensis* from the southern, central, and northern Baikal basins	Verrucomicrobia, Cyanobacteria, Bacteroidetes, and Proteobacteria	Taxonomic classification of sponge metagenome	[194]
Water and sediment in Central Baikal at the 'Saint Petersburg' hydrate site	Methanomicrobiales Methanosarcinales, Chloroflexi, candidate division JS1, and Caldiserica.	Taxonomic classification of bacteria residing in hydrate sites	[195]
Water samples were taken in 30 stations of Lake Baikal at different depths	Actinobacteria, Bacteroidetes, Verrucomicrobia, Proteobacteria, Acidobacteria, and Cyanobacteria	Studying microbial community in the Photic Layer of Lake Baikal's Three Basins	[196]
Hot spring near Goryachinsk town in Baikal lake region	*Fontivita pretiosa gen*. nov., sp. nov.	Morphological and biochemical characterization of the novel strain, genome sequencing with annotation	[197]
Hot spring in Zmeinaya Bay in the Baikal lake region	*Thiothrix* sp.	Morphological and biochemical characterization of the novel species, genome sequencing with annotation	[198]
Water and bottom sediments in Oil seepage near Bol'shaya Zelenovskaya River mouth	Unknown	Assessing the oil-oxidizing activity of resiging microorganisms	[199]
Sediment samples from varying depths in the	Unknown	Revealing components of the methane cycle	[200]

northern, central, and southern basins of Lake Baikal			
Soil of solonchaks and silts of Western Transbaikalia	Unknown	Biochemical characterization of the microflora	[110]
Sponge *Baikalospongia intermedia* near the Ukhan Cape and to the south at Tolsty Cape	Proteobacteria, Actinobacteria, Planctomycetes, Cloroflexi, Verrucomicrobia, Acidobacteria, Chlorobi, and Nitrospirae	Revealing taxonomic distribution of sponge microbiome	[201]
Deepwater amphipod species at different depths from the southern part of Lake Baikal near the Bolshie Koty settlement	*Streptomyces*, *Micromonospora*, and *Pseudonocardia*	Isolation and taxonomic classification of Actinobacteria, characterizing antimicrobial compounds with LC-MS and testing bactericidal and fungicidal activities	[104]
Male cones of Scots pine trees (*Pinus sylvestris*) growing on the shore of Lake Baikal	*Streptomyces*, *Rhodococcus*, *Amycolatopsis*, and *Micromonospora*	Isolation and taxonomic classification of Actinobacteria, characterizing antimicrobial compounds with LC-MS and testing bactericidal and fungicidal activities	[105]
The water surface of underground lakes from Badzheyskaya and Okhotnichya caves in Lake Baikal region	*Streptomyces*, *Nocardia*, and *Nocardiopsis*	Isolation and taxonomic classification of Actinobacteria, evaluating the composition of fatty acids, and testing bactericidal and fungicidal activities	[202]
Bottom sediments, and sponges of Lake Baikal	*Streptomyces* and *Micromonospora*	Testing bactericidal and fungicidal activities	[203]
Bottom sediments in oil seeps near Cape Gorevoi Utes	Alphaproteobacteria	Taxonomic classification of microbial community	[204]
Diseased *L. baicalensis* sponge collected from Lake Baikal	*Janthinobacterium* sp. SLB01 and *Flavobacterium* sp. SLB02	Genome assembly with annotation, phylogenetics, and comparative genomics of the strains	[205]
Benthic invertebrates near Listvyanka village in South Baikal	*Streptomyces* sp. IB2014/011-12	Genome assembly with annotation, studying biochemical pathway of bioactive alpiniamides, and testing bactericidal activity	[103]
Benthic invertebrates near Listvyanka village in South Baikal	*Streptomyces*, *Nocardia*, *Pseudonocardia*, *Micromonospora*, *Aeromicrobium*, and *Agromyces*	Taxonomic classification of microbial community, testing bactericidal and fungicidal activities	[106]
Water samples taken near the shore (near Berezovyi) and in the depths (the central point	*Streptomyces* sp. 156A	Testing bactericidal activity of secondary metabolites (polynacin)	[206]

of Listvyanka and Tankhoi) of the lake			
Water samples taken near the shore (near Berezovyi) and in the depths (the central point of Listvyanka and Tankhoi) of the lake	Actinomycetes, mostly *Streptomyces* sp.	Testing the bactericidal activity of the isolated strains	[207]
Alkaline hot spring of the Baikal Lake area.	*Thermincola carboxydiphila* gen. nov., sp. nov.	Morphological, biochemical, and phylogenetic characterization of the novel strain	[208]
Verkhnee Beloe soda lake in Lake Baikal region	*Clostridium alkalicellum* sp. nov.	Morphological, biochemical, and phylogenetic characterization of the novel strain	[209]
L. baicalensis sponge collected from Lake Baikal	Actinobacteria, Proteobacteria, Verrucomicrobia, Bacteroidetes, Cyanobacteria, and Nitrospira	Characterizing taxonomic diversity	[210]
Legume *Oxytropis triphylla* originating from Zunduk Cape in the Baikal Lake region	*Phyllobacterium zundukense* sp. nov	Taxonomic classification and biochemical characterization of the novel species	[211]
The bottom sediments at Posol'skaya Shoal station	*Nitrospirae*, *Pseudomonas*	Taxonomic classification of microbial community	[101]
Plankton and biofilm samples in the region of the Bol'shie Koty settlement	*Cyanobacteria*, *Bacteroidetes*, and *Proteobacteria;*	Taxonomic classification of microbial community	[212]
Astragalus chorinensis and *Oxytropis popoviana* originated from Buryatia in the Baikal Lake region	*Mesorhizobium japonicum* and *M. kowhaii*	Taxonomic identification of strains and analyzing nodulation after inoculation	[213]
Bottom sediments of Lake Baikal in the Shoal Posol'skaya	*Bacillus* (*B. cereus*, *B. subtilis*, *B. coagulans*), *Pseudomonas*, *Acinetobacter*, and *Micrococcus*	Taxonomic identification and morphological description of the strains	[107]
Surface microlayer in the southern basin of Lake Baikal	Alphaproteobacteria and Verrucomicrobi	Taxonomic classification of microbial community	[100]
Bitumen mound at the natural oil seep near Cape of Gorevoi Utes in Central Baikal	*Rhodococcus erythropolis* strain 4	Genome assembly with annotation of the novel strain	[214]
Sponge *Swartschewskia papyracea* near the Bol'shie Koty settlement	Verrucomicrobia, Cyanobacteria, and Proteobacteria	Taxonomic classification and revealing PKS genes in sponge microbiome	[215]

Plankton samples in the upper water layer layer of the South Baikal	*Trichormus variabilis*	Morphological description and taxonomic classification of the collected strains	[216]
Pelagic zone of the lake from stations in Barguzin Bay. Chivurkuy Bay, and Maloe More Strait	Actinobacteria, Bacteroidetes, and Alphaproteobacteria	Revealing the correlation between environmental factors and microbial composition	[217]
Surface microlayer, water column, bottom water, and epilithic biofilms in the littoral zone of the southern basin of Lake Baikal off the Bol'shiye Koty settlement	Rhodobacterales, Rhizobiales, Rhodospirillales, Sphingomonadales, Burkholderiales, Gemmatimonadetes, and Planctomycetes	Taxonomic classification of microbial community	[218]
Upper water columns throughout Lake Baikal	Unknown	Correspondence between environmental factors and physical characteristics of bacterial colonies	[219]
Sediment samples surrounding the Kedr-1 mud volcano in Lake Baikal	Bathyarchaeia, Caldatribacteriota, and Chloroflexota, methanogens *Methanoregulaceae*, *Methanosaetaceae*, and Methanomassiliicoccales	Correspondence between environmental factors and physical characteristics of bacterial colonies	[220]
Water columns from the underside of the ice sheet in Southern Baikal near the settlement of Bolshie Koty	Proteobacteria, Verrucomicrobia, Actinobacteria, Acidobacteria, Bacteroidetes, and Cyanobacteria	Taxonomic classification of microbial community	[221]
Phytophagous amphipods *Eulimnogammarus cyaneus* from the southern part of Lake Baikal	*Streptomyces* sp.	Morphological, biochemical, and phylogenetic characterization of the novel bacteria, metabolic profiling with LC-MS, and bactericidal activity testing	[222]
Pelagic zone of the lake from stations in Barguzin Bay. Chivurkuy Bay, and Maloe More Strait	Actinobacteria, Bacteroidetes, and Verrucomicrobia	Revealing the correlation between environmental factors and microbial composition	[95]
Natural oil seepage on Lake Baikal	*Rhodococcus erythropolis* and *Pseudomonas fluorescens*	Measuring hydrocarbon-oxidizing when adding protatranes	[96]
Benthic invertebrates in Central and North Baikal	Bacteroidetes and Firmicutes	Taxonomic classification of microbial community	[223]
The central basin of Lake Baikal: in the pelagic area in the	*Anabaena* and *Microcystis*	Taxonomic classification of phytoplankton	[224]

middle of the Ukhan–Tonkii section			
The water surface microlayer in Central and South Baikal	*Deinococcus ficus* strain NA202, Actinobacteria, Bacteroidetes, Proteobacteria, Firmicutes	Morphological, physiological, and biochemical characterization of heterotrophic and taxonomic assessment of the community	[225]
Baikalospongia bacillifera sponges in the southern basin of Lake Baikal, near Bolshiye Koty	Bacteroidetes, Proteobacteria Actinobacteria, Firmicutes	Comparing the microbiome and virome of marine and freshwater sponges	[226]
The littoral zone of Southern Baikal near the settlements of Listvyanka and Bolshiye Koty	*Tolypothrix distorta*	Assessing the production of toxins, taxonomic description of novel strains	[227]
Bottom sediments from the Lake Baikal area of the methane seep Posolskaya Bank	*Sphingomonas*, *Solirubrobacter*, and *Arthrobacter*	Taxonomic and morphological classification of the isolates, examining metabolic capacities under thermobaric conditions	[228]
Ice cores from in-shore and offshore sites of Southern Lake Baikal	*Synechococcus* and *Cyanobium*	Describing taxonomic composition and environmental characteristics of the samples, morphological description of strains	[229]
Water samples throughout the whole area of Lake Baikal	*Caulobacter* and *Brevundimonas*	Morphological, physiological, and biochemical analysis of strains and taxonomic identification of the isolates	[99]
Bottom sediments from Lake Baikal deep and coastal parts of the Frolikha Bay, in the Selenga estuary, and in the Barguzin Bay	*Thioploca*	Physicochemical characterization of sites, Morphological and phylogenetic analyses of bacteria	[230]
Biofilms on rocks in the southern basin of Lake Baikal near Cape Berezovy	*Rivularia rufescens*, *Tolypothrix limbata*, *Chamaesiphon fuscus*, *Ch. subglobosus*, and *Heteroleibleinia pusilla*.	Morphological and taxonomic description of the microbiome	[231]
Freshwater sponges *Lubomirskia Baikalensis* in the Olkhon Gate Strait	Bacteroidetes, Proteobacteria, *Flavobacteriaceae*, *Burkholderiaceae*, and *Moraxellaceae*.	Morphological and taxonomic description of the microbiome	[232]
Health and diseased sponges *Lubomirskia Baikalensis* along the littoral zone of Lake Baikal	*Oscillatoriaceae*, *Cytophagaceae*, *Flavobacteriaceae*, *Chitinophagaceae*, *Sphingobacteriaceae*, *Burkholderiaceae*, *Rhodobacteraceae*, *Comamonadaceae*,	Comparing the taxonomic composition of microbiomes from healthy and diseased sponges	[233]

	Oxalobacteraceae, and *Xanthomonadaceae*		
Water samples in large polluted and unpolluted rivers in the Baikal region	Unknown	Analyzing the impact of water pollution on bacterial production	[234]
Central and southern Baikal at the sites of discharge of hydrocarbon-containing fluids	Undefined methanotrophic bacteria	Measuring activities of methanotrophic bacteria from different water columns	[235]
Alkaline brackish Lake Solenoe in the Transbaikalian region	*Belliella buryatensis* sp. nov.	Biochemical and taxonomic classification of the novel species	[236]
Water columns at the central station on the Listvyanka settlement–Tankhoi settlement	Proteobacteria, Flavobacteria	Assessing microbial composition using the *in situ* Hybridization Method	[237]
Bottom sediments at the Posol'skaya Banka elevation	*Desulfosporosinus*, Clostridiales	Taxonomic identification and assessing the rate of sulfate reduction	[238]
Microbial mats of the Goryachinsk thermal spring in Lake Baikal	*Chloroflexus aurantiacus*, *Blastochloris sulfoviridis*, *Rhodomicrobium vannielii*, *Allochromatium* sp, and *Thiocapsa* sp.	Morphological description and taxonomic classification of anoxygenic phototrophic bacteria	[239]
Baikal water (unspecified)	Unknown	Studying the process of bacterial aggregation when adding glucose	[240]
Sediments from the Sukhaya thermal spring in Lake Baikal	*Methylocystis* and *Methylosinus*	Taxonomic identification and physiological characterization	[241]
Benthic sediments of Lake Alginskoe, a soda lake located in the Trans-Baikal Region	*Desulfonatronum zhilinae* sp. nov.	Morphological description, taxonomic classification, and biochemical testing of the novel species	[242]
Soda lake Nizhnee Beloye, south-east of Baikal	*Anoxynatronum sibiricum* gen.nov., sp.nov.	Morphological description, taxonomic classification, and biochemical testing of the novel species	[243]
Irkutsk Reservoir formed by the dam of the Irkutsk hydroelectric power station	*Dolichospermum lemmermannii*	Morphological description, taxonomic classification, and quantification of Saxitoxin	[244]
Bottom sediment in Lake Baikal in the region of the Akademicheskii Ridge	*Leptothrix*, *Siderocapsa*, *Naumaniella*, and *Bacillus*	Morphological description and physiological study of bacteria	[108]
Endemic sponges *Baicalospongia* sp. and *Lubomirskia baicalensis* in Lake Baikal near Listvyanka	Actinobacteria, Alphaproteobacteria, Betaproteobacteria, Firmicutes, and Gammaproteobacteria	Taxonomic classification of the microbiome and evaluating the efficacy of the novel cultivation method, I-tip	[245]

Bottom sediments from Lake Baikal	*Variovorax* sp.	Morphological description, taxonomic classification, and biochemical testing of bacteria from *Thioploca* Mats	[246]
Water in the Southern part of Lake Baikal	Cyanobacteria, Proteobacteria, and Actinobacteria	Taxonomic profiling of the samples	[247]
Oil seep in Mid-Baikal (Cape Gorevoi Utes)	Unknown	Measuring hydrocarbon-oxidizing activity of microbial communities	[248]
Lubomirskia baicalensis and *Baikalospongia* sp. sponges in the neighborhood of the Bolshie Koty settlement	Bacteroidetes, Proteobacteria, and Actinobacteria	Taxonomic profiling of the metagenome of sponges	[249]
Terrestrial hot springs in the Baikal Lake region	*Tepidisphaera mucosa* gen. nov., sp. nov	Morphological description, taxonomic classification, and biochemical testing of the novel species	[250]
Posolski Bank Methane Seep, Southern Baikal	*Thioploca ingrica*, Bacteroidetes, Nitrospira, Chloroflexi, Planctomycetes, Verrucomicrobia, Proteobacteria, Euryarchaeota, Crenarchaeota, and Thaumarchaeota	Morphological description of *Thioploca* bacteria forming mats, taxonomic classification of the bacterial community	[251]
Sediments of the Gorevoy Utes natural oil seep	Firmicutes, Chloroflexi, and Caldiserica (OP5), Deltaproteobacteria and Methanomicrobi	Analyzing cultural characteristics, revealing the taxonomic distribution of the metagenome, and examining anaerobic oxidation of petroleum hydrocarbons	[252]
Lake Baikal water (unspecified)	*Phormidium uncinatum*	Assessing biochemical characteristics at different salt concentrations	[253]
Gastropods from Lake Baikal in the zone of underwater hydrothermal vents of the Frolikha Bay	*Cristispira*	Morphological and biochemical description of the gastropods' microbiome	[254]
Water in the zone of the Selenga inflow into Lake Baikal	Gammaproteobacteria and Betaproteobacteria	Taxonomic classification of planktonic bacteria in different zones	[255]
Sediments in the region of subsurface discharge of methane hydrates in the southern part of Baikal	*Pseudomonas* sp.	Taxonomic classification of the microbiome	[256]
Deep pelagic waters of Lake Baikal	Unknown	Studying the effect of extreme temperatures on cultural growth	[257]

B. intermedia sponges in the Mid-Baikal area	Cyanobacteria	Taxonomic classification and studying the diversity of the *psbA* gene in the microbiome of sponges	[258]
Lake Baikal water at the central stations of three transects	Cyanobacteria and Proteobacteria	Morphological description and taxonomic classification of the microbiome	[259]
Bottom sediment in hydrothermal vents in the central basin	*Methylocystis*	Morphological description and taxonomic classification of the microbiome, and testing methane consumption intensity	[260]
Water in the coastal zone of the Barguzin and Kurkut Bays (Maloe More Strait)	*Aphanizomenon* and *Anabaena*	Morphological identification and assessment of genes encoding for neurotoxins	[261]
Water in the middle section of Lake Baikal	Unknown	Studying the abundance and physical characteristics of bacterial populations in water columns of various depths	[97]
Water near the Kultuk settlement in Lake Baikal	*Pseudomonas* and *Enterococcus*	Studying the efficacy of calcium hypochlorite on water sterilizing	[262]
Hot springs near Lake Baikal (Barguzin National Park)	*Thermoanaerobacter* and *Caldoanaerobacter*	Morphological and taxonomic characterization of novel strains and studying arginase activity	[98]
Baikalian winter picoplankton	*Synechococcus*, *Microcystis elabens*, *Cyanobacterium* sp., *Prochloorococcus marinus*	Taxonomic characterization of Baikalian picoplankton	[263]
Water samples and sediments embracing the entire water area of Lake Baikal	*Streptomyces*, *Micromonospora*	Describing morphological and biochemical characteristics of actinomycetes in Lake Baikal	[264]
Bottom sediments of low mineralized alkaline hydrothermal vents of Goryachinsk	*Anoxybacillus*	Morphological, taxonomic, and physiological classification of novel alkaliphilic strains	[265]
Water and bottom sediments in two areas of natural oil seepage on Lake Baikal near the Bol'shaya Zelenovskaya River mouth and near Cape Gorevoi Utes	*Pseudomonas*, *Bacillus*, *Brevibacillus*, *Paenibacillus*, *Micromonospora*, *Rhodococcus*, *Sphingomonas*, *Mycobacterium*, *Bosea*, *Micrococcus*, *Microbacterium*, *Arthrobacter*, *Novosphingobium*, *Methylobacterium*, *Curtobacterium*, and *Acidovorax*	Taxonomic characterization of aerobic in oil seepage	[109]
Bottom sediments were sampled from a well drilled in the Baikal rift	*Roseomonas baikalica* sp. nov.	Morphological, taxonomic, and physiological classification of novel species	[266]
Kotel'nikovskii Hot Spring of the Baikal Rift	*Pseudanabaena* sp. 0411 and *Synechococcus* sp. 0431	Morphological, taxonomic, and physiological classification novel strains	[267]

Table S13. The spectrum of bactericidal and/or fungicidal activities of strains from the *Bacillaceae* family. The criteria of inclusion for the research were the presence of at least one BGC and tested phytopathogens described in this study. If the isolate was already pre-tested on a certain pathogen before conducting the research, the respective cell contains "NT". In case the strain was active/non-active against all the pathogens tested, the "-" symbol is used. The entities mentioned in this study (BGCs/pathogens) are highlighted in green.

BGC	Organism	Activity type	Active against	Not active against	Reference
Bacillibactin	*Bacillus amyloliquefaciens* MBI600	Bactericidal	*Pseudomonas syringae*	NT	[121]
		Fungicidal	*Fusarium oxysporum*, *Rhizoctonia solani*, *Aspergillus flavus*, *Verticillium dahliae*	*Phytophthora cactorum*, *Botrytis cinerea*	
Bacillibactin (and Bacillomycin, Fengycin)	*Bacillus amyloliquefaciens* SQR9	Fungicidal	*Sclerotinia sclerotiorum*, *Fusarium oxysporum*, *Rhizoctonia solani*, *Fusarium solani*, *Verticillium dahlia*, *Phytophthora parasitica*	-	[268]
Bacillibactin (and Surfactin, Fengycin, Bacillomycin)	*Bacillus velezensis* FZB42	Bactericidal	*Xanthomonas campestris*	NT	[269]
Bacillibactin (and Bacilysin, Fengycin, Difficidin)	*Bacillus amyloliquefaciens* GKT04	Fungicidal	*Fusarium oxysporum*	NT	[149]
Bacillibactin (and Bacillaene, Fengycin, Bacilysin, Subtilosin, Surfactin)	*Bacillus stercoris* B.PNR1	Fungicidal	*Fusarium oxysporum*, *Sclerotium rolfsii*, *Colletotrichum musae*, *Colletotrichum gloeosporioides*	NT	[270]
Bacillibactin (and Surfactin, Iturin, L-dihydroanticapsin, Oxydifficidin, Azelaic acid)	*Bacillus velezensis* Bvel1	Fungicidal	*Botrytis cinerea*	NT	[271]
Bacillibactin (and Fengycin, Surfactin, Iturin, Bacillaene, Plantazolicin, bacilysin)	*Bacillus thuringiensis* CHGP12	Fungicidal	*Fusarium oxysporum*	NT	[272]
Bacillibactin (and Fengycin, Bacilysin,	*Bacillus* sp. AFE 4A	Fungicidal	*Rhizoctonia solani*, *Fusarium culmorum*, *Gaemannomyces graminis*, *Pythium ultimum*	-	[273]

Lanthipeptide class II, Difficidin, Macrolactin, Bacillaene)					
Bacillibactin (and Fengycin, Bacilysin, Difficidin, Macrolactin, Bacillaene, surfactin)	*Bacillus* sp. AFE 21	Fungicidal	*Rhizoctonia solani*, *Fusarium culmorum*, *Gaemannomyces graminis*,	*Pythium ultimum*	
Bacillibactin (and Surfactin, Fengycin, Iturin, Bacilysin, Bacillaene, and Butirosin)	*Bacillus velezensis* WB	Fungicidal	*Fusarium oxysporum*	NT	[274]
Bacillibactin (and Surfactin, Fengycin, Butirocin, Difficidin, Bacilysin, Mersacidin)	*Bacillus velezensis* VB7	Fungicidal	*Sclerotinia sclerotiorum*, *Fusarium oxysporum*	NT	[275]
Bacillibactin (and Subtilomycin, Bacillaene, Fengycin, Pulcherriminic acid, Subtilosin A, and Bacilysin)	*Bacillus subtilis* GYUN-2311	Fungicidal	*Colletotrichum acutatum*, *Colletotrichum coccodes*, *Colletotrichum siamense*, *Colletotrichum fructicola*, *Colletotrichum gloeosporioides*, *Colletotrichum aenigma*, *Colletotrichum fioriniae*, *Colletotrichum nymphaea*, *Diplodia seriata*, *Botryosphaeria dothidea*, *Fusarium solani*, *Fusarium oxysporum*	-	[276]
Bacillibactin (and Fengycin, Surfactin, Mojavensin, Bacillaene, L-dihydroanticapsin, Bacillibactin	*Bacillus halotolerans* Cal.l.30	Fungicidal	*Botrytis cinerea*	NT	[277]
Bacillibactin (and Rhizoctin, Surfactin, Bacillaene, Fengycin,	*Bacillus cabrialessi* BH5	Fungicidal	*Rhizoctonia solani*, *Botrytis cinerea*, *Verticillium dahliae*, *Phytophthora infestans*	-	[278]
		Bactericidal	*Erwinia carotovora*, *Pseudomonas syringae*	-	

Staphylococcin, Subtilosin, bacilysin)					
Bacillibactin (and Rhizoctin, Surfactin, Bacillaene, Fengycin Staphylococcin, Subtilosin, bacilysin)	*Bacillus cabrialessi* BH6	Fungicidal	*Rhizoctonia solani*, *Botrytis cinerea*, *Verticillium dahliae*, *Phytophthora infestans*	-	
		Bactericidal	*Erwinia carotovora*, *Pseudomonas syringae*	-	
Bacillibactin (and Surfactin, Bacillaene, Fengycin, Subtilin, Subtilosin, Bacilysin)	*Bacillus subtilis* DH12	Fungicidal	*Rhizoctonia solani*, *Botrytis cinerea*, *Verticillium dahliae*, *Phytophthora infestans*	-	
		Bactericidal	*Pseudomonas syringae*	*Erwinia carotovora*	
Bacillibactin (and Bacilysin, Subtilosin, Fengycin, Bacillaene, Surfactin)	*Bacillus subtilis* EH2	Fungicidal	*Rhizoctonia solani*, *Botrytis cinerea*, *Verticillium dahliae*, *Phytophthora infestans*	-	
		Bactericidal	-	*Erwinia carotovora*, *Pseudomonas syringae*	
Bacillibactin (and Bacilysin, Subtilosin, Fengycin, Bacillaene, Surfactin)	*Bacillus subtilis* EH5	Fungicidal	*Botrytis cinerea*, *Phytophthora infestans*	*Rhizoctonia solani*, *Verticillium dahliae*	
		Bactericidal	*Erwinia carotovora*	*Pseudomonas syringae*	
Bacillibactin (and Surfactin, Bacillaene, Fengycin, Subtilin, Subtilosin, Bacilysin)	*Bacillus subtilis* EH11	Fungicidal	*Rhizoctonia solani*, *Botrytis cinerea*, *Phytophthora infestans*	*Verticillium dahliae*	
		Bactericidal	*Pseudomonas syringae*	*Erwinia carotovora*	
Bacillibactin (and Surfactin, Plantazolicin Macrolactin, Fengycin, Difficidin, Bacillaene, Bacilysin)	*Bacillus velezensis* FH17	Fungicidal	*Rhizoctonia solani*, *Botrytis cinerea*, *Verticillium dahliae*, *Phytophthora infestans*	-	
		Bactericidal	*Erwinia carotovora*, *Pseudomonas syringae*	-	
Bacillibactin (and Fengycin, Difficidin, Surfactin. Macrolactin Bacillaene, Bacilysin)	*Bacillus velezensis* TH16	Fungicidal	*Rhizoctonia solani*, *Botrytis cinerea*, *Verticillium dahliae*, *Phytophthora infestans*	-	
		Bactericidal	*Erwinia carotovora*, *Pseudomonas syringae*	-	

Bacillibactin	*Bacillus endophyticus* FH5	Fungicidal	*Rhizoctonia solani, Verticillium dahliae, Phytophthora infestans*	*Botrytis cinerea*	
		Bactericidal	-	*Erwinia carotovora, Pseudomonas syringae*	
Bacillibactin (and Polymyxin)	*Paenibacillus* sp. EDO6	Fungicidal	*Phytophthora infestans*	*Rhizoctonia solani, Botrytis cinerea, Verticillium dahliae*	
		Bactericidal	-	*Erwinia carotovora, Pseudomonas syringae*	
Bacillibactin (and Bacillaene, Bacilysin, Fengycin, Macrolactin, Bacillomycin, Subtilosin, Surfactin)	*Bacillus inaquosorum* KR2-7	Fungicidal	*Alternaria alternata, Athelia rolfsii, Botrytis cinerea, Rhizoctonia solani, Verticillium albo-atrum*	-	[279]
Bacillibactin (and Surfactin, Fengycin, Rhizocticin, Bacillaene, Bacilysin, Subtilosin)	*Bacillus cabrialesii* TE3	Fungicidal	*Bipolaris sorokiniana*	NT	[280]
Bacillibactin (and Surfactin, Iturin, Bacillaene, Bacilysin)	*Bacillus nakamurai* BDI-IS1	Bactericidal	*Clavibacter michiganensis, Rhodococcus fascians, Pectobacterium carotovorum, Xanthomonas campestris, Pseudomonas cichorii, Pseudomonas fuscovaginae*	-	[281]
		Fungicidal	*Fusarium oxysporum, Botrytis cinerea, Rhizoctonia solani, Aspergillus niger, Pyricularia oryzae, Colletotricum* sp.	-	
Bacillibactin (and Surfactin, Subtilosin, Bacillaene, Bacilysin)	*Bacillus subtilis* BSD	Fungicidal	*Verticillium dahlia, Botrytis cinerea*	-	[282]
Bacillibactin (and Difficidin, Macrolactin, Fengycin, Surfactin,	*Bacillus velezensis* 20507	Fungicidal	*Exserohilum turcicum, Pyricularia oryzae, Fusarium graminearum, Sclerotinia sclerotiorum, Fusarium oxysporum, Fusarium verticillioides*	-	[142]

Butirosin, Bacillaene)					
Bacillibactin (and Iturin, Bacillomycine, Difficidin, Fengycin, Surfactin, Bacillaene, Bacilysin)	*Bacillus* sp. N67	Fungicidal	*Fusarium oxysporum*	-	[283]
Bacillibactin (and Macrolactin, Iturin, Bacillomycine, Difficidin, Fengycin, Surfactin, Bacillaene, Bacilysin)	*Bacillus* sp. WBN06				
Bacillibactin (and Iturin, Bacillomycine, Difficidin, Fengycin, Surfactin, Bacillaene, Bacilysin)	*Bacillus* sp. HN04				
Bacillibactin (and Macrolactin, Iturin, Bacillomycine, Difficidin, Fengycin, Surfactin, Bacillaene, Bacilysin)	*Bacillus* sp. YN1282-2				
Bacillibactin (and Iturin, Bacillomycine, Difficidin, Fengycin, Surfactin, Bacillaene, Bacilysin)	*Bacillus* sp. G9R-3				
Bacillibactin (and Surfactin, Bacillaene, Fengycin, Bacilysin, Subtilosin)	*Bacillus halotolerans* Q2H2	Fungicidal	*Fusarium oxysporum*, *Fusarium commune*, *Fusarium graminearum*, *Fusarium brachygibbosum*, *Rhizoctonia solani*, *Stemphylium solani*	-	[284]

Bacillibactin	*B. amyloliquefaciens* MTCC 12713	Bactericidal	*Staphylococcus aureus, Enterococcus faecalis, Pseudomonas aeruginosa, Klebsiella pneumoniae*	-	[123]
Fengycin (and Iturin, Bacillomycin, Mycosubtilin, Surfactin, Bacilysin, Bacillaene, Macrolactin, Difficidin)	*B. velezensis* SG277	Bactericidal	*Clostridioides difficile*	*Escherichia coli, Salmonella enterica, Bacillus cereus, Klebsiella aerogenes, Staphylococcus aureus, Staphylococcus hominis, Listeria monocytogenes*	[285]
	B. velezensis SG137				
	B. velezensis SG185				
	B. velezensis SG277				
	B. velezensis SG297		*Clostridioides difficile, Staphylococcus aureus*	*Escherichia coli, Salmonella enterica, Bacillus cereus, Klebsiella aerogenes, Staphylococcus hominis, Listeria monocytogenes*	
Fengycin	*Bacillus atrophaeus* NX-12	Fungicidal	*Fusarium oxysporum*	NT	[286]
Fengycin (and Bacillomycin, Surfactin)	*B. amyloliquefaciens* 49	Fungicidal	*Sclerotinia sclerotiorum, Fusarium oxysporum*	-	[287]
Fengycin (and Bacillomycin, Surfactin, Bacilysin)	*B. amyloliquefaciens* 50				
Fengycin (and Bacillomycin, Bacilysin)	*Bacillus sp.* 206				
Fengycin (and Bacillomycin)	*B. thuringiensis* 2026				
Fengycin (and Bacillomycin, Surfactin)	*B. amyloliquefaciens* 2536		*Sclerotinia sclerotiorum*	*Fusarium oxysporum*	
Fengycin (and Bacillomycin, Surfactin, Bacilysin)	*B. amyloliquefaciens* 2784		*Sclerotinia sclerotiorum, Fusarium oxysporum*	-	
Fengycin (and Bacillomycin, Surfactin, Bacilysin)	*B. amyloliquefaciens* 2785				
Fengycin (and Bacillomycin, Surfactin, Bacilysin)	*B. amyloliquefaciens* 2788				

Fengycin (and Bacillomycin, Surfactin, Bacilysin)	*B. amyloliquefaciens* 2791				
Fengycin (and Bacillibactin, Butirosin, Macrolactin, Bacillaene, Difficidin, Surfactin, Bacilysin, Mersacidin)	*Bacillus velezensis* NWUMFkBS10.5	Fungicidal	*Fusarium graminearum, Fusarium culmorum*	-	[288]
		Bactericidal	*Bacillus cereus, Enterococcus faecalis, Klebsiella pneumoniae, Pseudomonas aeruginosa*	-	
Fengycin (and Bacillomycin)	*Bacillus amyloliquefaciens* YN201732	Fungicidal	*Fusarium solani, Erysiphe cichoracearum*	-	[289]
Fengycin (and Iturin, Surfactin)	*Bacillus halotolerans* QTH8	Fungicidal	*Hainesia lythri, Pestalotiopsis sp., Botrytis cinerea, Curvularia lunata, Phyllosticta theaefolia, Fusarium graminearum, Phytophthora nicotianae, Sclerotinia sclerotiorum*	-	[290]
Fengycin (and Surfactin)	*Bacillus subtilis* ZD01	Fungicidal	*Alternaria solani*	NT	[124]
Fengycin (and Bacillibactin, Difficidin, Macrolactin, Butirosin, Bacilysin, Surfactin)	*Bacillus velezensis* FB2	Fungicidal	*Alternaria alternata, Fusarium oxysporum, Fusarium solani*	-	[291]
		Bactericidal	*Escherichia coli, Bacillus cereus, Pseudomonas syringae*	*Klebsiella pneumoniae*	
Fengycin	*Bacillus amyloliquefaciens* LPB-18	Fungicidal	*Aspergillus flavus, Aspergillus niger, Fusarium oxysporum*	-	[292]
		Bactericidal	*Staphylococcus aureus, Salmonella choleraesuis, Bacillus cereus, Escherichia coli, Cronobacter sakazakii, Enterobacter aerogenes, Salmonella typhimurium*	-	
Fengycin (and Bacillomycin, Iturin, Surfactin)	*Bacillus subtilis* UL-1	Fungicidal	*Cryptococcus neoformans, Aspergillus fumigatus, Fusarium oxysporum, Fusarium solani*	*Candida albicans*	[293]
Fengycin (and Iturin, Surfactin)	*B. amyloliquefaciens FZB42*	Fungicidal	*Fusarium oxysporum, Fusarium solan, Fusarium*	-	[143]

	Bacillus sp. NH 217	Fungicidal	*moniliforme, Trichoderma atroviride*		
	B. subtilis NH-100	Fungicidal			
Fengycin (and Iturin, Surfactin, Locillomycin, Butirosin Macrolactin, Bacillaene, Difficidin, Bacillibactin, Bacilysin)	*Bacillus velezensis* Q12	Fungicidal	*Fusarium solani*	-	[123]
		Bactericidal	*Pectobacterium carotovorum, Pectobacterium atrosepticum, Xanthomonas campestris*	-	
Fengycin (and Bacillomycin, Plantazolicin, Surfactin, Butirosin Macrolactin, Bacillaene, Difficidin, Bacillibactin, Bacilysin)	*Bacillus velezensis* US1	Fungicidal	*Fusarium solani*	-	
		Bactericidal	-	*Pectobacterium carotovorum, Pectobacterium atrosepticum, Xanthomonas campestris*	
Fengycin (and Surfactin, Iturin)	*Bacillus velezensis* BSM54	Fungicidal	*Fusarium oxysporum, Sclerotinia sclerotiorum*	-	[294]
	Bacillus velezensis KRF-001				
Fengycin (and Bacillibactin, Laterocidine, Geobacillin, Lichenysin, Butirosin, Schizokinen)	*Bacillus glycinifermentans* MGMM1	Fungicidal	*Fusarium oxysporum, Fusarium graminearum*	-	[295]
Fengycin (and Bacillomycin)	*Bacillus amyloliquefaciens* Q-426	Fungicidal	*Fusarium oxysporum, Colletotrichum lindemuthianum, Coniothyrium diplodiella, Curvularia lunata, Exserohilum turcicum, Fusarium graminearum, Fusarium moniliform, Fusarium semitectum, Mucor hiemolis, Pellicularia sasakii, Penicillium chrysogenum, Rhizoctonia solani, Rhizopus oryzae, Sclerotinia sclerotiorum, Trichoderma viridae*	-	[296]

		Bactericidal	-	*Bacillus subtilis, Escherichia coli*	
Fengycin (and Surfactin, Iturin)	*Bacillus velezensis* NKMV-3	Fungicidal	*Alternaria solani, Fusarium oxysporum, Rhizoctonia solani, Pyricularia oryzae*	*Macrophomina phaseolina*	[297]
Fengycin (and Surfactin, Iturin)	*Bacillus siamensis* m62b	Fungicidal	*Fusarium oxysporum, Cholletotrichum orchidophilum*	-	[144]
	Bacillus siamensis m64				
	Bacillus siamensis t24				
Fengycin (and Surfactin)	*Bacillus cabrialesii* BH5	Fungicidal	*Rhizoctonia solani, Botrytis cinerea, Fusarium culmorum, Pythium ultimum*	-	[298]
Fengycin (and Surfactin, Iturin)	*Bacillus cabrialesii* BH5	Fungicidal	*Ralstonia solanacearum, Fusarium oxysporum*	-	[299]
Fengycin	*Bacillus subtilis* FAJT-4	Fungicidal	*Fusarium oxysporum*	NT	[300]
Fengycin (and Bacillibactin, Fengycin, Iturin, Macrolactin, Bacillaene, Bacilysin, Difficidin, Butirosin, Lantipeptide	*Bacillus velezensis* WB	Fungicidal	*Fusarium oxysporum*	NT	[301]
Fengycin (and Surfactin, Iturin)	*B. subtilis* RMB5	Fungicidal	*Fusarium oxysporum, Fusarium solani, Rhizoctonia solani, Colletotrichum gloeosporioides, Colletotrichum falcatum, Aspergillus niger, Aspergillus flavus, Alternaria alternata, Pythium irregulare, Pythium ultimum.*	-	[302]
	B. subtilis NS1				
	B. subtilis NS2				
	B. subtilis NS4				
	B. subtilis NS6				
Fengycin (and Surfactin, Iturin)	*Bacillus velezensis* Bs006	Fungicidal	*Fusarium oxysporum*	NT	[145]
Fengycin (and Surfactin, Mycosubtilin)	*Bacillus subtilis* FJ3	Fungicidal	*Fusarium oxysporum, Aspergillus flavus, Aspergillus niger, Rhizopus oryzae*	-	[303]
Fengycin (and Surfactin, Iturin)	*Bacillus amyloliquefaciens* DHA55	Fungicidal	*Fusarium oxysporum, Didymella bryoniae, Sclerotinia sclerotiorum,*	-	[304]

			Fusarium graminearum, Rhizoctonia solani		
Fengycin (and Bacilysin, Subtilosin, Surfactin)	*Bacillus subtilis* SEM-9	Fungicidal	*Fusarium sambucinum, Fusarium catenulatum, Fusarium oxysporum, Fusarium proliferatum, Fusarium equiseti, Fusarium graminearum*	-	[305]
Fengycin (and Surfactin, Iturin)	*Bacillus amyloliquefaciens* NCPSJ7	Fungicidal	*Fusarium oxysporum*	NT	[306]
Fengycin (and Surfactin)	*B. subtilis* NCIB 3610	Fungicidal	*Botrytis cinerea*	NT	[307]
Fengycin (and Surfactin, Bacillomycin Mycosubtilin, Kurstakin)	*Bacillus mojavensis* I4	Fungicidal	*Fusarium solani*	NT	[308]
Fengycin (and Surfactin, Bacillaene, Bacillibactin, Subtilosin, Basilysin)	*Bacillus subtilis* MBI 600	Fungicidal	*Fusarium oxysporum, Pythium aphanidermatum*	NT	[309]
Fengycin (and Surfactin, Iturin)	*Bacillus halotolerans* BM1-3	Fungicidal	*Neocosmospora solani, Fusarium brachygibbosum, Fusarium equiseti*	*Fusarium oxysporum*	[152]
Fengycin (and Iturin)	*Bacillus amyloliquefaciens* BM3-4	Fungicidal	*Neocosmospora solani, Fusarium brachygibbosum, Fusarium equiseti*	*Fusarium oxysporum*	[152]
Fengycin (and Iturin)	*Bacillus halotolerans* BM3-5	Fungicidal	*Neocosmospora solani, Fusarium oxysporum, Fusarium brachygibbosum, Fusarium equiseti*	-	[152]
Fengycin (and Iturin)	*Bacillus halotolerans* BM4-1	Fungicidal	*Neocosmospora solani, Fusarium oxysporum, Fusarium brachygibbosum, Fusarium equiseti*	-	[152]
Fengycin (and Bacillomycin, Subtilosin)	*Stenotrophomonas maltophilia* GH1-5	Fungicidal	*Neocosmospora solani*	*Fusarium oxysporum, Fusarium brachygibbosum, Fusarium equiseti*	[152]
Fengycin (and Bacillomycin, Iturin, Subtilosin)	*Bacillus subtilis* GH3-8	Fungicidal	*Neocosmospora solani, Fusarium oxysporum, Fusarium brachygibbosum, Fusarium equiseti*	-	[152]
Fengycin	*Bacillus velezensis* HNAH 17806	Fungicidal	*Fusarium solani*	NT	[310]

Fengycin (and Surfactin, Iturin)	*Bacillus* sp. INECOL-4742<br>*Bacillus* sp. INECOL-5927	Fungicidal	*Fusarium solani*, *Fusarium kuroshium,*	NT	[311]
Fengycin (and Surfactin, Iturin)	*Bacillus subtilis* DHA41	Fungicidal	*Fusarium oxysporum*, *Didymella bryoniae*, *Sclerotinia sclerotiorum*, *Fusarium graminearum*, *Rhizoctonia solani*	-	[312]
Fengycin (and Surfactin, Iturin, Bacilysin, Bacillomycin)	*Bacillus* sp. XY-2-3<br>*Bacillus* sp. XY-13<br>*Bacillus* sp. GJ-1-15	Fungicidal	*Fusarium oxysporum*, *Fusarium solani*, *Rhizoctonia solani*	-	[313]
Fengycin (and Surfactin, Iturin)<br>Fengycin (and Kurstakin, Surfactin, Iturin)	*Bacillus amyloliquefaciens* SS-12.6<br>*Bacillus amyloliquefaciens* SS-38.4	Bactericidal	*Xanthomonas arboricola*, *Pseudomonas syringae*	NT	[314]
Fengycin (and Surfactin, Iturin)	*Bacillus amyloliquefaciens* CCeRi1-002	Fungicidal	*Fusarium solani*, *Fusarium kuroshium*	-	[315]
Fengycin (and Surfactin, Iturin)	*Bacillus velezensis* Bs006	Fungicidal	*Fusarium oxysporum*	-	[316]
Fengycin (and Surfactin, Iturin)	*Bacillus velezensis* Bs006	Fungicidal	*Fusarium oxysporum*	NT	[317]
Fengycin (and Surfactin)	*Bacillus cereus* TSH77	Fungicidal	*Fusarium solani*	NT	[141]
Fengycin (and Surfactin, Myriocin)	*Bacillus velezensis* LZN01	Fungicidal	*Fusarium oxysporum*	NT	[318]
Fengycin (and Surfactin, Bacilysin, Bacillibactin, Sublancin, Bacillaene, Subtilosin)	*Bacillus subtilis* SZMC 6179J	Fungicidal	*Alternaria solani*, *Alternaria alternata*, *Botrytis cinerea*, *Phytophthora infestans and Sclerotinia sclerotiorum*, *Fusarium solani*, *Fusarium oxysporum*, *Bipolaris bicolor*	-	[319]
Fengycin (and Mycosubtilin)	*Bacillus pumilus* St 1T2	Fungicidal	*Aspergillus carbonarius*, *Botrytis cinerea*, *Eutypa lata*	*Aspergillus flavus*, *Aspergillus niger*, *Aspergillus ochraceus*, *Clonostachys rosea*,	[147]

				Fusarium equiseti, Fusarium oxysporum, Fusarium solani	
Fengycin (and Mycosubtilin, Difficidin, Macrolactin)	*Bacillus subtilis* E1Pv	Fungicidal	*Aspergillus carbonarius, Aspergillus niger, Aspergillus ochraceus, Botrytis cinerea, Clonostachys rosea, Fusarium equiseti, Fusarium oxysporum, Fusarium solani, Eutypa lata*	*Aspergillus flavus*	
Fengycin (and Mycosubtilin, Bacillibactin, Difficidin, Macrolactin, Iturin)	*Bacillus velezensis* BPVs2<br>*Bacillus velezensis* BAHs1<br>*Bacillus velezensis* BTAs3<br>*Bacillus velezensis* LFF MYM 5		*Aspergillus carbonarius, Aspergillus flavus, Aspergillus niger, Aspergillus ochraceus, Botrytis cinerea, Clonostachys rosea, Fusarium equiseti, Fusarium oxysporum, Fusarium solani, Eutypa lata*	-	
Fengycin (and Iturin)	*Bacillus sp.* P34	Fungicidal	*Aspergillus flavus, Aspergillus niger, Aspergillus phoenicis, Bipolaris sorokiniana, Fusarium oxysporum Fusarium graminearum*	-	[320]
Fengycin (and Surfactin, Iturin)	*Bacillus sp.* P37				
Fengycin (and Surfactin, Bacillomycin)	*Bacillus amyloliquefaciens* SYBC H47	Fungicidal	*Aspergillus niger, Mucor racemosus, Fusarium oxysporum, Botryosphaeria dothidea*	-	[321]
Fengycin (and Surfactin, Bacillomycin)	*Bacillus velezensis* GB03<br>*Bacillus velezensis* FZB42<br>*Bacillus subtilis* BSn5	Fungicidal	*Fusarium oxysporum*	-	[322]
Fengycin (and Surfactin, Iturin)	*Bacillus velezensis* FJAT-46737	Bactericidal	*Ralstonia solanacearum, Escherichia coli*	-	[323]
		Fungicidal	*Fusarium oxysporum*	NT	
Fengycin (and Surfactin, Difficidin, Bacillaene, Macrolactin, Butirosin, Bacilysin, Bacillibactin)	*Bacillus amyloliquefaciens* WS-8	Fungicidal	*Botrytis cinerea*	NT	[324]

Fengycin (and Surfactin)	*Bacillus subtilis* NM4	Fungicidal	*Alternaria solani*	NT	[325]
Fengycin (and Surfactin, Iturin)	*Bacillus amyloliquefaciens* SR1	Fungicidal	*Rhizoctonia solani, Alternaria solani, Fusarium oxysporum, Sclerotium rolfsii, Macrophomina phaseolina, Aspergillus niger*	-	[326]
Fengycin (and Surfactin, Iturin)	*Bacillus velezensis* N23	Fungicidal	*Colletotrichum sp., Botrytis cinerea, Exserohilum turcicum, Alternaria solani, Fusarium oxysporum*	-	[327]
Fengycin (and Andalusicin, Surfactin, Difficidin, Bacillaene, Macrolactin, Bacilysin, Bacillibactin)	*Bacillus velezensis* AMR25	Bactericidal	*Erwinia billingiae, Pantoae agglomerans, Xantomonas campestris*	-	[328]
		Fungicidal	*Botrytis cinerea, Fusarium avenacea,*	-	
Fengycin (and Surfactin, Iturin)	*Bacillus subtilis* XF-1	Fungicidal	*Plasmodiophora brassicae, Fusarium solani*	-	[329]
Fengycin (and Surfactin, Bacillomycin)	*Bacillus methylotrophicus* XT1 CECT 8661	Fungicidal	*Botrytis cinerea*	NT	[330]
Fengycin (and Surfactin, Iturin, Pumilacidin)	*Bacillus methylotrophicus* DCS1	Fungicidal	*Colletotrichum sp., Rhizoctonia bataticola, Verticillium dahlia, Fusarium oxysporum*	-	[331]
Fengycin (and Surfactin, Bacillomycin)	*Bacillus amyloliquefaciens* CMS5<br>*Bacillus amyloliquefaciens* CMR12	Fungicidal	*Fusarium oxysporum, Fusarium solani*	-	[332]
Fengycin (and Iturin)	*Bacillus amyloliquefaciens* NJN-6	Fungicidal	*Fusarium oxysporum*	NT	[333]
Fengycin (and Surfactin)	*Bacillus subtilis* BBG201	Fungicidal	*Botrytis cinerea*	NT	[334]
Fengycin	*Bacillus subtilis* C2	Fungicidal	*Fusarium solani*	NT	[335]
Fengycin (and Surfactin, Bacilysin)	*Bacillus subtilis* NCIB3610	Fungicidal	*Fusarium oxysporum*	NT	[336]
Fengycin (and Surfactin)	*Bacillus subtilis* PCL1605	Fungicidal	*Fusarium oxysporum, Rosellinia necatrix, Rhizoctonia solani*	*Phytophthora cinnamomi, Pythium ultimum, Sclerotium rolfsii*	[337]

Fengycin (and Surfactin, Iturin)	*Bacillus subtilis* PCL1608		*Fusarium oxysporum*, *Phytophthora cinnamomi*, *Pythium ultimum*, *Rosellinia necatrix*, *Rhizoctonia solani*, *Sclerotium rolfsii*	-	
Fengycin (and Surfactin)	*Bacillus subtilis* PCL1610		*Fusarium oxysporum*, *Rosellinia necatrix*, *Rhizoctonia solani*, *Sclerotium rolfsii*	*Phytophthora cinnamomi*, *Pythium ultimum*	
Fengycin (and Surfactin, Iturin)	*Bacillus subtilis* PCL1612		*Fusarium oxysporum*, *Phytophthora cinnamomi*, *Pythium ultimum*, *Rosellinia necatrix*, *Rhizoctonia solani*, *Sclerotium rolfsii*	-	
Fengycin (and Surfactin, Iturin)	*Bacillus velezensis* CCTCC 2022571	Bactericidal	*Pectobacterium carotovorum*	NT	[338]
Fengycin (and Iturin, Bacillomycin)	*Bacillus amyloliquefaciens* S2536	Fungicidal	*Fusarium oxysporum*, *Scherotinia sclerotiorum*	-	[339]
Fengycin (and Iturin, Bacillomycin, Bacilysin)	*Bacillus amyloliquefaciens* S2784		*Scherotinia sclerotiorum*	*Fusarium oxysporum*	
	Bacillus amyloliquefaciens S2785		*Fusarium oxysporum*, *Scherotinia sclerotiorum*	-	
	Bacillus amyloliquefaciens S2787		*Fusarium oxysporum*	*Scherotinia sclerotiorum*	
	Bacillus amyloliquefaciens S2788		*Fusarium oxysporum*, *Scherotinia sclerotiorum*	-	
	Bacillus amyloliquefaciens S2791		*Fusarium oxysporum*, *Scherotinia sclerotiorum*	-	
Fengycin (and Surfactin)	*Bacillus subtilis* S2896		-	*Fusarium oxysporum*, *Scherotinia sclerotiorum*	
Fengycin	*Bacillus amyloliquefaciens* 6256	Fungicidal	*Botrytis cinerea*	NT	[340]
Fengycin (and Iturin)	*Bacillus amyloliquefaciens* LBM5006	Fungicidal	*Aspergillus niger*, *Aspergillus phoencis*, *Aspergillus flavus*, *Apiosordaria* sp., *Bipolaris sorokiniana*, *Cercosporina sojina*, *Diplodia* sp., *Fusarium oxysporum*, *Fusarium graminearum*	*Promopsis* sp., *Rhizoctonia* sp., *Verticillium albatrum*	[341]
Fengycin (and Surfactin)	*Bacillus mojavensis* BL1	Fungicidal	*Botrytis cinerea*	-	[342]

Fengycin (and Surfactin)	*Brevibacterium halotolerans* BT5				
Fengycin (and Surfactin)	*Bacillus amyloliquefaciens* BF11				
Fengycin	*Bacillus* sp. YCFE4	Bactericidal	*Enterococcus faecalis, Staphylococcus epidermidis Staphylococcus aureus, Listeria monocytogenes, Escherichia coli*	*Salmonella enterica*	[148]
		Fungicidal	-	*Colletotrichium gloeosporoides, Fusarium oxysporum*	
Fengycin (and Surfactin, Bacillomycin)	*Bacillus subtilis* UMAF6614	Bactericidal	*Xanthomonas campestris, Pectobacterium carotovorum*	*Pseudomonas syringae, Acidovorax avenae*	[343]
Fengycin (and Surfactin, Iturin)	*Bacillus subtilis* UMAF6619		*Xanthomonas campestris*	*Pseudomonas syringae, Pectobacterium carotovorum, Acidovorax avenae*	
Fengycin (and Surfactin, Iturin)	*Bacillus subtilis* UMAF6639		*Xanthomonas campestris, Pectobacterium carotovorum, Acidovorax avenae*	*Pseudomonas syringae*	
Fengycin (and Surfactin, Bacillomycin)	*Bacillus subtilis* UMAF8561		*Xanthomonas campestris*	*Pseudomonas syringae, Pectobacterium carotovorum, Acidovorax avenae*	
Fengycin (and Surfactin Bacilysocin)	*Bacillus subtilis* NCD-2	Fungicidal	*Botrytis cinerea*	-	[344]
Fengycin (and Bacillomycin)	*Bacillus amyloliquefaciens* SQR9	Fungicidal	*Fusarium oxysporum*	NT	[345]
Fengycin (and Surfactin, Bacillomycin, Difficidin, Bacillaene, Macrolactin, Bacilysin)	*Bacillus amyloliquefaciens* SQR9	Bactericidal	*Pseudomonas syringae*	NT	[346]
		Fungicidal	*Botrytis cinerea*	NT	
Fengycin	*Bacillus atrophaeus* CAB-1	Fungicidal	*Botrytis cinerea, Sphaerotheca fuliginea*	-	[347]
Fengycin (and Surfactin, Iturin)	*Bacillus subtilis* GA1	Fungicidal	*Botrytis cinerea, Fusarium oxysporum, Fusarium graminearum, Pythium ultimum, Pythium ultimum, Rhizoctonia solani, Aspergillus niger, Aspergillus*	-	[348]

			flavus, Trichoderma harzanium, Trichoderma reesei, Mucor sp.		
Fengycin (and Surfactin, Iturin)	*Bacillus amyloliquefaciens* PPL	Fungicidal	*Fusarium oxysporum*	NT	[349]
Fengycin (and Bacillomycin, Bacilysin, Surfactin)	*Bacillus velezensis* HeN-7	Fungicidal	*Bipolaris sorokiniana*	-	[350]
Bacillibactin (and Bacitracin, Butirosin, Lichenysin Haloduracin)	*Bacillus paralicheniformis* TRQ65	Fungicidal	*Bipolaris sorokiniana*	NT	[351]
Bacillibactin (and Surfactin, Fengycin, Rhizocticin, Bacillaene, Bacilysin, Subtilosin)	*Bacillus cabrialesii* TE3	Fungicidal	*Bipolaris sorokiniana*	NT	[352]
Fengycin (and Surfactin, Iturin)	*Bacillus* sp. Kol L6	Fungicidal	*Fusarium culmorum, Fusarium oxysporum*	-	[353]
Fengycin (and Surfactin, Bacilysin, Bacillibactin, Bacillaene, Difficidin, Macrolactin, Iturin, Lactococcin)	*Bacillus velezensis* LBUM279	Fungicidal	*Botrytis cinerea, Sclerotinia sclerotiorum, Fusarium oxysporum, Fusarium culmorum, Fusarium sporotrichoides, Nigrospora sphaerica, Nigrospora oryzae, Alternaria alternata, Phoma* sp., *Cercospora* sp.	-	[151]
Fengycin (and Surfactin, Bacilysin, Bacillibactin, Bacillaene, Difficidin, Macrolactin, Iturin)	*Bacillus velezensis* LBUM1082				
Fengycin (and Surfactin, Bacilysin, Bacillibactin, Bacillaene, Subtilosin, Sublancin)	*Bacillus subtilis* LBUM979		*Botrytis cinerea, Sclerotinia sclerotiorum, Fusarium culmorum, Nigrospora sphaerica, Nigrospora oryzae, Alternaria alternata, Phoma* sp., *Cercospora* sp.	*Fusarium oxysporum, Fusarium sporotrichoides*	
Fengycin (and Iturin,		Bactericidal	-	*Pectobacterium carotovorum*	[153]

Surfactin, Bacilysin, Difficidin, Macrolactin)	*Bacillus amyloliquefaciens* 17A-B3	Fungicidal	*Alternaria solani*, *Fusarium solani*, *Rhizoctonia solani*	-	
Fengycin (and Bacillomycin, Surfactin)	*Bacillus licheniformis* 7B-B41	Bactericidal	-	*Pectobacterium carotovorum*	
		Fungicidal	*Alternaria solani*, *Fusarium solani*	*Rhizoctonia solani*	
Fengycin (and Bacillomycin)	*Bacillus licheniformis* 8B-B32	Bactericidal	-	*Pectobacterium carotovorum*	
		Fungicidal	*Alternaria solani*, *Fusarium solani*	*Rhizoctonia solani*	
Fengycin (and Bacilysin)	*Bacillus pumilus* 15A-B8	Bactericidal	-	*Pectobacterium carotovorum*	
		Fungicidal	*Alternaria solani*	*Rhizoctonia solani*, *Fusarium solani*	
Fengycin (and Bacilysin)	*Bacillus pumilus* 44R-B1	Bactericidal	-	*Pectobacterium carotovorum*	
		Fungicidal	*Alternaria solani*, *Rhizoctonia solani*	*Fusarium solani*	
Fengycin	*Bacillus pumilus* 44R-B2	Bactericidal	-	*Pectobacterium carotovorum*	
		Fungicidal	*Alternaria solani*, *Rhizoctonia solani*	*Fusarium solani*	
Fengycin (and Bacillomycin, Surfactin, Bacilysin)	*Bacillus subtilis* 15A B91	Bactericidal	-	*Pectobacterium carotovorum*	
		Fungicidal	*Alternaria solani*, *Rhizoctonia solani*	*Fusarium solani*	
Fengycin (and Surfactin, Bacillibactin, Bacillaene, Paenibacillin)	*Bacillus subtilis* BSn5	Bactericidal	*Pectobacterium carotovorum*	NT	[354]
Fengycin (and Surfactin, Bacillaene, Bacillibactin, Subtilosin, Bacilysin, Subtilomycin)	*Bacillus subtilis* 73	Fungicidal	*Botrytis cinerea*	*Fusarium oxysporum*, *Fusarium graminearum*	[146]
Fengycin (and Surfactin, Bacillaene, Bacillibactin, Subtilosin, Bacilysin)	*Bacillus subtilis* 75		*Botrytis cinerea*, *Fusarium oxysporum*, *Fusarium graminearum*	-	
Fengycin (and Surfactin, Bacillaene, Bacillibactin, Subtilosin, Bacilysin, Subtilomycin)	*Bacillus subtilis* MB8_B1		*Botrytis cinerea*, *Fusarium oxysporum*, *Fusarium graminearum*	-	

Fengycin (and Surfactin, Bacillaene, Bacillibactin, Subtilosin, Bacilysin, Sublancin)	*Bacillus subtilis* MB8_B7		*Botrytis cinerea*, *Fusarium oxysporum*, *Fusarium graminearum*	-	
Fengycin (and Surfactin, Bacillaene, Bacillibactin, Subtilosin, Bacilysin, Subtilomycin)	*Bacillus subtilis* MB8_B10		*Botrytis cinerea*, *Fusarium oxysporum*, *Fusarium graminearum*	-	
Fengycin (and Surfactin, Bacillaene, Bacillibactin, Subtilosin, Bacilysin, Subtilomycin)	*Bacillus subtilis* MB9_B1		*Botrytis cinerea*, *Fusarium oxysporum*, *Fusarium graminearum*	-	
Fengycin (and Surfactin, Bacillaene, Bacillibactin, Subtilosin, Bacilysin, Subtilomycin)	*Bacillus subtilis* MB9_B4		*Botrytis cinerea*, *Fusarium oxysporum*, *Fusarium graminearum*	-	
Fengycin (and Surfactin, Bacillaene, Bacillibactin, Subtilosin, Bacilysin, Subtilomycin)	*Bacillus subtilis* MB9_B6		*Botrytis cinerea*	*Fusarium oxysporum*, *Fusarium graminearum*	
Fengycin (and Surfactin, Bacillaene, Bacillibactin, Subtilosin, Bacilysin, Subtilin)	*Bacillus subtilis* P5_B1		*Botrytis cinerea*, *Fusarium oxysporum*, *Fusarium graminearum*	-	
Fengycin (and Surfactin, Bacillibactin, Subtilosin, Bacilysin)	*Bacillus subtilis* P5_B2		-	*Botrytis cinerea*, *Fusarium oxysporum*, *Fusarium graminearum*	
Fengycin (and Surfactin, Bacillaene, Bacillibactin, Subtilosin,	*Bacillus subtilis* P8_B1		*Botrytis cinerea*, *Fusarium oxysporum*, *Fusarium graminearum*	-	

Bacilysin, Sublancin)					
Fengycin (and Bacillibactin, Butirosin, Lichenysin, Lichenicidin)	*Bacillus licheniformis* P8_B2		-	*Botrytis cinerea*, *Fusarium oxysporum*, *Fusarium graminearum*	
Fengycin (and Surfactin, Bacillaene, Bacillibactin, Subtilosin, Bacilysin, Sublancin)	*Bacillus subtilis* P8_B3		*Botrytis cinerea*, *Fusarium oxysporum*, *Fusarium graminearum*	-	
Fengycin (and Surfactin, Bacillaene, Bacillibactin, Subtilosin, Bacilysin)	*Bacillus subtilis* P9_B1		*Botrytis cinerea*, *Fusarium oxysporum*, *Fusarium graminearum*	-	
Fengycin (and Surfactin, Bacillaene, Bacillibactin, Macrolactin, Difficidin)	*Bacillus velezensis* Yao	Fungicidal	*Fusarium solani*	-	[355]
Fengycin (and Surfactin, Bacillaene, Bacillibactin, Macrolactin, Difficidin, Bacilysin, Butirosin)	*Bacillus velezensis* 9D-6	Bactericidal	*Clavibacter michiganensis*, *Bacillus cereus*, *Pantoea agglomerans*, *Ralstonia solanacearum*, *Xanthomonas campestris*, *Xanthomonas euvesicatoria*	*Erwinia amylovora*, *Pseudomonas syringae*	[356]
		Fungicidal	*Alternaria solani*, *Cochliobolus carbonum*, *Fusarium oxysporum*, *Fusarium solani*, *Gibberella pulicaris*, *Gibberella zeae*, *Monilinia fructicola*, *Pyrenochaeta terrestris*, *Rhizoctonia solani*	*Pythium mamillatum*	
Fengycin (and Surfactin, Bacillaene, Bacillibactin, Macrolactin, Difficidin, Bacilysin, Butirosin Locillomycin,)	*Bacillus amyloliquefaciens* XJ5	Fungicidal	*Alternaria solani*,	NT	[357]
Fengycin (and Surfactin, Bacillaene, Macrolactin,	*Bacillus velezensis* YXDHD1-7	Fungicidal	*Alternaria solani*, *Fusarium oxysporum*	*Ralstonia solanacearum*	[358]

Difficidin, Bacilysin, Butirosin, Amylocyclicin, Mersacidin, Macrobrevin, Bryostatin)					
Fengycin (and Surfactin, Bacillomycin)	*Bacillus siamensis* NKIT9	Fungicidal	*Rhizoctonia solani, Verticillium lateritium, Botrytis cinerea, Alternaria solani, Fusarium oxysporum*	-	[359]
Fengycin (and Surfactin, Iturin)	*Bacillus amyloliquefaciens* SS-12.6<br>*Bacillus amyloliquefaciens* SS-38.4<br>*Bacillus pumilus Bacillus amyloliquefaciens* SS-10.7	Bactericidal	*Pseudomonas syringae*	-	[360]
Fengycin (and Kijanimicin)	*Lysinibacillus boronitolerans* Unnamed	Bactericidal	*Bacillus pumilus, Bacillus velezensis, Pseudomonas syringae, Xanthomonas axonopodis*	*Staphylococcus saprophyticus, B. subtilis*	[361]
Fengycin (and Surfactin, Iturin, Bacillaene, Macrolactin, Difficidin)	*Bacillus amyloliquefaciens FZB42*	Bactericidal	*Pseudomonas syringae*	NT	[362]
Fengycin (and Surfactin, Bacillaene, Bacillibactin, Macrolactin, Difficidin, Bacilysin, Butirosin, Kalimantacin, Micrococcin, Plantazolicin)	*Bacillus velezensis* RC116	Fungicidal	*Fusarium oxysporum, Ralstonia solanacearum*	-	[363]
Fengycin (and Surfactin, Bacillaene, Bacillibactin, Macrolactin, Difficidin, Bacilysin, Butirosin)	*Bacillus velezensis* Ag75	Fungicidal	*Rhizoctonia solani, Macrophomina phaseolina, Fusarium solani*	-	[364]
Fengycin (and Lichenysin,	*Bacillus altitudinis*	Bactericidal	*Listeria monocytogenes*	*Salmonella enterica, Escherichia coli*	[365]

Enterocin, Bacilysin, Bottromycin)	UTK D1-0055	Fungicidal	-	*Saccharomyces cerevisiae*, *Botrytis cinerea*, *Hyperdermium pulvinatum*	
Fengycin (and Surfactin, Iturin)	*Bacillus subtilis* PTS-394	Fungicidal	*Fusarium oxysporum*, *Fusarium solani*, *Ralstonia solanacearum*	-	[366]
Fengycin (and Surfactin, Bacilysin, Thailanstatin, Subtilosin, Subtilin, Bacillibactin)	*Bacillus subtilis* PBs 12	Fungicidal	*Magnaporthe grisea*, *Sclerotium rolfsii*, *Fusarium solani*, *Alternaria alternata*, *Ganoderma*	-	[367]
Fengycin (and Bacillibactin, Geobacillin, Lichenysin, Butirosin, Schizokinen, Bacitracin)	*Bacillus paralichenifo rmis* PBl 36				
Fengycin (and Surfactin, Iturin)	*Bacillus* sp. RB150	Fungicidal	*Fusarium solani*	-	[368]
Fengycin (and Surfactin)	*Bacillus velezensis* OEE1	Fungicidal	*Fusarium solani*, *Fusarium avenaceum*, *Fusarium sulphureum*, *Cladosporium cladosporioides* *Botrytis cinerea*	-	[369]
Fengycin (and Surfactin, Iturin)	*Bacillus subtilis* SPB1	Fungicidal	*Fusarium solani*	NT	[370]
Fengycin (and Surfactin, Iturin, Bacillomycin)	*Bacillus licheniformis* XNRB-3	Fungicidal	*Fusarium oxysporum*, *Fusarium moniliforme*, *Fusarium proliferatum*, *Fusarium solani*	-	[371]
Fengycin (and Surfactin)	*Bacillus amyloliquefac iens* RS-25	Fungicidal	*Botrytis cinerea*	-	[372]
Fengycin (and Surfactin, Iturin)	*Bacillus subtilis* Pnf-4				
Fengycin (and Surfactin, Iturin)	*Bacillus subtilis* Z-14				
Fengycin (and Iturin)	*Bacillus licheniformis* MG-4				
Fengycin (and Surfactin, Iturin)	*Bacillus velezensis* BBC023	Fungicidal	*Botrytis cinerea*	-	[373]

	Bacillus velezensis BBC047				
Fengycin (and Surfactin)	*Bacillus subtilis* MBI 600	Fungicidal	*Botrytis cinerea*	-	[374]
Fengycin (and Surfactin, Bacillibactin, Difficidin, Bacilysin, Bacillaene)	*Bacillus velezensis* CMRP 4489	Fungicidal	*Sclerotinia sclerotiorum, Macrophomina phaseolina, Botrytis cinerea*	-	[375]
Fengycin (and Surfactin, Bacillomycin, Bacillibactin, Bacillaene, Difficidin, Macrolactin, Amylocyclicin)	*Bacillus velezensis* 83	Fungicidal	*Botrytis cinerea*	NT	[376]
Fengycin (and Surfactin, Bacillibactin, Macrolactin, Bacillaene, Difficidin, Bacilysin)	*Bacillus velezensis* E	Fungicidal	*Botrytis cinerea, Fusarium oxysporum, Rhizoctonia solani, Alternaria solani, Phytophthora capsica*	-	[377]
Fengycin (and Bacillibactin, Bacilysin, Lichenysin, Plantazolicin)	*Bacillus safensis* RGM 2450	Fungicidal	*Botrytis cinerea, Colletotrichum acutatum*	*Fusarium oxysporum, Phytophtora cinnamomi*	[378]
Fengycin (and Surfactin, Bacillibactin, Bacillaene, Aurantinin, Bacillomycin, Amylocyclin)	*Bacillus siamensis* RGM 2529		*Botrytis cinerea, Colletotrichum acutatum, Fusarium oxysporum, Phytophtora cinnamomi*	-	
Fengycin (and Surfactin, Bacillaene, Mycosubtilin, Bacillibactin, Bacilycin, Subtilosin)	*Bacillus halotolerans* Hil4	Fungicidal	*Botrytis cinerea*	-	[379]
Fengycin (and Surfactin, Bacillaene, Bacillibactin, Macrolactin,	*Bacillus subtilis* 168	Fungicidal	*Rhizoctonia solani, Fusarium oxysporum, Botrytis cinerea, Alternaria alternata, Cochliobolus heterostrophus, and Nigrospora oryzae*	-	[380]

Difficidin, Iturin)					
Petrobactin (and Bacillibactin, Fengycin)	*Bacillus cereus* Z4	Fungicidal	*Phytophthora nicotianae*	NT	[381]
Petrobactin (and Locillomycin, Macrobrevin)	*Paenibacillus ehimensis isolate* MZ921932	Bactericidal	*Staphylococcus aureus, Escherichia coli, Klebsiella pneumoniae*	-	[382]
		Fungicidal	*Candia albicans, Candia auris*	NT	
Petrobactin (and Fengycin, Cerecidin)	*Lysinibacillus xylanilyticus* t26	Fungicidal	*Pythium ultimum, Rhizoctonia solani, Fusarium oxysporum*	-	[150]
Paenilamicin	*Paenibacillus larvae* DSM25430	Bactericidal	*Bacillus megaterium*	NT	[125]
	Paenibacillus larvae DSM25430	Bactericidal	*Bacillus megaterium*	NT	[383]
	Paenibacillus larvae DSM25430	Bactericidal	*Bacillus megaterium, Bacillus licheniformis, Paenibacillus pastoris*	*Paenibacillus alvei, Bacillus subtilis*	[126]
		Fungicidal	*Fusarium oxysporum, Saccharomyces cerevisiae*	-	
	Paenibacillus larvae unnamed	Bactericidal	*Staphylococcus aureus*	NT	[113]
		Fungicidal	*Sporobolomyces salmonicolor, Aspergillus fumigatus*	NT	
Paenilamicin (and Baptifoline, Dehydromorroniaglycone, Isoleucinopine Sophoramine, Melazolide, Rengyoside, Iedoglucomide, Aeruginopeptin)	*Paenibacillus larvae* DJ4	Bactericidal	*Escherichia coli, Bacillus subtilis, Pseudomonas aeruginosa, Klebsiella pneumoniae*	-	[384]
Paenilamicin (and Baptifoline, Dehydromorroniaglycone, Isoleucinopine Sophoramine, Aspersecosteroid, Rengyoside,	*Paenibacillus larvae* DJ9	Bactericidal			

Iedoglucomide, Aeruginopeptin					
Paenilamicin (and Petrobactin, Bacillibactin, Fengycin)	*Bacillus thuringiensis* NBAIR BtAr	Fungicidal	*Sclerotium rolfsii*	NT	[385]
Paeninodin	*Paenibacillus dendritiformis* C454	Bactericidal	-	*Bacillus subtilis, Micrococcus flavus, Burkholderia rhizoxinica, Sphingobium japonicum, Xanthomonas citri* pv. *mangiferaeindicae, Bacillus cereus, Bacillus megaterium, Bacillus amyloliquefaciens*	[128]
Paeninodin (and Paenibacillin, Paenilan, Sactipeptides, Thiazole-oxazole modified microcin)	*Paenibacillus polymyxa* Kp 10	Bactericidal	*Listeria monocytogenes*	NT	[386]
Paeninodin (and Paenibacillin, Fusaricidin, Bacillibactin, Tridecaptin, Paenilipoheptin, Brevicidine, Paenilipoheptin, Lacunalide, Polymyxin, Paenicidin	*Paenibacillus peoriae* ZBSF16	Fungicidal	*Gloeosporium fructigrum, Botrytis cinerea, Diaporthe eres, Alternaria viticola, Fusarium oxysporum, Aspergillus niger, Pestalotiopsis clavispora*, and	-	[129]
		Bactericidal	*Allorhizobium vitis*	-	
Butyrolactol A	*Streptomyces rochei* S785-16	Fungicidal	*Aspergillus fumigatus, Trichophyton mentagrophytes, Candida albicans, Saccharomyces cerevisiae, Cryptococcus neoformans*	*Candida tropicalis*	[130]
Cerecyclin	*Bacillus cereus* DDD103	Bactericidal	*Bacillus cereus, Bacillus firmus, Bacillus subtilis, Bacillus thuringiensis, Bacillus amyloliquefaciens, Bacillus pumilus,*	*Escherichia coli, Salmonella enterica, Pseudomonas aeruginosa, Pseudomonas putida*	[127]

			Staphylococcus aureus, Listeria monocytogenes, Enterococcus faecalis		

Table S14. The list of patented biopreparations using *B. mycoides* strains and patented biopesticides against species inhibited by the strain b12.3 described in the current study. Phytopathogens that wich growth was suppressed when adding the strain b12.3 are highlighted in green color.

Organism/mixture	Target activity	Reference
Bacillus mycoides isolate J	*Cercospora beticola*	US20070224179A1
Bacillus mycoides JYZ-SD5	*Alternaria alternata, Alternaria tenuissima*	CN109355228B
Bacillus mycoides isolate J, *Bacillus mojavensis,* MSU 203-7	*Cladosporium corrigendum*	US20070224179A1
Bacillus mycoldes 683	*Fusarium sambucinum, Erwinia aroideae*	RU1771639C
Bacillus mycoides WWM09	*Sphaerotheca fuliginea*	CN105002120A
Penicillium steckii IBWF104-06	*Alternaria solani, Botrytis cinerea, Phytophthora infestans*	US20180127840A1
Paenibacillus (mixture of strains Lu16774, Lu17007, Lu17015, BD-62)	*Alternaria solani, Phytophthora infestans, Botrytis cinerea, Sclerotinia sclerotiorum*	CN109152367B
Bacillus belgii K01	*Alternaria solani, Botrytis cinerea, Verticillium dahlia, Pseudomonas syringae, Pectobacterium carotovorum, Xanthomonas citri*	CN112746043B
Bacillus velezensis RTI301	*Alternaria solani, Botrytis cinereaI, Cercospora sojina, Fusarium graminearum, Stagonospora nodorum, Magnaporthe grisea, Fusarium oxysporum*	US20180020676A1
Bacillus amyloliquefaciens RTI472	*Alternaria solani, Cercospora sojina, Fusarium graminearum, Fusarium oxysporum, Moniina fructicola, Magnaporihe grisea, Scierotinia scierotiorum*	US20170196226A1
Bacillus amyloliquefaciens subsp. *plantarum* 71, *Paenibacillus polymyxa* To99	*Xanthomonas campestris, Xanthomonas perforans, Xanthomonas gardneri*	US20210337806A1
Pseudozyma aphidis L12	*Xanthomonas campestris, Clavibacter michiganensis, Botrytis cinerea, Alternaria brassicicola*	US9161545B2
Bacillus amyloliquefaciens DAIJU-SIID2550	*Xanthomonas campestris, Clavibacter michiganensis, Erwinia carotovora, Pyricularia grisea, Cochliobolus miyabeanus, Alternaria brassicae*	EP1719410B1
Streptomyces griseoflavus NMG6-3-9 CGMCC No. 3441	*Xanthomonas campestris, Pseudomonas syringae, Fusarium solani, Fusarium graminearum,*	CN101822272B

	Stemphylium botryosum, Fusarium oxysporum, Erwinia carotovora	
Bacillus methylotrophicus DR-08	*Xanthomonas campestris, Pseudomonas syringae, Ralstonia solanacearum, Xanthomonas oryzae, Xanthomonas arboricola, Botrytis cinerea, Endothia parasitica, Rhizoctonia solani*	US11528912B2
Bacillus sp. F727	*Clavibacter michiganensis, Xanthomonas campestris, Xanthomonas arboricola, Erwinia carotovora, Botrytis cinerea*	EP2885398B1
Bacillus mojavensis R3B	*Clavibacter michiganensis, Pseudomonas syringae, Xanthomonas vesicatoria, Pythium debaryanum, Alternaria alternata*	ES2702497T3
Bacillus subtilus ITI-2	*Clavibacter michiganensis*	WO2020069438A1
Streptomyces microflavus NMG2-4-8,	*Clavibacter michiganensis, Xanthomonas campestris, Ralstonia solanacearum, Erwinia carotovora, Rhizoctonia solani, Stemphylium botryosum*	CN101864378B
Bacillus velezensis G341, *Lysinibacillus sphaericus* TC1	*Clavibacter michiganensis, Ralstonia solanacearum, Burkholderia glumae, Sclerotinia sclerotiorum, Rhizoctonia solani, Botrytis cinerea*	KR102015051B1
Bacillus methylotrophicus XT1	*Pectobacterium atrosepticum, Xanthomonas campestris, Ralstonia solanacearum, Botrytis cinerea*	US20170215429A1
Pseudomonas spp. 0617-T307, 0917-T305, 0917-T306, 0917-T307, 0118-T319, 0318-T327, 0418-T328 (cell broth with produced metabolites)	*Pectobacterium atrosepticum, Clavibacter michiganensis, Erwinia amylovora, Xanthomonas axonopodis, Botrytis cinerea, Venturia inaqualis*	US20230165260A1
Pseudomonas chlororaphis subsp. *aurantiaca* 1214-CHY4	*Pectobacterium atrosepticum, Clavibacter michiganensis, Xanthomonas campestris, Pseudomonas syringae, Pectobacterium carotovorum, Erwinia amylovora, Dickeya dadantii, Botrytis cinerea*	US20220232834A1
Bacillus amyloliquefaciens Ab8b	*Pectobacterium atrosepticum, Pectobacterium carotovorum, Pectobacterium wasabiae, Dickeya solani*	RU2673155C1
Pseudomonas putida CECT8538	*Pectobacterium atrosepticum, Botrytis aclada, Rhizoctonia solani, Pythium ultimum, Sclerotinia sclerotiorum*	JP2020515273A

Table S15. Available commercial formulations based on active beneficial microorganisms. Listed are the names of the biopreparation and the microbial agents within it, the mechanisms of defensive activities, target pathogens, and plants. The data was taken from the US patent US20210337806A1. Phytopathogens that wich growth was suppressed when adding the strain b12.3 are highlighted in green color.

Product: Bioagent	Mode of action	Target pathogens	Crop	Company- Registered and commercialized reference
Actinovate: *Streptomyces lydicus*	Antibiosis	Soilborne disease	Greenhouse and nursery crops, turf	Natural Industries Inc., USA, McSpadden Gardner B.B., 2002
Bacillus SPP®: *Bacillus* spp .	Antibiosis	*Xanthomonas campestris* pv. *vesicatoria*, *Pseudomonas syringae* pv. *syringae*	Several crops	Bio InsumosNativa Ltda., Chili, Cawoy H., et al., 2011
Ballad®: *Bacillus pumilus*	Antibiosis, competition, growth promotion, resistance induction	*Xanthomonas* spp.	Cereals, oil plants, sugar beet	AgraQuest Inc., USA, Cawoy H., et al., 2011
BioPro®: *Bacillus subtilis* BsBD170	Antibiosis	*Erwinia amylovora*	Apple, pear, oriental pear , quince, loquat	Germany, USA, Zeller W., 2006
Biosubtilin: *Bacillus subtilis*	Antibiosis, competition	*Xanthomonas* spp.	Cotton, cereals, ornamental plants, and vegetable crops	Biotech International Ltd., India Cawoy H., et al., 2011
BlightBanA506™: *Pseudomonas fluorescens* A506	Competition of sites and nutrients	*Erwinia amylovora* and russet-inducing bacteria	Almond, apple, apricot, tomato blueberry, cherry, peach, pear, potato, strawberry	NuFarm Inc., USA, Cawoy H., et al., 2011
BloomtimeBiological™: *Pantoea agglomerans* (syn . *Erwinia herbicola*)	Antibiosis (herbicolin, pantocin A and B)	*Erwinia amylovora*	Apple, pear, oriental pear, quince, loquat	Northwest Agri Products, USA, Grantastein, 2014
Blossom Protect™: *Aureobasidium pullulans* (strains DSM 14940 and DSM 14941)	Antibiosis	*Erwinia amylovora*	Apple, crabapple, pear, oriental pear, quince, loquat	Bio-ferm GmbH, Austria, Germany, USA, New Zealand, Grantastein, 2014 and Kunz et al., 2011
Botrycid®: *Burkholderia cepacia*	Antibiosis	*Xanthomonas* sp., *Erwinia* sp., *Agrobacterium* sp.	Several crops	Safer Agrobiologicos,

				Colombia, Cawoy H. et al., 2011
Cease®: *Bacillus subtilis*	Antibiosis	*Xanthomonas* spp.	Several crops	BioWorks Inc., USA, Mexico, Cawoy H. et al., 2011
Cedomon™: *Pseudomonas chlororaphis*	Antibiosis	*Pseudomonas syringae*	Barley and oats, other cereals	BioAgri AB, Sweden, McSpadden Gardner B.B., 2002
Companion®: *Bacillus subtilis*	Antibiosis, competition, growth promotion, resistance induction	*Xanthomonas campestris*, *Pseudomonas syringae*	Cotton, bean, pea, soybean, peanut, corn, and others	Growth Products Ltd., USA, Cawoy H. et al., 2011
Galltrol™: *Agrobacterium radiobacter* 84	Antibiosis	Agrobacterium tumefaciens	Fruit, nut, and ornamental nursery stock	AgBioChem Inc., USA, McSpadden Gardner B.B., 2002
Messenger™: *Erwinia amylovora* (HrpNharpin protein)	Plant activator	Wide spectrum (general activation)	Field ornamental and vegetable crop	EDEN Bioscience Corporation, USA, McSpadden Gardner B.B., 2002
Nacillus Pro™: *Brevibacillus parabrevis* N4, *Bacillus subtilis* N5, *Bacillus cereus* N6, *Bacillus cereus* N7	Antibiosis, competition	*Pseudomonas syringae* pv. *syringae* , *P. syringae* pv. tomato, *Xanthomonas campestris* pv. *vesicatoria*, *X. campestris* pv. *coralina*, *Xanthomonas juglandis*, *Clavibacter michiganensis* subsp. *michiganensis*, *Acetobacter* sp., *Erwinia caratovora*	Tomato, peppers, cucurbits, walnut, peanut, hop, leafy vegetables	Bio InsumosNativa Ltda., Chili Valdes et al., 2012
Nogall™: *Agrobacterium radiobacter* K1026	Antibiosis	*Agrobacterium tumefaciens*	Fruit, nut, and ornamental nursery stock	Bio-care Technology, Australia, New BioProducts Inc., Australia, USA, McSpadden Gardner B.B., 2002

Serenade®: *Bacillus subtilis* QST 713	Antibiosis	*Xanthomonas* spp. (bacterial spot) , *Xanthomonas campestris* (walnut blight), *Erwinia amylovora* (fire blight)	Grape, apples, pear, banana, cherry, walnut, peanut, hop, leafy vegetables, tomato, peppers, cucurbits, mango, bean, onion garlic, potato, broccoli, carrot	AgraQuest Inc., Chile, USA, New Zealand, Mexico, Japan, Israel, Costa Rica, Philippines, Guatemala, Honduras, Argentina, Italy, France, Turkey, Switzerland, Korea, Ecuador, Peru, Cawoy H. et al., 2011

Supporting Tables Description

Table S1. Forward and reverse primers used for the *gyrB* gene sequencing.

Table S2. The list of biosynthetic gene clusters detected by the antiSMASH software. Presented are genomic coordinates of the regions, the description of the closest known cluster, and the respective similarity score.

Table S3. Biosynthetic gene clusters found in the genome of the strain b12.3 using the DeepBGC utility. Shown are genomic coordinates of the regions, accuracy score, predicted activity and chemical class of the produced moiety, and the list of PfamA domains applied to detect a cluster.

Table S4. The closest 50 reference genomes from the RefSeq database with respective ANI values relative to the genome of the strain b12.3.

Table S5. The metadata of the closest reference strains deposited in the RefSeq database. The data was taken from NCBI Assembly and BioSample databases. Presented are the properties of the assembly, taxonomic attribution, isolation source, geographic origin, and host of the isolates.

Table S6. Main genomic features (toxins and biosynthetic gene clusters) in the analyzed dataset (strain b12.3 with 50 closest reference genomes according to ANI estimates). Presented are the properties of the assemblies (the number of contigs, completeness, and contamination level). The list of known/putative BGCs coupled with predicted biological activities and homologs of the insecticidal factors, and the list of affected hosts according to the BPPRC database. The respective lists of the features are separated by semicolon and agree with the following pattern: *<cluster/toxin>*:*<identity score>*.

Table S7. Feature-wise characteristics of genomes in the context of predicted toxins, BGCs, and biological activities. Provided are the total number of a particular loci/activity and mean identity with the closest homolog in case the loci (known BGCs/toxin-encoding loci) are described.

Table S8. The cluster-wise total amount of biosynthetic gene clusters in the genomes within the studied dataset.

Table S9. The abundance of loci encoding virulence factors. The total amount of genes including paralogs are considered.

Table S10. The existing information on the 50 closest (relative to the strain b12.3) *B. mycoides* isolates selected according to ANI values. Presented are qualitative categories present in the articles describing the strains. The references to the studies are marked by DOI.

Table S11. The structured information from articles in the NCBI Pubmed database related to *B. mycoides* isolates in the context of produced metabolites and their activities (searching term – *Bacillus mycoides*). Lusted are the tested pathogens (bacteria/fungi) and BGCs presented in the strains' genomes and/or identified by chromatography methods. The presence/absence of the activity is marked with "+" and "-" symbols, respectively. In case the feature was not reported, the cell contains "NT". The green background highlights BGCs/pathogens that were studied in the current work.

Table S12. Short summaries from studies related to the microbiota of Lake Baikal with surrounding territories. Listed are isolation sites of the bacteria, taxonomic assignments of selected microorganisms, and the types of research. The green color highlights the same categories as those conducted in this research whereas the yellow color represents partial similarity, i.e., isolation from sediments (but not soil) and examining *Bacillus* sp. of unknown species.

Table S13. The spectrum of bactericidal and/or fungicidal activities of strains from the *Bacillaceae* family. The criteria of inclusion for the research were the presence of at least one BGC and tested phytopathogens described in this study. If the isolate was already pre-tested on a certain pathogen before conducting the research, the respective cell contains "NT". In case the strain was active/non-active against all the pathogens tested, the "-" symbol is used. The entities mentioned in this study (BGCs/pathogens) are highlighted in green.

Table S14. The list of patented biopreparations using *B. mycoides* strains and patented biopesticides against species inhibited by the strain b12.3 described in the current study. Phytopathogens that wich growth was suppressed when adding the strain b12.3 are highlighted in green color.

Table S15. Available commercial formulations based on active beneficial microorganisms. Listed are the names of the biopreparation and the microbial agents within it, the mechanisms of defensive activities, target pathogens, and plants. The data was taken from the US patent US20210337806A1. Phytopathogens whose growth was suppressed when adding the strain b12.3 are highlighted in green color.

Supplementary References

Here, the references represent those unmentioned in the main text. Other numbers refer to those presented in the manuscript.

159. Méndez Acevedo, M.; Carroll, L.M.; Mukherjee, M.; Mills, E.; Xiaoli, L.; Dudley, E.G.; Kovac, J. Novel Effective *Bacillus cereus* Group Species "*Bacillus clarus*" Is Represented by Antibiotic-Producing Strain ATCC 21929 Isolated from Soil. *mSphere* **2020**, *5*, https://doi.org/10.1128/mSphere.00882-20.

160. Jun'ichi Shoji, H.; Mikao Mayama, I.; Shinzo Matsuura, I.; Kouichi Matsumoto, T.; Yoshiharu Wakisaka, T. Antibiotic 60-6 and Production Thereof. Patent US3923979A, 2 December **1974**.

161. Hung, J.-C.; Huang, T.-P.; Huang, J.; Chang, C.J.; Jan, F.-J. The Efficacy of Orange Terpene and *Bacillus mycoides* Strain BM103 on the Control of Periwinkle Leaf Yellowing Phytoplasma. *Plant Dis* **2024**, https://doi.org/10.1094/PDIS-07-24-1547-RE.

162. Elamary, R.; Salem, W.M. Optimizing and Purifying Extracellular Amylase from Soil Bacteria to Inhibit Clinical Biofilm-Forming Bacteria. *PeerJ* **2020**, *8*, e10288, https://doi.org/10.7717/peerj.10288.

163. Gambhir, N.; Paul, A.; Qiu, T.; Combs, D.B.; Hosseinzadeh, S.; Underhill, A.; Jiang, Y.; Cadle-Davidson, L.E.; Gold, K.M. Non-Destructive Monitoring of Foliar Fungicide Efficacy with Hyperspectral Sensing in Grapevine. *Phytopathology* **2024**, *114*, 464–473, https://doi.org/10.1094/PHYTO-02-23-0061-R.

164. Athukorala, S.N.P.; Fernando, W.G.D.; Rashid, K.Y. Identification of Antifungal Antibiotics of *Bacillus* Species Isolated from Different Microhabitats Using Polymerase Chain Reaction and MALDI-TOF Mass Spectrometry. *Can J Microbiol* **2009**, *55*, 1021–1032, https://doi.org/10.1139/W09-067.

165. Yuan, X.; Gdanetz, K.; Outwater, C.A.; Slack, S.M.; Sundin, G.W. Evaluation of Plant Defense Inducers and Plant Growth Regulators for Fire Blight Management Using Transcriptome Studies and Field Assessments. *Phytopathology* **2023**, *113*, 2152–2164, https://doi.org/10.1094/PHYTO-04-23-0147-KC.

166. Ambas, I.; Buller, N.; Fotedar, R. Isolation and Screening of Probiotic Candidates from Marron, *Cherax cainii* (Austin, 2002) Gastrointestinal Tract (GIT) and Commercial Probiotic Products for the Use in Marron Culture. *J Fish Dis* **2015**, *38*, 467–476, https://doi.org/10.1111/jfd.12257.

167. Michaud, M.; Martinez, C.; Simao-Beaunoir, A.-M.; Bélanger, R.R.; Tweddell, R.J. Selection of Antagonist Microorganisms Against *Helminthosporium solani*, Causal Agent of Potato Silver Scurf. *Plant Dis* **2002**, *86*, 717–720, https://doi.org/10.1094/PDIS.2002.86.7.717.

168. Kong, G.A.; Kochman, J.K.; Brown, J.F. Phylloplane Bacteria Antagonistic to the Sunflower Pathogen *Alternaria helianthi*. *Australasian Plant Pathology* **1997**, *26*, 85, https://doi.org/10.1071/AP97014.

169. Luo, T.; Hou, S.; Yang, L.; Qi, G.; Zhao, X. Nematodes Avoid and Are Killed by *Bacillus mycoides*-Produced Styrene. *J Invertebr Pathol* **2018**, *159*, 129–136, https://doi.org/10.1016/j.jip.2018.09.006.

170. Fravel, D.R. Biocontrol of Tobacco Brown-Spot Disease by *Bacillus cereus* subsp. *mycoides* in a Controlled Environment. *Phytopathology* **1977**, *77*, 930, https://doi.org/10.1094/Phyto-67-930.

171. de la Huerta-Bengoechea, P.; Gil-Serna, J.; Melguizo, C.; Ramos, A.J.; Prim, M.; Vázquez, C.; Patiño, B. Biocontrol of Mycotoxigenic Fungi Using Bacteria Isolated from Ecological Vineyard Soils. *Journal of Fungi* **2022**, *8*, 1136, https://doi.org/10.3390/jof8111136.

172. Sandilya, S.P.; Jeevan, B.; Subrahmanyam, G.; Dutta, K.; Vijay, N.; Bhattacharyya, N.; Chutia, M. Co-Inoculation of Native Multi-Trait Plant Growth Promoting Rhizobacteria Promotes Plant Growth and Suppresses *Alternaria* Blight Disease in Castor (*Ricinus communis* L.). *Heliyon* **2022**, *8*, e11886, https://doi.org/10.1016/j.heliyon.2022.e11886.

173. Singh, R.K.; Singh, P.; Li, H.-B.; Song, Q.-Q.; Guo, D.-J.; Solanki, M.K.; Verma, K.K.; Malviya, M.K.; Song, X.-P.; Lakshmanan, P.; et al. Diversity of Nitrogen-Fixing Rhizobacteria Associated with Sugarcane: A Comprehensive Study of Plant-Microbe Interactions for Growth Enhancement in *Saccharum* spp. *BMC Plant Biol* **2020**, *20*, 220, https://doi.org/10.1186/s12870-020-02400-9.

174. Irvin, A.D. The Inhibition of *Listeria monocytogenes* by an Organism, Resembling *Bacillus mycoides*, Present in Normal Silage. *Res Vet Sci* **1969**, *10*, 106–108, https://doi.org/10.1016/S0034-5288(18)34499-0.

175. Czaban, J.; Ksiezniak, A.; Wróblewska, B.; Paszkowski, W.L. An Attempt to Protect Winter Wheat against *Gaeumannomyces graminis* var. *tritici* by the Use of Rhizobacteria *Pseudomonas fluorescent* and *Bacillus mycoides*. *Pol J Microbiol* **2004**, *53*, 101–110.

176. Piacenza, E.; Presentato, A.; Zonaro, E.; Lemire, J.A.; Demeter, M.; Vallini, G.; Turner, R.J.; Lampis, S. Antimicrobial Activity of Biogenically Produced Spherical Se-nanomaterials Embedded in Organic Material against *Pseudomonas aeruginosa* and *Staphylococcus aureus* Strains on Hydroxyapatite-coated Surfaces. *Microb Biotechnol* **2017**, *10*, 804–818, https://doi.org/10.1111/1751-7915.12700.

177. Ambas, I.; Suriawan, A.; Fotedar, R. Immunological Responses of Customised Probiotics-Fed Marron, *Cherax tenuimanus*, (Smith 1912) When Challenged with *Vibrio mimicus*. *Fish Shellfish Immunol* **2013**, *35*, 262–270, https://doi.org/10.1016/j.fsi.2013.04.026.

178. Guetsky, R.; Shtienberg, D.; Elad, Y.; Dinoor, A. Combining Biocontrol Agents to Reduce the Variability of Biological Control. *Phytopathology* **2001**, *91*, 621–627, https://doi.org/10.1094/PHYTO.2001.91.7.621.

179. Azarova, I.N.; Parfenova, V. V; Baram, G.I.; Terkina, I.A.; Pavlova, O.N.; Suslova, M.I. Degradation of Bis(2-Ethylhexyl)Phthalate by Microorganisms of Water and Sediments of the Selenga River and Baikal Lake under Experimental Conditions. *Prikl Biokhim Mikrobiol* **2003**, *39*, 665–669, https://doi.org/10.1023/A:1026282502521.

180. Cabello-Yeves, P.J.; Zemskaya, T.I.; Rosselli, R.; Coutinho, F.H.; Zakharenko, A.S.; Blinov, V. V.; Rodriguez-Valera, F. Genomes of Novel Microbial Lineages Assembled from the Sub-Ice Waters of Lake Baikal. *Appl Environ Microbiol* **2018**, *84*, https://doi.org/10.1128/AEM.02132-17.

181. Bel'kova, N.L.; Parfenova, V. V.; Suslova, M.Yu.; Ahn, T.S.; Tazaki, K. Biodiversity and Activity of the Microbial Community in the Kotelnikovsky Hot Springs (Lake Baikal). *Biology Bulletin* **2005**, *32*, 549–555, https://doi.org/10.1007/s10525-005-0142-y.

182. Pavlova, O.N.; Zemskaya, T.I.; Gorshkov, A.G.; Parfenova, V. V.; Suslova, M.Yu.; Khlystov, O.M. Study on the Lake Baikal Microbial Community in the Areas of the Natural Oil Seeps. *Appl Biochem Microbiol* **2008**, *44*, 287–291, https://doi.org/10.1134/S0003683808030101.

183. Parfenova, V. V.; Terkina, I.A.; Kostornova, T.Ya.; Nikulina, I.G.; Chernykh, V.I.; Maksimova, E.A. Microbial Community of Freshwater Sponges in Lake Baikal. *Biology Bulletin* **2008**, *35*, 374–379, https://doi.org/10.1134/S1062359008040079.

184. Pavlova, O.N.; Tupikin, A.E.; Chernitsyna, S.M.; Bukin, Y.S.; Lomakina, A. V.; Pogodaeva, T. V.; Nikonova, A.A.; Bukin, S. V.; Zemskaya, T.I.; Kabilov, M.R. Description and Genomic Analysis of the First Facultatively Lithoautotrophic, Thermophilic Bacteria of the Genus Thermaerobacter Isolated from Low-Temperature Sediments of Lake Baikal. *Microb Ecol* **2023**, *86*, 1604–1619, https://doi.org/10.1007/s00248-023-02182-1.

185. Butina, T. V; Bukin, Y.S.; Krasnopeev, A.S.; Belykh, O.I.; Tupikin, A.E.; Kabilov, M.R.; Sakirko, M. V; Belikov, S.I. Estimate of the Diversity of Viral and Bacterial Assemblage in the Coastal Water of Lake Baikal. *FEMS Microbiol Lett* **2019**, *366*, https://doi.org/10.1093/femsle/fnz094.

186. Haro-Moreno, J.M.; Cabello-Yeves, P.J.; Garcillán-Barcia, M.P.; Zakharenko, A.; Zemskaya, T.I.; Rodriguez-Valera, F. A Novel and Diverse Group of *Candidatus* Patescibacteria from Bathypelagic Lake Baikal Revealed through Long-Read Metagenomics. *Environ Microbiome* **2023**, *18*, 12, https://doi.org/10.1186/s40793-023-00473-1.

187. Podosokorskaya, O.A.; Elcheninov, A.G.; Novikov, A.A.; Merkel, A.Y.; Kublanov, I. V. *Fontisphaera Persica* Gen. Nov., Sp. Nov., a Thermophilic Hydrolytic Bacterium from a Hot Spring of Baikal Lake Region, and Proposal of *Fontisphaeraceae* fam. nov., and *Limisphaeraceae* fam. nov. within the Limisphaerales ord. nov. (Verrucomicrobiota). *Syst Appl Microbiol* **2023**, *46*, 126438, https://doi.org/10.1016/j.syapm.2023.126438.

188. Soutourina, O.A.; Semenova, E.A.; Parfenova, V. V.; Danchin, A.; Bertin, P. Control of Bacterial Motility by Environmental Factors in Polarly Flagellated and Peritrichous Bacteria Isolated from Lake Baikal. *Appl Environ Microbiol* **2001**, *67*, 3852–3859, https://doi.org/10.1128/AEM.67.9.3852-3859.2001.

189. Evseev, P.; Tikhonova, I.; Krasnopeev, A.; Sorokovikova, E.; Gladkikh, A.; Timoshkin, O.; Miroshnikov, K.; Belykh, O. *Tychonema* sp. BBK16 Characterisation: Lifestyle, Phylogeny and Related Phages. *Viruses* **2023**, *15*, 442, https://doi.org/10.3390/v15020442.

190. Chernogor, L.; Bakhvalova, K.; Belikova, A.; Belikov, S. Isolation and Properties of the Bacterial Strain *Janthinobacterium* sp. SLB01. *Microorganisms* **2022**, *10*, 1071, https://doi.org/10.3390/microorganisms10051071.

191. Zakharova, Y.R.; Galachyants, Y.P.; Kurilkina, M.I.; Likhoshvay, A. V.; Petrova, D.P.; Shishlyannikov, S.M.; Ravin, N. V.; Mardanov, A. V.; Beletsky, A. V.; Likhoshway, Y. V. The Structure of Microbial Community and Degradation of Diatoms in the Deep Near-Bottom Layer of Lake Baikal. *PLoS One* **2013**, *8*, e59977, https://doi.org/10.1371/journal.pone.0059977.

192. Chernogor, L.; Eliseikina, M.; Petrushin, I.; Chernogor, E.; Khanaev, I.; Belikov, S.I. *Janthinobacterium* sp. Strain SLB01 as Pathogenic Bacteria for Sponge *Lubomirskia baikalensis*. *Pathogens* **2022**, *12*, 8, https://doi.org/10.3390/pathogens12010008.

193. Shchapova, E.; Nazarova, A.; Vasilyeva, U.; Gurkov, A.; Ostyak, A.; Mutin, A.; Adelshin, R.; Belkova, N.; Timofeyev, M. Cellular Immune Response of an Endemic Lake Baikal Amphipod to Indigenous *Pseudomonas* sp. *Marine Biotechnology* **2021**, *23*, 463–471, https://doi.org/10.1007/s10126-021-10039-2.

194. Belikov, S.; Belkova, N.; Butina, T.; Chernogor, L.; Martynova-Van Kley, A.; Nalian, A.; Rorex, C.; Khanaev, I.; Maikova, O.; Feranchuk, S. Diversity and Shifts of the Bacterial Community Associated with Baikal Sponge Mass Mortalities. *PLoS One* **2019**, *14*, e0213926, https://doi.org/10.1371/journal.pone.0213926.

195. Kadnikov, V. V.; Mardanov, A. V.; Beletsky, A. V.; Shubenkova, O. V.; Pogodaeva, T. V.; Zemskaya, T.I.; Ravin, N. V.; Skryabin, K.G. Microbial Community Structure in Methane Hydrate-Bearing Sediments of Freshwater Lake Baikal. *FEMS Microbiol Ecol* **2012**, *79*, 348–358, https://doi.org/10.1111/j.1574-6941.2011.01221.x.

196. Mikhailov, I.S.; Zakharova, Yu.R.; Galachyants, Yu.P.; Usoltseva, M. V.; Petrova, D.P.; Sakirko, M. V.; Likhoshway, Ye. V.; Grachev, M.A. Similarity of Structure of Taxonomic Bacterial Communities in the Photic Layer of Lake Baikal's Three Basins Differing in Spring Phytoplankton Composition and Abundance. *Dokl Biochem Biophys* **2015**, *465*, 413–419, https://doi.org/10.1134/S1607672915060198.

197. Podosokorskaya, O.A.; Elcheninov, A.G.; Novikov, A.A.; Kublanov, I. V. *Fontivita pretiosa gen*. nov., sp. nov., a Thermophilic Planctomycete of the Order *Tepidisphaerales* from a Hot Spring of Baikal Lake Region. *Syst Appl Microbiol* **2022**, *45*, 126375, https://doi.org/10.1016/j.syapm.2022.126375.

198. Chernitsyna, S.M.; Elovskaya, I.S.; Bukin, S. V.; Bukin, Yu.S.; Pogodaeva, T. V.; Kwon, D.A.; Zemskaya, T.I. Genomic and Morphological Characterization of a New *Thiothrix* Species from a Sulfide Hot Spring of the Zmeinaya Bay (Northern Baikal, Russia). *Antonie Van Leeuwenhoek* **2024**, *117*, 23, https://doi.org/10.1007/s10482-023-01918-w.

199. Pavlova, O.N.; Zemskaya, T.I.; Gorshkov, A.G.; Kostornova, T.Ya.; Khlystov, O.M.; Parfenova, V. V. Comparative Characterization of Microbial Communities in Two Regions of Natural Oil Seepage in Lake Baikal. *Biology Bulletin* **2008**, *35*, 287–293, https://doi.org/10.1134/S1062359008030096.

200. Dagurova, O.P.; Namsaraev, B.B.; Kozyreva, L.P.; Zemskaya, T.I.; Dulov, L.E. Bacterial Processes of the Methane Cycle in Bottom Sediments of Lake Baikal. *Microbiology (N Y)* **2004**, *73*, 202–210, https://doi.org/10.1023/B:MICI.0000023990.71983.c1.

201. Kaluzhnaya, O. V.; Itskovich, V.B. Phylogenetic Diversity of Microorganisms Associated with the Deep-Water Sponge *Baikalospongia intermedia*. *Russ J Genet* **2014**, *50*, 667–676, https://doi.org/10.1134/S1022795414060052.

202. Voytsekhovskaya, I. V.; Axenov-Gribanov, D. V.; Murzina, S.A.; Pekkoeva, S.N.; Protasov, E.S.; Gamaiunov, S. V.; Timofeyev, M.A. Estimation of Antimicrobial Activities and Fatty Acid Composition of Actinobacteria Isolated from Water Surface of Underground Lakes from Badzheyskaya and Okhotnichya Caves in Siberia. *PeerJ* **2018**, *6*, e5832, https://doi.org/10.7717/peerj.5832.

203. Terkina, I.A.; Parfenova, V. V.; Ahn, T.S. Antagonistic Activity of Actinomycetes of Lake Baikal. *Appl Biochem Microbiol* **2006**, *42*, 173–176, https://doi.org/10.1134/S0003683806020104.

204. Lomakina, A. V.; Pogodaeva, T. V.; Morozov, I. V.; Zemskaya, T.I. Microbial Communities of the Discharge Zone of Oil- and Gas-Bearing Fluids in Low-Mineral Lake Baikal. *Microbiology (N Y)* **2014**, *83*, 278–287, https://doi.org/10.1134/S0026261714030126.

205. Petrushin, I.; Belikov, S.; Chernogor, L. Cooperative Interaction of *Janthinobacterium* sp. SLB01 and *Flavobacterium* sp. SLB02 in the Diseased Sponge *Lubomirskia baicalensis*. *Int J Mol Sci* **2020**, *21*, 8128, https://doi.org/10.3390/ijms21218128.

206. Shishlyannikova, T.A.; Kuzmin, A. V.; Fedorova, G.A.; Shishlyannikov, S.M.; Lipko, I.A.; Sukhanova, E. V.; Belkova, N.L. Ionofore Antibiotic Polynactin Produced by *Streptomyces* sp. 156A Isolated from Lake Baikal. *Nat Prod Res* **2017**, *31*, 639–644, https://doi.org/10.1080/14786419.2016.1217203.

207. Sobolevskaya, M.P.; Terkina, I.A.; Buzoleva, L.S.; Li, I.A.; Kusaikin, M.I.; Verigina, N.S.; Mazeika, A.N.; Shevchenko, L.S.; Burtseva, Yu. V.; Zvyagintseva, T.N.; et al. Biologically Active Compounds from Lake Baikal Streptomycetes. *Chem Nat Compd* **2006**, *42*, 82–87, https://doi.org/10.1007/s10600-006-0041-6.

208. Sokolova, T.G.; Kostrikina, N.A.; Chernyh, N.A.; Kolganova, T. V.; Tourova, T.P.; Bonch-Osmolovskaya, E.A. *Thermincola carboxydiphila* gen. nov., sp. nov., a Novel Anaerobic, Carboxydotrophic, Hydrogenogenic Bacterium from a Hot Spring of the Lake Baikal Area. *Int J Syst Evol Microbiol* **2005**, *55*, 2069–2073, https://doi.org/10.1099/ijs.0.63299-0.

209. Zhilina, T.N.; Kevbrin, V. V.; Tourova, T.P.; Lysenko, A.M.; Kostrikina, N.A.; Zavarzin, G.A. *Clostridium alkalicellum* sp. nov., an Obligately Alkaliphilic Cellulolytic Bacterium from a Soda Lake in the Baikal Region. *Microbiology (N Y)* **2005**, *74*, 557–566, https://doi.org/10.1007/s11021-005-0103-y.

210. Kaluzhnaya, O. V.; Krivich, A.A.; Itskovich, V.B. Diversity of 16S RRNA Genes in Metagenomic Community of the Freshwater Sponge *Lubomirskia baicalensis*. *Russ J Genet* **2012**, *48*, 855–858, https://doi.org/10.1134/S1022795412070058.

211. Safronova, V.I.; Sazanova, A.L.; Kuznetsova, I.G.; Belimov, A.A.; Andronov, E.E.; Chirak, E.R.; Popova, J.P.; Verkhozina, A. V.; Willems, A.; Tikhonovich, I.A. *Phyllobacterium zundukense* sp. nov., a Novel Species of Rhizobia Isolated from Root Nodules of the Legume Species *Oxytropis triphylla* (Pall.) Pers. *Int J Syst Evol Microbiol* **2018**, *68*, 1644–1651, https://doi.org/10.1099/ijsem.0.002722.

212. Parfenova, V. V.; Gladkikh, A.S.; Belykh, O.I. Comparative Analysis of Biodiversity in the Planktonic and Biofilm Bacterial Communities in Lake Baikal. *Microbiology (N Y)* **2013**, *82*, 91–101, https://doi.org/10.1134/S0026261713010128.

213. Safronova, V.; Belimov, A.; Sazanova, A.; Chirak, E.; Kuznetsova, I.; Andronov, E.; Pinaev, A.; Tsyganova, A.; Seliverstova, E.; Kitaeva, A.; et al. Two Broad Host Range Rhizobial Strains Isolated From Relict Legumes Have Various Complementary Effects on Symbiotic Parameters of Co-Inoculated Plants. *Front Microbiol* **2019**, *10*, https://doi.org/10.3389/fmicb.2019.00514.

214. Likhoshvay, A.; Lomakina, A.; Grachev, M. The Complete Alk Sequences of *Rhodococcus erythropolis* from Lake Baikal. *Springerplus* **2014**, *3*, 621, https://doi.org/10.1186/2193-1801-3-621.

215. Kaluzhnaya, O. V.; Itskovich, V.B. Distinctive Features of the Microbial Diversity and the Polyketide Synthase Genes Spectrum in the Community of the Endemic Baikal Sponge *Swartschewskia papyracea*. *Russ J Genet* **2016**, *52*, 38–48, https://doi.org/10.1134/S1022795416010099.

216. Gladkikh, A.S.; Belykh, O.I.; Klimenkov, I. V.; Tikhonova, I. V. Nitrogen-Fixing Cyanobacterium *Trichormus variabilis* of the Lake Baikal Phytoplankton. *Microbiology (N Y)* **2008**, *77*, 726–733, https://doi.org/10.1134/S0026261708060118.

217. Mikhailov, I.S.; Zakharova, Y.R.; Bukin, Y.S.; Galachyants, Y.P.; Petrova, D.P.; Sakirko, M. V.; Likhoshway, Y. V. Co-Occurrence Networks Among Bacteria and Microbial Eukaryotes of Lake Baikal During a Spring Phytoplankton Bloom. *Microb Ecol* **2019**, *77*, 96–109, https://doi.org/10.1007/s00248-018-1212-2.

218. Galachyants, A.D.; Krasnopeev, A.Y.; Podlesnaya, G.V.; Potapov, S.A.; Sukhanova, E.V.; Tikhonova, I.V.; Zimens, E.A.; Kabilov, M.R.; Zhuchenko, N.A.; Gorshkova, A.S.; et al. Diversity of Aerobic Anoxygenic Phototrophs and Rhodopsin-Containing Bacteria in the Surface Microlayer, Water Column and Epilithic Biofilms of Lake Baikal. *Microorganisms* **2021**, *9*, 842, https://doi.org/10.3390/microorganisms9040842.

219. Galachyants, A.D.; Tomberg, I. V.; Sukhanova, E. V.; Shtykova, Y.R.; Suslova, M.Yu.; Zimens, E.A.; Blinov, V. V.; Sakirko, M. V.; Domysheva, V.M.; Belykh, O.I. Bacterioneuston in Lake Baikal: Abundance, Spatial and Temporal Distribution. *Int J Environ Res Public Health* **2018**, *15*, 2587, https://doi.org/10.3390/ijerph15112587.

220. Lomakina, A. V.; Bukin, S. V.; Pogodaeva, T. V.; Turchyn, A. V.; Khlystov, O.M.; Khabuev, A. V.; Ivanov, V.G.; Krylov, A.A.; Zemskaya, T.I. Microbial Diversity and Authigenic Siderite Mediation in Sediments Surrounding the Kedr-1 Mud Volcano, Lake Baikal. *Geobiology* **2023**, *21*, 770–790, https://doi.org/10.1111/gbi.12575.

221. Bashenkhaeva, M. V.; Zakharova, Y.R.; Petrova, D.P.; Khanaev, I. V.; Galachyants, Y.P.; Likhoshway, Y. V. Sub-Ice Microalgal and Bacterial Communities in Freshwater Lake Baikal, Russia. *Microb Ecol* **2015**, *70*, 751–765, https://doi.org/10.1007/s00248-015-0619-2.

222. Dmitrieva, M.E.; Malygina, E. V.; Belyshenko, A.Y.; Shelkovnikova, V.N.; Imidoeva, N.A.; Morgunova, M.M.; Telnova, T.Y.; Vlasova, A.A.; Axenov-Gribanov, D. V. The Effects of a High Concentration of Dissolved Oxygen on Actinobacteria from Lake Baikal. *Metabolites* **2023**, *13*, 830, https://doi.org/10.3390/metabo13070830.

223. Chernitsyna, S.M.; Khalzov, I.A.; Sitnikova, T.Ya.; Naumova, T. V.; Khabuev, A. V.; Zemskaya, T.I. Microbial Communities Associated with Bentic Invertebrates of Lake Baikal. *Curr Microbiol* **2021**, *78*, 3020–3031, https://doi.org/10.1007/s00284-021-02563-0.

224. Belykh, O.I.; Gladkikh, A.S.; Sorokovikova, E.G.; Tikhonova, I. V.; Butina, T. V. Identification of Toxic Cyanobacteria in Lake Baikal. *Dokl Biochem Biophys* **2015**, *463*, 220–224, https://doi.org/10.1134/S1607672915040067.

225. Galach'yants, A.D.; Bel'kova, N.L.; Sukhanova, E. V.; Romanovskaya, V.A.; Gladka, G. V.; Bedoshvili, E.D.; Parfenova, V. V. Diversity and Physiological and Biochemical Properties of Heterotrophic Bacteria Isolated from Lake Baikal Neuston. *Microbiology (N Y)* **2016**, *85*, 604–613, https://doi.org/10.1134/S0026261716050064.

226. Butina, T. V.; Petrushin, I.S.; Khanaev, I. V.; Bukin, Y.S. Metagenomic Assessment of DNA Viral Diversity in Freshwater Sponges, *Baikalospongia bacillifera*. *Microorganisms* **2022**, *10*, 480, https://doi.org/10.3390/microorganisms10020480.

227. Belykh, O.I.; Tikhonova, I. V.; Kuzmin, A. V.; Sorokovikova, E.G.; Fedorova, G.A.; Khanaev, I. V.; Sherbakova, T.A.; Timoshkin, O.A. First Detection of Benthic Cyanobacteria in Lake Baikal Producing Paralytic Shellfish Toxins. *Toxicon* **2016**, *121*, 36–40, https://doi.org/10.1016/j.toxicon.2016.08.015.

228. Bukin, S. V.; Pavlova, O.N.; Manakov, A.Y.; Kostyreva, E.A.; Chernitsyna, S.M.; Mamaeva, E. V.; Pogodaeva, T. V.; Zemskaya, T.I. The Ability of Microbial Community of Lake Baikal Bottom Sediments Associated with Gas Discharge to Carry Out the Transformation of Organic Matter under Thermobaric Conditions. *Front Microbiol* **2016**, *7*, https://doi.org/10.3389/fmicb.2016.00690.

229. Bondarenko, N.A.; Belykh, O.I.; Golobokova, L.P.; Artemyeva, O. V.; Logacheva, N.F.; Tikhonova, I. V.; Lipko, I.A.; Kostornova, T.Ya.; Parfenova, V. V.; Khodzher, T. V.; et al. Stratified Distribution of Nutrients and Extremophile Biota within Freshwater Ice Covering the Surface of Lake Baikal. *The Journal of Microbiology* **2012**, *50*, 8–16, https://doi.org/10.1007/s12275-012-1251-1.

230. Zemskaya, T.I.; Chernitsyna, S.M.; Dul'tseva, N.M.; Sergeeva, V.N.; Pogodaeva, T. V.; Namsaraev, B.B. Colorless Sulfur Bacteria *Thioploca* from Different Sites in Lake Baikal. *Microbiology (N Y)* **2009**, *78*, 117–124, https://doi.org/10.1134/S0026261709010159.

231. Sorokovikova, E.G.; Belykh, O.I.; Gladkikh, A.S.; Kotsar, O. V.; Tikhonova, I. V.; Timoshkin, O.A.; Parfenova, V. V. Diversity of Cyanobacterial Species and Phylotypes in Biofilms from the Littoral Zone of Lake Baikal. *Journal of Microbiology* **2013**, *51*, 757–765, https://doi.org/10.1007/s12275-013-3240-4.

232. Chernogor, L.; Klimenko, E.; Khanaev, I.; Belikov, S. Microbiome Analysis of Healthy and Diseased Sponges *Lubomirskia Baicalensis* by Using Cell Cultures of Primmorphs. *PeerJ* **2020**, *8*, e9080, https://doi.org/10.7717/peerj.9080.

233. Kulakova, N. V.; Sakirko, M. V.; Adelshin, R. V.; Khanaev, I. V.; Nebesnykh, I.A.; Pérez, T. Brown Rot Syndrome and Changes in the Bacterial Community of the Baikal Sponge *Lubomirskia baicalensis*. *Microb Ecol* **2018**, *75*, 1024–1034, https://doi.org/10.1007/s00248-017-1097-5.

234. Maksimov, V. V; Shchetinina, E. V; Kraĭkivskaia, O. V; Maksimov, V.N.; Maksimova, E.A. The Classification and the Monitoring of the State of Mouth Riverine and Lacustrine Ecosystems in Lake Baikal Based on the Composition of Local Microbiocenoses and Their Activity. *Microbiology* **2002**, *71*, 690–696, https://doi.org/10.1023/A:1020571122456.

235. Zakharenko, A.S.; Pimenov, N. V.; Ivanova, V.G.; Zemskaya, T.I. Detection of Methane in the Water Column at Gas and Oil Seep Sites in Central and Southern Lake Baikal. *Microbiology (N Y)* **2015**, *84*, 90–97, https://doi.org/10.1134/S0026261715010178.

236. Kozyreva, L.; Egorova, D.; Anan'ina, L.; Plotnikova, E.; Ariskina, E.; Prisyazhnaya, N.; Radnaeva, L.; Namsaraev, B. *Belliella buryatensis* sp. nov., Isolated from Alkaline Lake Water. *Int J Syst Evol Microbiol* **2016**, *66*, 137–143, https://doi.org/10.1099/ijsem.0.000682.

237. Bel'kova, N.L.; Driukker, V. V; Xong, S.K.; An, T.S. Study of the Aquatic Bacterial Community Composition of Baikal Lake by in *situ* Hybridization Assay. *Mikrobiologiia* **2003**, *72*, 282–283, https://doi.org/10.1023/A:1023288602726.

238. Pimenov, N. V.; Zakharova, E.E.; Bryukhanov, A.L.; Korneeva, V.A.; Kuznetsov, B.B.; Tourova, T.P.; Pogodaeva, T. V.; Kalmychkov, G. V.; Zemskaya, T.I. Activity and Structure of the Sulfate-Reducing Bacterial Community in the Sediments of the Southern Part of Lake Baikal. *Microbiology (N Y)* **2014**, *83*, 47–55, https://doi.org/10.1134/S0026261714020167.

239. Kalashnikov, A.M.; Gaisin, V.A.; Sukhacheva, M. V.; Namsaraev, B.B.; Panteleeva, A.N.; Nuyanzina-Boldareva, E.N.; Kuznetsov, B.B.; Gorlenko, V.M. Anoxygenic Phototrophic Bacteria from Microbial Communities of Goryachinsk Thermal Spring (Baikal Area, Russia). *Microbiology (N Y)* **2014**, *83*, 407–421, https://doi.org/10.1134/S0026261714040080.

240. Spiglazov, L.P.; Drucker, V. V; Ahn, T.S. Bacterial Aggregates Formation after Addition of Glucose in Lake Baikal Water. *J Microbiol* **2004**, *42*, 357–360.

241. Zelenkina, T.S.; Eshinimayev, B.Ts.; Dagurova, O.P.; Suzina, N.E.; Namsarayev, B.B.; Trotsenko, Yu.A. Aerobic Methanotrophs from the Coastal Thermal Springs of Lake Baikal. *Microbiology (N Y)* **2009**, *78*, 492–497, https://doi.org/10.1134/S0026261709040134.

242. Zakharyuk, A.G.; Kozyreva, L.P.; Khijniak, T. V.; Namsaraev, B.B.; Shcherbakova, V.A. *Desulfonatronum zhilinae* sp. nov., a Novel Haloalkaliphilic Sulfate-Reducing Bacterium from Soda Lake Alginskoe, Trans-Baikal Region, Russia. *Extremophiles* **2015**, *19*, 673–680, https://doi.org/10.1007/s00792-015-0747-0.

243. Garnova, E.S.; Zhilina, T.N.; Tourova, T.P.; Lysenko, A.M. *Anoxynatronum sibiricum* gen. nov., sp nov. Alkaliphilic Saccharolytic Anaerobe from Cellulolytic Community of Nizhnee Beloe (Transbaikal Region). *Extremophiles* **2003**, *7*, 213–220, https://doi.org/10.1007/s00792-002-0312-5.

244. Grachev, M.; Zubkov, I.; Tikhonova, I.; Ivacheva, M.; Kuzmin, A.; Sukhanova, E.; Sorokovikova, E.; Fedorova, G.; Galkin, A.; Suslova, M.; et al. Extensive Contamination of Water with Saxitoxin Near the Dam of the Irkutsk Hydropower Station Reservoir (East Siberia, Russia). *Toxins (Basel)* **2018**, *10*, 402, https://doi.org/10.3390/toxins10100402.

245. Jung, D.; Seo, E.-Y.; Epstein, S.S.; Joung, Y.; Han, J.; Parfenova, V. V.; Belykh, O.I.; Gladkikh, A.S.; Ahn, T.S. Application of a New Cultivation Technology, I-Tip, for Studying Microbial Diversity in Freshwater Sponges of Lake Baikal, Russia. *FEMS Microbiol Ecol* **2014**, n/a-n/a, https://doi.org/10.1111/1574-6941.12399.

246. Dul'tseva, N.M.; Chernitsina, S.M.; Zemskaya, T.I. Isolation of Bacteria of the Genus *Variovorax* from the *Thioploca* Mats of Lake Baikal. *Microbiology (N Y)* **2012**, *81*, 67–78, https://doi.org/10.1134/S0026261712010067.

247. Denisova, L.I.; Bel'kova, N.L.; Tulokhonov, I.I.; Zaĭchikov, E.F. Diversity of Bacteria at Various Depths in the Southern Part of Lake Baikal as Detected by 16S RRNA Sequencing. *Mikrobiologiia* **1999**, *68*, 547–556.

248. Pavlova, O.N.; Lomakina, A. V.; Gorshkov, A.G.; Suslova, M.Yu.; Likhoshvai, A. V.; Zemskaya, T.I. Microbial Communities and Their Ability to Oxidize N-Alkanes in the Area of Release of Gas- and Oil-Containing Fluids in Mid-Baikal (Cape Gorevoi Utes). *Biology Bulletin* **2012**, *39*, 458–463, https://doi.org/10.1134/S1062359012050123.

249. Gladkikh, A.S.; Kalyuzhnaya, Ok. V.; Belykh, O.I.; Ahn, T.S.; Parfenova, V. V. Analysis of Bacterial Communities of Two Lake Baikal Endemic Sponge Species. *Microbiology (N Y)* **2014**, *83*, 787–797, https://doi.org/10.1134/S002626171406006X.

250. Kovaleva, O.L.; Merkel, A.Yu.; Novikov, A.A.; Baslerov, R. V.; Toshchakov, S. V.; Bonch-Osmolovskaya, E.A. *Tepidisphaera mucosa* gen. nov., sp. nov., a Moderately Thermophilic Member of the Class *Phycisphaerae* in the Phylum *Planctomycetes*, and Proposal of a New Family, *Tepidisphaeraceae* fam. nov., and a New Order, *Tepidisphaerales* ord. nov. *Int J Syst Evol Microbiol* **2015**, *65*, 549–555, https://doi.org/10.1099/ijs.0.070151-0.

251. Chernitsyna, S.M.; Khal'zov, I.A.; Khanaeva, T.A.; Morozov, I. V.; Klimenkov, I. V.; Pimenov, N. V.; Zemskaya, T.I. Microbial Community Associated with *Thioploca* sp. Sheaths in the Area of the Posolsk Bank Methane Seep, Southern Baikal. *Microbiology (N Y)* **2016**, *85*, 562–569, https://doi.org/10.1134/S0026261716050027.

252. Pavlova, O.N.; Izosimova, O.N.; Chernitsyna, S.M.; Ivanov, V.G.; Pogodaeva, T. V.; Khabuev, A. V.; Gorshkov, A.G.; Zemskaya, T.I. Anaerobic Oxidation of Petroleum Hydrocarbons in Enrichment Cultures from Sediments of the Gorevoy Utes Natural Oil Seep under Methanogenic and Sulfate-Reducing Conditions. *Microb Ecol* **2022**, *83*, 899–915, https://doi.org/10.1007/s00248-021-01802-y.

253. Brown, I.I.; Galperin, M.Yu.; Glagolev, A.N.; Skulachev, V.P. Utilization of Energy Stored in the Form of Na^+ and K^+ Ion Gradients by Bacterial Cells. *Eur J Biochem* **1983**, *134*, 345–349, https://doi.org/10.1111/j.1432-1033.1983.tb07573.x.

254. Tulupova, Yu.R.; Parfenova, V. V.; Sitnikova, T.Ya.; Sorokovnikova, E.G.; Khanaev, I.B. First Report on Bacteria of the Family *Spirochaetaceae* from Digestive Tract of Endemic Gastropods from Lake Baikal. *Microbiology (N Y)* **2012**, *81*, 460–467, https://doi.org/10.1134/S0026261712030150.

255. Maksimenko, S.Yu.; Zemskaya, T.I.; Pavlova, O.N.; Ivanov, V.G.; Buryukhaev, S.P. Microbial Community of the Water Column of the Selenga River-Lake Baikal Biogeochemical Barrier. *Microbiology (N Y)* **2008**, *77*, 587–594, https://doi.org/10.1134/S0026261708050123.

256. Shubenkova, O. V.; Zemskaya, T.I.; Chernitsyna, S.M.; Khlystov, O.M.; Triboi, T.I. The First Results of an Investigation into the Phylogenetic Diversity of Microorganisms in Southern Baikal Sediments in the Region of Subsurface Discharge of Methane Hydrates. *Microbiology (N Y)* **2005**, *74*, 314–320, https://doi.org/10.1007/s11021-005-0069-9.

257. Maksimov, V. V.; Shchetinina, E. V.; Kraykivskaya, O. V.; Maksimova, E.A. Response of Microbial Communities of Lake Baikal to Extreme Temperatures. *Microbiology (N Y)* **2006**, *75*, 653–657, https://doi.org/10.1134/S0026261706060063.

258. Kaluzhnaya, O. V.; Itskovich, V.B. Phototrophic Microorganisms in the Symbiotic Communities of Baikal Sponges: Diversity of *psbA* Gene (Encoding D1 Protein of Photosystem II) Sequences. *Mol Biol* **2017**, *51*, 372–378, https://doi.org/10.1134/S0026893317030086.

259. Bel'kova, N.L.; Parfenova, V. V; Kostopnova, T.I.; Denisova, L.I.; Zaĭchikov, E.F. Microbial Biodiversity in the Lake Baikal Water. *Mikrobiologiia* **2003**, *72*, 239–249, https://doi.org/10.1023/A:1023224215929.

260. Gainutdinova, E.A.; Eshinimaev, B.Ts.; Tsyrenzhapova, I.S.; Dagurova, O.P.; Suzina, N.E.; Khmelenina, V.N.; Namsaraev, B.B.; Trotsenko, Yu.A. Aerobic Methanotrophic Communities in the Bottom Sediments of Lake Baikal. *Microbiology (N Y)* **2005**, *74*, 486–494, https://doi.org/10.1007/s11021-005-0093-9.

261. Belykh, O.I.; Glagkikh, A.S.; Tikhonova, I. V.; Kuz'min, A. V.; Mogil'nikova, T.A.; Fedorova, G.A.; Sorokovikova, E.G. Identification of Cyanobacterial Producers of Shellfish Paralytic Toxins in Lake Baikal and Reservoirs of the Angara River. *Microbiology (N Y)* **2015**, *84*, 98–99, https://doi.org/10.1134/S0026261715010038.

262. Parfenova, V. V; Kravchenko, O.S.; Pavlova, O.N.; Suslova, M.I.; Bedoshvili, E.D. [Effect of Different Calcium Hypochlorite Concentrations on the Survival of Potentially Pathogenic Microorganisms Isolated from Baikal Lake]. *Gig Sanit* **2012**, 8–12.

263. Semenova, E.A.; Kuznedelov, K.D.; Grachev, M.A. Nucleotide Sequences of Fragments of 16S RRNA of the Baikal Natural Populations and Laboratory Cultures of Cyanobacteria. *Mol Biol* **2001**, *35*, 405–410, https://doi.org/10.1023/A:1010482930472.

264. Terkina, I.A.; Drukker, V. V.; Parfenova, V. V.; Kostornova, T.Ya. The Biodiversity of Actinomycetes in Lake Baikal. *Microbiology (N Y)* **2002**, *71*, 346–349, https://doi.org/10.1023/A:1015871115187.

265. Lavrenteva, E. V.; Shagzhina, A.P.; Babasanova, O.B.; Dunaevsky, Y.E.; Namsaraev, Z.B.; Barkhutova, D.D. The Study of Two Alkaliphilic Thermophile Bacteria of the *Anoxybacillus* Genus as Producers of Extracellular Proteinase. *Appl Biochem Microbiol* **2009**, *45*, 484–488, https://doi.org/10.1134/S0003683809050044.

266. Andreeva, I.S.; Pechurkina, N.I.; Morozova, O. V.; Ryabchikova, E.I.; Belikov, S.I.; Puchkova, L.I.; Emel'yanova, E.K.; Torok, T.; Repin, V.E. The New Eubacterium *Roseomonas baikalica* sp. nov. Isolated from Core Samples Collected by Deep-Hole Drilling of the Bottom of Lake Baikal. *Microbiology (N Y)* **2007**, *76*, 487–493, https://doi.org/10.1134/S0026261707040157.

267. Sorokovikova, E.G.; Tikhonova, I. V.; Belykh, O.I.; Klimenkov, I. V.; Likhoshwai, E. V. Identification of Two Cyanobacterial Strains Isolated from the Kotel'nikovskii Hot Spring of the Baikal Rift. *Microbiology (N Y)* **2008**, *77*, 365–372, https://doi.org/10.1134/S002626170803017X.

268. Li, B.; Li, Q.; Xu, Z.; Zhang, N.; Shen, Q.; Zhang, R. Responses of Beneficial *Bacillus amyloliquefaciens* SQR9 to Different Soilborne Fungal Pathogens through the Alteration of Antifungal Compounds Production. *Front Microbiol* **2014**, *5*, https://doi.org/10.3389/fmicb.2014.00636.

269. Mácha, H.; Marešová, H.; Juříková, T.; Švecová, M.; Benada, O.; Škríba, A.; Baránek, M.; Novotný, Č.; Palyzová, A. Killing Effect of *Bacillus velezensis* Fzb42 on a (*Xcc*) Strain Newly Isolated from Cabbage *Brassica oleracea* convar. *capitata* (L.): A Metabolomic Study. *Microorganisms* **2021**, *9*, https://doi.org/10.3390/microorganisms9071410.

270. Pengproh, R.; Thanyasiriwat, T.; Sangdee, K.; Saengprajak, J.; Kawicha, P.; Sangdee, A. Evaluation and Genome Mining of *Bacillus stercoris* Isolate B.PNR1 as Potential Agent for *Fusarium* Wilt Control and Growth Promotion of Tomato. *Plant Pathol J* **2023**, *39*, 430–448, https://doi.org/10.5423/PPJ.OA.01.2023.0018.

271. Nifakos, K.; Tsalgatidou, P.C.; Thomloudi, E.-E.; Skagia, A.; Kotopoulis, D.; Baira, E.; Delis, C.; Papadimitriou, K.; Markellou, E.; Venieraki, A.; et al. Genomic Analysis and Secondary Metabolites Production of the Endophytic *Bacillus velezensis* Bvel1: A Biocontrol Agent against *Botrytis cinerea* Causing Bunch Rot in Post-Harvest Table Grapes. *Plants* **2021**, *10*, 1716, https://doi.org/10.3390/plants10081716.

272. Fatima, R.; Mahmood, T.; Moosa, A.; Aslam, M.N.; Shakeel, M.T.; Maqsood, A.; Shafiq, M.U.; Ahmad, T.; Moustafa, M.; Al-Shehri, M. *Bacillus thuringiensis* CHGP12 Uses a Multifaceted Approach for the Suppression of *Fusarium oxysporum* f. sp. *siceris* and to Enhance the Biomass of Chickpea Plants. *Pest Manag Sci* **2023**, *79*, 336–348, https://doi.org/10.1002/ps.7203.

273. Doty, S.L.; Joubert, P.M.; Firrincieli, A.; Sher, A.W.; Tournay, R.; Kill, C.; Parikh, S.S.; Okubara, P. Potential Biocontrol Activities of Populus Endophytes against Several Plant Pathogens Using Different Inhibitory Mechanisms. *Pathogens* **2022**, *12*, 13, https://doi.org/10.3390/pathogens12010013.

274. Wang, K.-X.; Xu, W.-H.; Chen, Z.-N.; Hu, J.-L.; Luo, S.-Q.; Wang, Z.-G. Complete Genome Sequence of *Bacillus velezensis* WB, an Isolate from the Watermelon Rhizosphere: Genomic Insights into Its Antifungal Effects. *J Glob Antimicrob Resist* **2022**, *30*, 442–444, https://doi.org/10.1016/j.jgar.2022.05.010.

275. R, S.; Nakkeeran, S.; Saranya, N.; Senthilraja, C.; Renukadevi, P.; Krishnamoorthy, A.S.; El Enshasy, H.A.; El-Adawi, H.; Malathi, V.G.; Salmen, S.H.; et al. Mining the Genome of *Bacillus velezensis* VB7 (CP047587) for MAMP Genes and Non-Ribosomal Peptide Synthetase Gene Clusters Conferring Antiviral and Antifungal Activity. *Microorganisms* **2021**, *9*, 2511, https://doi.org/10.3390/microorganisms9122511.

276. Heo, Y.; Lee, Y.; Balaraju, K.; Jeon, Y. Characterization and Evaluation of *Bacillus subtilis* GYUN-2311 as a Biocontrol Agent against *Colletotrichum* spp. on Apple and Hot Pepper in Korea. *Front Microbiol* **2024**, *14*, https://doi.org/10.3389/fmicb.2023.1322641.

277. Tsalgatidou, P.C.; Thomloudi, E.-E.; Baira, E.; Papadimitriou, K.; Skagia, A.; Venieraki, A.; Katinakis, P. Integrated Genomic and Metabolomic Analysis Illuminates Key Secreted Metabolites Produced by the Novel Endophyte *Bacillus halotolerans* Cal.l.30 Involved in Diverse Biological Control Activities. *Microorganisms* **2022**, *10*, 399, https://doi.org/10.3390/microorganisms10020399.

278. Zhou, L.; Song, C.; Li, Z.; Kuipers, O.P. Antimicrobial Activity Screening of Rhizosphere Soil Bacteria from Tomato and Genome-Based Analysis of Their Antimicrobial Biosynthetic Potential. *BMC Genomics* **2021**, *22*, 29, https://doi.org/10.1186/s12864-020-07346-8.

279. Kamali, M.; Guo, D.; Naeimi, S.; Ahmadi, J. Perception of Biocontrol Potential of *Bacillus inaquosorum* KR2-7 against Tomato Fusarium Wilt through Merging Genome Mining with Chemical Analysis. *Biology (Basel)* **2022**, *11*, 137, https://doi.org/10.3390/biology11010137.

280. Villa-Rodriguez, E.; Moreno-Ulloa, A.; Castro-Longoria, E.; Parra-Cota, F.I.; de los Santos-Villalobos, S. Integrated Omics Approaches for Deciphering Antifungal Metabolites Produced by a Novel *Bacillus* Species, *B. cabrialesii* TE3^T, against the Spot Blotch Disease of Wheat (*Triticum turgidum* L. subsp. *durum*). *Microbiol Res* **2021**, *251*, 126826, https://doi.org/10.1016/j.micres.2021.126826.

281. Nimbeshaho, F.; Nihorimbere, G.; Arias, A.A.; Liénard, C.; Steels, S.; Nibasumba, A.; Nihorimbere, V.; Legrève, A.; Ongena, M. Unravelling the Secondary Metabolome and Biocontrol Potential of the Recently Described Species *Bacillus nakamurai*. *Microbiol Res* **2024**, *288*, 127841, https://doi.org/10.1016/j.micres.2024.127841.
282. Liu, H.; Yin, S.; An, L.; Zhang, G.; Cheng, H.; Xi, Y.; Cui, G.; Zhang, F.; Zhang, L. Complete Genome Sequence of *Bacillus subtilis* BSD-2, a Microbial Germicide Isolated from Cultivated Cotton. *J Biotechnol* **2016**, *230*, 26–27, https://doi.org/10.1016/j.jbiotec.2016.05.019.
283. Li, S.; He, P.; Fan, H.; Liu, L.; Yin, K.; Yang, B.; Li, Y.; Huang, S.-M.; Li, X.; Zheng, S.-J. A Real-Time Fluorescent Reverse Transcription Quantitative PCR Assay for Rapid Detection of Genetic Markers' Expression Associated with Fusarium Wilt of Banana Biocontrol Activities in *Bacillus*. *Journal of Fungi* **2021**, *7*, 353, https://doi.org/10.3390/jof7050353.
284. Wang, Y.; Sun, Z.; Zhao, Q.; Yang, X.; Li, Y.; Zhou, H.; Zhao, M.; Zheng, H. Whole-Genome Analysis Revealed the Growth-Promoting and Biological Control Mechanism of the Endophytic Bacterial Strain *Bacillus halotolerans* Q2H2, with Strong Antagonistic Activity in Potato Plants. *Front Microbiol* **2024**, *14*, https://doi.org/10.3389/fmicb.2023.1287921.
285. Ferreira, W.T.; Hong, H.A.; Hess, M.; Adams, J.R.G.; Wood, H.; Bakun, K.; Tan, S.; Baccigalupi, L.; Ferrari, E.; Brisson, A.; et al. Micellar Antibiotics of *Bacillus*. *Pharmaceutics* **2021**, *13*, 1296, https://doi.org/10.3390/pharmaceutics13081296.
286. Xue, J.; Sun, L.; Xu, H.; Gu, Y.; Lei, P. *Bacillus atrophaeus* NX-12 Utilizes Exosmotic Glycerol from *Fusarium oxysporum* f. sp. *cucumerinum* for Fengycin Production. *J Agric Food Chem* **2023**, *71*, 10565–10574, https://doi.org/10.1021/acs.jafc.3c01276.
287. Santos, J.B. dos; Cruz, J. de O.; Geraldo, L.C.; Dias, E.G.; Queiroz, P.R.M.; Monnerat, R.G.; Borges, M.; Blassioli-Moraes, M.C.; Blum, L.E.B. Detection and Evaluation of Volatile and Non-Volatile Antifungal Compounds Produced by *Bacillus* spp. Strains. *Microbiol Res* **2023**, *275*, 127465, https://doi.org/10.1016/j.micres.2023.127465.
288. Adeniji, A.A.; Aremu, O.S.; Babalola, O.O. Selecting Lipopeptide-producing, *Fusarium*-Suppressing *Bacillus* spp.: Metabolomic and Genomic Probing of *Bacillus velezensis* NWUMFkBS10.5. *Microbiologyopen* **2019**, *8*, https://doi.org/10.1002/mbo3.742.
289. Jiao, R.; Cai, Y.; He, P.; Munir, S.; Li, X.; Wu, Y.; Wang, J.; Xia, M.; He, P.; Wang, G.; et al. *Bacillus amyloliquefaciens* YN201732 Produces Lipopeptides With Promising Biocontrol Activity Against Fungal Pathogen *Erysiphe cichoracearum*. *Front Cell Infect Microbiol* **2021**, *11*, https://doi.org/10.3389/fcimb.2021.598999.
290. Li, S.; Xu, J.; Fu, L.; Xu, G.; Lin, X.; Qiao, J.; Xia, Y. Biocontrol of Wheat Crown Rot Using *Bacillus halotolerans* QTH8. *Pathogens* **2022**, *11*, 595, https://doi.org/10.3390/pathogens11050595.
291. Hammad, M.; Ali, H.; Hassan, N.; Tawab, A.; Salman, M.; Jawad, I.; de Jong, A.; Moreno, C.M.; Kuipers, O.P.; Feroz, Y.; et al. Food Safety and Biological Control; Genomic Insights and Antimicrobial Potential of *Bacillus velezensis* FB2 against Agricultural Fungal Pathogens. *PLoS One* **2023**, *18*, e0291975, https://doi.org/10.1371/journal.pone.0291975.
292. Lu, H.; Yang, P.; Zhong, M.; Bilal, M.; Xu, H.; Zhang, Q.; Xu, J.; Liang, N.; Liu, S.; Zhao, L.; et al. Isolation of a Potential Probiotic Strain *Bacillus amyloliquefaciens* LPB-18 and Identification of Antimicrobial Compounds Responsible for Inhibition of Food-borne Pathogens. *Food Sci Nutr* **2023**, *11*, 2186–2196, https://doi.org/10.1002/fsn3.3094.
293. Santos-Lima, D.; de Castro Spadari, C.; de Morais Barroso, V.; Carvalho, J.C.S.; de Almeida, L.C.; Alcalde, F.S.C.; Ferreira, M.J.P.; Sannomiya, M.; Ishida, K. Lipopeptides from an Isolate of *Bacillus Subtilis* Complex Have Inhibitory and Antibiofilm Effects on *Fusarium Solani*. *Appl Microbiol Biotechnol* **2023**, *107*, 6103–6120, https://doi.org/10.1007/s00253-023-12712-z.
294. Kim, Y.T.; Kim, S.E.; Lee, W.J.; Fumei, Z.; Cho, M.S.; Moon, J.S.; Oh, H.-W.; Park, H.-Y.; Kim, S.U. Isolation and Characterization of a High Iturin Yielding *Bacillus velezensis* UV Mutant with Improved Antifungal Activity. *PLoS One* **2020**, *15*, e0234177, https://doi.org/10.1371/journal.pone.0234177.
295. Afordoanyi, D.M.; Diabankana, R.G.C.; Komissarov, E.N.; Kuchaev, E.S.; Validov, S.Z. Characterization of a Novel *Bacillus glycinifermentans* Strain MGMM1 Based on Full Genome Analysis and Phenotypic Properties for Biotechnological Applications. *Microorganisms* **2023**, *11*, 1410, https://doi.org/10.3390/microorganisms11061410.
296. Zhao, P.; Quan, C.; Wang, Y.; Wang, J.; Fan, S. *Bacillus amyloliquefaciens* Q-426 as a Potential Biocontrol Agent against *Fusarium oxysporum* f. sp. *spinaciae*. *J Basic Microbiol* **2014**, *54*, 448–456, https://doi.org/10.1002/jobm.201200414.
297. Vignesh, M.; Shankar, S.R.M.; MubarakAli, D.; Hari, B.N.V. A Novel Rhizospheric Bacterium: *Bacillus velezensis* NKMV-3 as a Biocontrol Agent Against Alternaria Leaf Blight in Tomato. *Appl Biochem Biotechnol* **2022**, *194*, 1–17, https://doi.org/10.1007/s12010-021-03684-9.
298. Zhou, L.; Song, C.; Muñoz, C.Y.; Kuipers, O.P. *Bacillus cabrialesii* BH5 Protects Tomato Plants Against *Botrytis cinerea* by Production of Specific Antifungal Compounds. *Front Microbiol* **2021**, *12*, https://doi.org/10.3389/fmicb.2021.707609.
299. Cao, Y.; Pi, H.; Chandrangsu, P.; Li, Y.; Wang, Y.; Zhou, H.; Xiong, H.; Helmann, J.D.; Cai, Y. Antagonism of Two Plant-Growth Promoting *Bacillus selezensis* Isolates Against *Ralstonia solanacearum* and *Fusarium sxysporum*. *Sci Rep* **2018**, *8*, 4360, https://doi.org/10.1038/s41598-018-22782-z.
300. Deng, Y.; Chen, Z.; Chen, Y.; Wang, J.; Xiao, R.; Wang, X.; Liu, B.; Chen, M.; He, J. Lipopeptide C $_{17}$ Fengycin B Exhibits a Novel Antifungal Mechanism by Triggering Metacaspase-Dependent Apoptosis in *Fusarium oxysporum*. *J Agric Food Chem* **2024**, *72*, 7943–7953, https://doi.org/10.1021/acs.jafc.4c00126.
301. Wang, K.; Wang, Z.; Xu, W. Induced Oxidative Equilibrium Damage and Reduced Toxin Synthesis in *Fusarium oxysporum* f. sp . *niveum* by Secondary Metabolites from *Bacillus velezensis* WB. *FEMS Microbiol Ecol* **2022**, *98*, https://doi.org/10.1093/femsec/fiac080.
302. Ali, S.; Hameed, S.; Shahid, M.; Iqbal, M.; Lazarovits, G.; Imran, A. Functional Characterization of Potential PGPR Exhibiting Broad-Spectrum Antifungal Activity. *Microbiol Res* **2020**, *232*, 126389, https://doi.org/10.1016/j.micres.2019.126389.
303. Jan, F.; Arshad, H.; Ahad, M.; Jamal, A.; Smith, D.L. *In vitro* Assessment of *Bacillus subtilis* FJ3 Affirms Its Biocontrol and Plant Growth Promoting Potential. *Front Plant Sci* **2023**, *14*, https://doi.org/10.3389/fpls.2023.1205894.

304. Al-Mutar, D.M.K.; Alzawar, N.S.A.; Noman, M.; Azizullah; Li, D.; Song, F. Suppression of *Fusarium* Wilt in Watermelon by *Bacillus amyloliquefaciens* DHA55 through Extracellular Production of Antifungal Lipopeptides. *Journal of Fungi* **2023**, *9*, 336, https://doi.org/10.3390/jof9030336.

305. Li, Q.; Liao, S.; Zhi, H.; Xing, D.; Xiao, Y.; Yang, Q. Characterization and Sequence Analysis of Potential Biofertilizer and Biocontrol Agent *Bacillus subtilis* Strain SEM-9 from Silkworm Excrement. *Can J Microbiol* **2019**, *65*, 45–58, https://doi.org/10.1139/cjm-2018-0350.

306. Wang, J.; Qiu, J.; Yang, X.; Yang, J.; Zhao, S.; Zhou, Q.; Chen, L. Identification of Lipopeptide Iturin A Produced by *Bacillus amyloliquefaciens* NCPSJ7 and Its Antifungal Activities against *Fusarium oxysporum* f. sp. *siveum*. *Foods* **2022**, *11*, 2996, https://doi.org/10.3390/foods11192996.

307. Berlanga-Clavero, M. V.; Molina-Santiago, C.; Caraballo-Rodríguez, A.M.; Petras, D.; Díaz-Martínez, L.; Pérez-García, A.; de Vicente, A.; Carrión, V.J.; Dorrestein, P.C.; Romero, D. *Bacillus subtilis* Biofilm Matrix Components Target Seed Oil Bodies to Promote Growth and Anti-Fungal Resistance in Melon. *Nat Microbiol* **2022**, *7*, 1001–1015, https://doi.org/10.1038/s41564-022-01134-8.

308. Ghazala, I.; Charfeddine, S.; Charfeddine, M.; Gargouri-Bouzid, R.; Ellouz-Chaabouni, S.; Haddar, A. Antimicrobial and Antioxidant Activities of *Bacillus mojavensis* I4 Lipopeptides and Their Potential Application against the Potato Dry Rot Causative *Fusarium solani*. *Arch Microbiol* **2022**, *204*, 484, https://doi.org/10.1007/s00203-022-03098-z.

309. Samaras, A.; Nikolaidis, M.; Antequera-Gómez, M.L.; Cámara-Almirón, J.; Romero, D.; Moschakis, T.; Amoutzias, G.D.; Karaoglanidis, G.S. Whole Genome Sequencing and Root Colonization Studies Reveal Novel Insights in the Biocontrol Potential and Growth Promotion by *Bacillus subtilis* MBI 600 on Cucumber. *Front Microbiol* **2021**, *11*, https://doi.org/10.3389/fmicb.2020.600393.

310. Wang, C.; Ye, X.; Ng, T.B.; Zhang, W. Study on the Biocontrol Potential of Antifungal Peptides Produced by *Bacillus velezensis* against *Fusarium solani* That Infects the Passion Fruit *Passiflora edulis*. *J Agric Food Chem* **2021**, *69*, 2051–2061, https://doi.org/10.1021/acs.jafc.0c06106.

311. Guevara-Avendaño, E.; Lizette Pérez-Molina, M.; Luis Monribot-Villanueva, J.; Marian Cortazar-Murillo, E.; Ramírez-Vázquez, M.; Reverchon, F.; Antonio Guerrero-Analco, J. Identification of Antifungal Compounds from Avocado Rhizobacteria (*Bacillus* spp.) against *Fusarium* spp., by a Bioassay-Guided Fractionation Approach. *Chem Biodivers* **2022**, *19*, https://doi.org/10.1002/cbdv.202200687.

312. Al-Mutar, D.M.K.; Noman, M.; Alzawar, N.S.A.; Qasim, H.H.; Li, D.; Song, F. The Extracellular Lipopeptides and Volatile Organic Compounds of *Bacillus subtilis* DHA41 Display Broad-Spectrum Antifungal Activity against Soil-Borne Phytopathogenic Fungi. *Journal of Fungi* **2023**, *9*, 797, https://doi.org/10.3390/jof9080797.

313. Yang, D.; Zhang, X.; Li, Z.; Chu, R.; Shah, S.; Wang, X.; Zhang, X. Antagonistic Effect of *Bacillus* and *Pseudomonas* Combinations against *Fusarium oxysporum* and Their Effect on Disease Resistance and Growth Promotion in Watermelon. *J Appl Microbiol* **2024**, *135*, https://doi.org/10.1093/jambio/lxae074.

314. Dimkić, I.; Stanković, S.; Nišavić, M.; Petković, M.; Ristivojević, P.; Fira, D.; Berić, T. The Profile and Antimicrobial Activity of *Bacillus* Lipopeptide Extracts of Five Potential Biocontrol Strains. *Front Microbiol* **2017**, *8*, https://doi.org/10.3389/fmicb.2017.00925.

315. Báez-Vallejo, N.; Camarena-Pozos, D.A.; Monribot-Villanueva, J.L.; Ramírez-Vázquez, M.; Carrión-Villarnovo, G.L.; Guerrero-Analco, J.A.; Partida-Martínez, L.P.; Reverchon, F. Forest Tree Associated Bacteria for Potential Biological Control of *Fusarium solani* and of *Fusarium kuroshium*, Causal Agent of *Fusarium* Dieback. *Microbiol Res* **2020**, *235*, 126440, https://doi.org/10.1016/j.micres.2020.126440.

316. Moreno-Velandia, C.A.; Ongena, M.; Kloepper, J.W.; Cotes, A.M. Biosynthesis of Cyclic Lipopeptides by *Bacillus velezensis* Bs006 and Its Antagonistic Activity Are Modulated by the Temperature and Culture Media Conditions. *Curr Microbiol* **2021**, *78*, 3505–3515, https://doi.org/10.1007/s00284-021-02612-8.

317. Izquierdo-García, L.F.; González-Almario, A.; Cotes, A.M.; Moreno-Velandia, C.A. *Trichoderma virens* Gl006 and *Bacillus velezensis* Bs006: A Compatible Interaction Controlling *Fusarium* Wilt of Cape Gooseberry. *Sci Rep* **2020**, *10*, 6857, https://doi.org/10.1038/s41598-020-63689-y.

318. Hu, J.; Wang, Z.; Xu, W. Production-optimized Fermentation of Antifungal Compounds by *Bacillus velezensis* LZN01 and Transcriptome Analysis. *Microb Biotechnol* **2024**, *17*, https://doi.org/10.1111/1751-7915.70026.

319. Bóka, B.; Manczinger, L.; Kocsubé, S.; Shine, K.; Alharbi, N.S.; Khaled, J.M.; Münsterkötter, M.; Vágvölgyi, C.; Kredics, L. Genome Analysis of a Bacillus *subtilis* Strain Reveals Genetic Mutations Determining Biocontrol Properties. *World J Microbiol Biotechnol* **2019**, *35*, 52, https://doi.org/10.1007/s11274-019-2625-x.

320. Velho, R. V.; Medina, L.F.C.; Segalin, J.; Brandelli, A. Production of Lipopeptides among *Bacillus* Strains Showing Growth Inhibition of Phytopathogenic Fungi. *Folia Microbiol (Praha)* **2011**, *56*, 297–303, https://doi.org/10.1007/s12223-011-0056-7.

321. Li, X.; Zhang, Y.; Wei, Z.; Guan, Z.; Cai, Y.; Liao, X. Antifungal Activity of Isolated *Bacillus amyloliquefaciens* SYBC H47 for the Biocontrol of Peach Gummosis. *PLoS One* **2016**, *11*, e0162125, https://doi.org/10.1371/journal.pone.0162125.

322. Assena, M.W.; Pfannstiel, J.; Rasche, F. Inhibitory Activity of Bacterial Lipopeptides against *Fusarium oxysporum* f. sp. *strigae*. *BMC Microbiol* **2024**, *24*, 227, https://doi.org/10.1186/s12866-024-03386-2.

323. Chen, M.; Wang, J.; Liu, B.; Zhu, Y.; Xiao, R.; Yang, W.; Ge, C.; Chen, Z. Biocontrol of Tomato Bacterial Wilt by the New Strain *Bacillus velezensis* FJAT-46737 and Its Lipopeptides. *BMC Microbiol* **2020**, *20*, 160, https://doi.org/10.1186/s12866-020-01851-2.

324. Liu, H.; Wang, Y.; Yang, Q.; Zhao, W.; Cui, L.; Wang, B.; Zhang, L.; Cheng, H.; Song, S.; Zhang, L. Genomics and LC-MS Reveal Diverse Active Secondary Metabolites in *Bacillus amyloliquefaciens* WS-8. *J Microbiol Biotechnol* **2020**, *30*, 417–426, https://doi.org/10.4014/jmb.1906.06055.

325. Noh, J.S.; Hwang, S.H.; Maung, C.E.H.; Cho, J.-Y.; Kim, K.Y. Enhanced Control Efficacy of *Bacillus subtilis* NM4 via Integration of Chlorothalonil on Potato Early Blight Caused by *Alternaria solani*. *Microb Pathog* **2024**, *190*, 106604, https://doi.org/10.1016/j.micpath.2024.106604.

326. Nanjundan, J.; Ramasamy, R.; Uthandi, S.; Ponnusamy, M. Antimicrobial Activity and Spectroscopic Characterization of Surfactin Class of Lipopeptides from *Bacillus amyloliquefaciens* SR1. *Microb Pathog* **2019**, *128*, 374–380, https://doi.org/10.1016/j.micpath.2019.01.037.

327. Yang, P.; Zeng, Q.; Jiang, W.; Wang, L.; Zhang, J.; Wang, Z.; Wang, Q.; Li, Y. Genome Sequencing and Characterization of *Bacillus velezensis* N23 as Biocontrol Agent against Plant Pathogens. *Microorganisms* **2024**, *12*, 294, https://doi.org/10.3390/microorganisms12020294.

328. Ananev, A.A.; Ogneva, Z. V.; Nityagovsky, N.N.; Suprun, A.R.; Kiselev, K. V.; Aleynova, O.A. Whole Genome Sequencing of *Bacillus velezensis* AMR25, an Effective Antagonist Strain against Plant Pathogens. *Microorganisms* **2024**, *12*, 1533, https://doi.org/10.3390/microorganisms12081533.

329. Li, X.-Y.; Yang, J.-J.; Mao, Z.-C.; Ho, H.-H.; Wu, Y.-X.; He, Y.-Q. Enhancement of Biocontrol Activities and Cyclic Lipopeptides Production by Chemical Mutagenesis of *Bacillus subtilis* XF-1, a Biocontrol Agent of *Plasmodiophora brassicae* and *Fusarium solani*. *Indian J Microbiol* **2014**, *54*, 476–479, https://doi.org/10.1007/s12088-014-0471-y.

330. Toral, L.; Rodríguez, M.; Béjar, V.; Sampedro, I. Antifungal Activity of Lipopeptides From *Bacillus* XT1 CECT 8661 Against *Botrytis cinerea*. *Front Microbiol* **2018**, *9*, https://doi.org/10.3389/fmicb.2018.01315.

331. Jemil, N.; Besbes, I.; Gharbi, Y.; Triki, M.A.; Cheffi, M.; Manresa, A.; Nasri, M.; Hmidet, N. *Bacillus methylotrophicus* DCS1: Production of Different Lipopeptide Families, *in vitro* Antifungal Activity and Suppression of *Fusarium* Wilt in Tomato Plants. *Curr Microbiol* **2024**, *81*, 142, https://doi.org/10.1007/s00284-024-03660-6.

332. Yang, R.; Liu, P.; Ye, W.; Chen, Y.; Wei, D.; Qiao, C.; Zhou, B.; Xiao, J. Biological Control of Root Rot of Strawberry by *Bacillus amyloliquefaciens* Strains CMS5 and CMR12. *Journal of Fungi* **2024**, *10*, 410, https://doi.org/10.3390/jof10060410.

333. Yuan, J.; Raza, W.; Huang, Q.; Shen, Q. The Ultrasound-assisted Extraction and Identification of Antifungal Substances from *B. amyloliquefaciens* Strain NJN-6 Suppressing *Fusarium oxysporum*. *J Basic Microbiol* **2012**, *52*, 721–730, https://doi.org/10.1002/jobm.201100560.

334. Yaseen, Y.; Diop, A.; Gancel, F.; Béchet, M.; Jacques, P.; Drider, D. Polynucleotide Phosphorylase Is Involved in the Control of Lipopeptide Fengycin Production in *Bacillus subtilis*. *Arch Microbiol* **2018**, *200*, 783–791, https://doi.org/10.1007/s00203-018-1483-5.

335. Li, L.; Ma, M.; Huang, R.; Qu, Q.; Li, G.; Zhou, J.; Zhang, K.; Lu, K.; Niu, X.; Luo, J. Induction of Chlamydospore Formation in Fusarium by Cyclic Lipopeptide Antibiotics from *Bacillus subtilis* C2. *J Chem Ecol* **2012**, *38*, 966–974, https://doi.org/10.1007/s10886-012-0171-1.

336. Kobayashi, K. Plant Methyl Salicylate Induces Defense Responses in the Rhizobacterium *Bacillus subtilis*. *Environ Microbiol* **2015**, *17*, 1365–1376, https://doi.org/10.1111/1462-2920.12613.

337. Cazorla, F.M.; Romero, D.; Pérez-García, A.; Lugtenberg, B.J.J.; Vicente, A. de; Bloemberg, G. Isolation and Characterization of Antagonistic *Bacillus subtilis* Strains from the Avocado Rhizoplane Displaying Biocontrol Activity. *J Appl Microbiol* **2007**, *103*, 1950–1959, https://doi.org/10.1111/j.1365-2672.2007.03433.x.

338. Zhang, X.; Xin, Y.; Wang, J.; Dhanasekaran, S.; Yue, Q.; Feng, F.; Gu, X.; Li, B.; Zhao, L.; Zhang, H. Characterization of a *Bacillus velezensis* Strain as a Potential Biocontrol Agent against Soft Rot of Eggplant Fruits. *Int J Food Microbiol* **2024**, *410*, 110480, https://doi.org/10.1016/j.ijfoodmicro.2023.110480.

339. Rocha, G.T.; Queiroz, P.R.M.; Grynberg, P.; Togawa, R.C.; de Lima Ferreira, A.D.C.; do Nascimento, I.N.; Gomes, A.C.M.M.; Monnerat, R. Biocontrol Potential of Bacteria Belonging to the *Bacillus subtilis* Group against Pests and Diseases of Agricultural Interest through Genome Exploration. *Antonie Van Leeuwenhoek* **2023**, *116*, 599–614, https://doi.org/10.1007/s10482-023-01822-3.

340. Zhang, Q.X.; Zhang, Y.; He, L.L.; Ji, Z.L.; Tong, Y.H. Identification of a Small Antimycotic Peptide Produced by *Bacillus amyloliquefaciens* 6256. *Pestic Biochem Physiol* **2018**, *150*, 78–82, https://doi.org/10.1016/j.pestbp.2018.07.006.

341. Benitez, L.B.; Velho, R.V.; Lisboa, M.P.; da Costa Medina, L.F.; Brandelli, A. Isolation and Characterization of Antifungal Peptides Produced by *Bacillus amyloliquefaciens* LBM5006. *The Journal of Microbiology* **2010**, *48*, 791–797, https://doi.org/10.1007/s12275-010-0164-0.

342. Kefi, A.; Slimene, I. Ben; Karkouch, I.; Rihouey, C.; Azaeiz, S.; Bejaoui, M.; Belaid, R.; Cosette, P.; Jouenne, T.; Limam, F. Characterization of Endophytic *Bacillus* Strains from Tomato Plants (*Lycopersicon esculentum*) Displaying Antifungal Activity against *Botrytis cinerea* Pers. *World J Microbiol Biotechnol* **2015**, *31*, 1967–1976, https://doi.org/10.1007/s11274-015-1943-x.

343. Zeriouh, H.; Romero, D.; García-Gutiérrez, L.; Cazorla, F.M.; de Vicente, A.; Pérez-García, A. The Iturin-like Lipopeptides Are Essential Components in the Biological Control Arsenal of *Bacillus Subtilis* Against Bacterial Diseases of Cucurbits. *Molecular Plant-Microbe Interactions®* **2011**, *24*, 1540–1552, https://doi.org/10.1094/MPMI-06-11-0162.

344. Wang, P.; Guo, Q.; Ma, Y.; Li, S.; Lu, X.; Zhang, X.; Ma, P. DegQ Regulates the Production of Fengycins and Biofilm Formation of the Biocontrol Agent *Bacillus subtilis* NCD-2. *Microbiol Res* **2015**, *178*, 42–50, https://doi.org/10.1016/j.micres.2015.06.006.

345. Xu, Z.; Shao, J.; Li, B.; Yan, X.; Shen, Q.; Zhang, R. Contribution of Bacillomycin D in *Bacillus amyloliquefaciens* SQR9 to Antifungal Activity and Biofilm Formation. *Appl Environ Microbiol* **2013**, *79*, 808–815, https://doi.org/10.1128/AEM.02645-12.

346. Wu, G.; Liu, Y.; Xu, Y.; Zhang, G.; Shen, Q.; Zhang, R. Exploring Elicitors of the Beneficial Rhizobacterium *Bacillus amyloliquefaciens* SQR9 to Induce Plant Systemic Resistance and Their Interactions With Plant Signaling Pathways. *Molecular Plant-Microbe Interactions®* **2018**, *31*, 560–567, https://doi.org/10.1094/MPMI-11-17-0273-R.
347. Zhang, X.; Li, B.; Wang, Y.; Guo, Q.; Lu, X.; Li, S.; Ma, P. Lipopeptides, a Novel Protein, and Volatile Compounds Contribute to the Antifungal Activity of the Biocontrol Agent *Bacillus atrophaeus* CAB-1. *Appl Microbiol Biotechnol* **2013**, *97*, 9525–9534, https://doi.org/10.1007/s00253-013-5198-x.
348. Toure, Y.; Ongena, M.; Jacques, P.; Guiro, A.; Thonart, P. Role of Lipopeptides Produced by *Bacillus subtilis* GA1 in the Reduction of Grey Mould Disease Caused by Botrytis Cinerea on Apple. *J Appl Microbiol* **2004**, *96*, 1151–1160, https://doi.org/10.1111/j.1365-2672.2004.02252.x.
349. Kang, B.R.; Park, J.S.; Jung, W.-J. Antifungal Evaluation of Fengycin Isoforms Isolated from *Bacillus amyloliquefaciens* PPL against *Fusarium oxysporum* f. sp. *lycopersici*. *Microb Pathog* **2020**, *149*, 104509, https://doi.org/10.1016/j.micpath.2020.104509.
350. Lin, X.; Wang, J.; Hou, Z.; Ren, S.; Wang, W.; Yang, Y.; Yi, Y.; Zhang, Y.; Li, R. Antifungal Potential and Mechanism of *Bacillus velezensis* HeN-7 Isolated from Tobacco Leaves on *Bipolaris sorokiniana*. *Curr Microbiol* **2024**, *81*, 340, https://doi.org/10.1007/s00284-024-03858-8.
351. Valenzuela-Ruiz, V.; Robles-Montoya, R.I.; Parra-Cota, F.I.; Santoyo, G.; del Carmen Orozco-Mosqueda, Ma.; Rodríguez-Ramírez, R.; de los Santos-Villalobos, S. Draft Genome Sequence of *Bacillus paralicheniformis* TRQ65, a Biological Control Agent and Plant Growth-Promoting Bacterium Isolated from Wheat (*Triticum turgidum* subsp. *durum*) Rhizosphere in the Yaqui Valley, Mexico. *3 Biotech* **2019**, *9*, 436, https://doi.org/10.1007/s13205-019-1972-5.
352. Morales Sandoval, P.H.; Ortega Urquieta, M.E.; Valenzuela Ruíz, V.; Montañez Acosta, K.; Campos Castro, K.A.; Parra Cota, F.I.; Santoyo, G.; de los Santos Villalobos, S. Improving Beneficial Traits in *Bacillus cabrialesii* subsp. *cabrialesii* TE3^{T} through UV-Induced Genomic Changes. *Plants* **2024**, *13*, 2578, https://doi.org/10.3390/plants13182578.
353. Walaszczyk, A.; Jasińska, A.; Bernat, P.; Płaza, G.; Paraszkiewicz, K. Microplastics Influence on Herbicides Removal and Biosurfactants Production by a *Bacillus* sp. Strain Active against *Fusarium culmorum*. *Sci Rep* **2023**, *13*, 14618, https://doi.org/10.1038/s41598-023-41210-5.
354. Deng, Y.; Zhu, Y.; Wang, P.; Zhu, L.; Zheng, J.; Li, R.; Ruan, L.; Peng, D.; Sun, M. Complete Genome Sequence of *Bacillus subtilis* BSn5, an Endophytic Bacterium of *Amorphophallus konjac* with Antimicrobial Activity for the Plant Pathogen *Erwinia carotovora* subsp. *carotovora*. *J Bacteriol* **2011**, *193*, 2070–2071, https://doi.org/10.1128/JB.00129-11.
355. Pei, D.; Zhang, Q.; Zhu, X.; Yao, X.; Zhang, L. The Complete Genome Sequence Resource of Rhizospheric Soil-Derived *Bacillus velezensis* Yao, with Biocontrol Potential Against *Fusarium solani* -Induced Pepper Root Rot. *Phytopathology* **2023**, *113*, 580–583, https://doi.org/10.1094/PHYTO-03-22-0101-A.
356. Grady, E.N.; MacDonald, J.; Ho, M.T.; Weselowski, B.; McDowell, T.; Solomon, O.; Renaud, J.; Yuan, Z.-C. Characterization and Complete Genome Analysis of the Surfactin-Producing, Plant-Protecting Bacterium *Bacillus zelezensis* 9D-6. *BMC Microbiol* **2019**, *19*, 5, https://doi.org/10.1186/s12866-018-1380-8.
357. Mu, F.; Chen, X.; Fu, Z.; Wang, X.; Guo, J.; Zhao, X.; Zhang, B. Genome and Transcriptome Analysis to Elucidate the Biocontrol Mechanism of *Bacillus amyloliquefaciens* XJ5 against *Alternaria solani*. *Microorganisms* **2023**, *11*, 2055, https://doi.org/10.3390/microorganisms11082055.
358. Li, W.; Sun, L.; Wu, H.; Gu, W.; Lu, Y.; Liu, C.; Zhang, J.; Li, W.; Zhou, C.; Geng, H.; et al. *Bacillus velezensis* YXDHD1-7 Prevents Early Blight Disease by Promoting Growth and Enhancing Defense Enzyme Activities in Tomato Plants. *Microorganisms* **2024**, *12*, 921, https://doi.org/10.3390/microorganisms12050921.
359. Sharma, A.; Kaushik, N.; Sharma, A.; Bajaj, A.; Rasane, M.; Shouche, Y.S.; Marzouk, T.; Djébali, N. Screening of Tomato Seed Bacterial Endophytes for Antifungal Activity Reveals Lipopeptide Producing *Bacillus siamensis* Strain NKIT9 as a Potential Bio-Control Agent. *Front Microbiol* **2021**, *12*, https://doi.org/10.3389/fmicb.2021.609482.
360. Nikolić, I.; Berić, T.; Dimkić, I.; Popović, T.; Lozo, J.; Fira, D.; Stanković, S. Biological Control of *Pseudomonas syringae* pv. *aptata* on Sugar Beet with *Bacillus pumilus* SS-10.7 and *Bacillus amyloliquefaciens* (SS-12.6 and SS-38.4) Strains. *J Appl Microbiol* **2019**, *126*, 165–176, https://doi.org/10.1111/jam.14070.
361. Tang, Y.; Lei, J.; Ma, X.; Li, J.; Li, H.; Liu, Z. Identification and Characterization of a Novel Bacteriocin Gene Cluster in *Lysinibacillus boronitolerans*. *Biotechnol Appl Biochem* **2023**, *70*, 1860–1869, https://doi.org/10.1002/bab.2488.
362. Xie, S.; Jiang, H.; Ding, T.; Xu, Q.; Chai, W.; Cheng, B. *Bacillus amyloliquefaciens* FZB42 Represses Plant miR846 to Induce Systemic Resistance via a Jasmonic Acid-dependent Signalling Pathway. *Mol Plant Pathol* **2018**, *19*, 1612–1623, https://doi.org/10.1111/mpp.12634.
363. Dong, H.; Gao, R.; Dong, Y.; Yao, Q.; Zhu, H. *Bacillus velezensis* RC116 Inhibits the Pathogens of Bacterial Wilt and *Fusarium* Wilt in Tomato with Multiple Biocontrol Traits. *Int J Mol Sci* **2023**, *24*, 8527, https://doi.org/10.3390/ijms24108527.
364. Mosela, M.; Andrade, G.; Massucato, L.R.; de Araújo Almeida, S.R.; Nogueira, A.F.; de Lima Filho, R.B.; Zeffa, D.M.; Mian, S.; Higashi, A.Y.; Shimizu, G.D.; et al. *Bacillus velezensis* Strain Ag75 as a New Multifunctional Agent for Biocontrol, Phosphate Solubilization and Growth Promotion in Maize and Soybean Crops. *Sci Rep* **2022**, *12*, 15284, https://doi.org/10.1038/s41598-022-19515-8.
365. Hudson, L.K.; Orellana, L.A.G.; Bryan, D.W.; Moore, A.; Munafo, J.P.; den Bakker, H.C.; Denes, T.G. Phylogeny of the *Bacillus altitudinis* Complex and Characterization of a Newly Isolated Strain with Antilisterial Activity. *J Food Prot* **2021**, *84*, 1321–1332, https://doi.org/10.4315/JFP-20-498.
366. Qiao, J.; Zhang, R.; Liu, Y.; Liu, Y. Evaluation of the Biocontrol Efficiency of *Bacillus subtilis* Wettable Powder on Pepper Root Rot Caused by *Fusarium solani*. *Pathogens* **2023**, *12*, 225, https://doi.org/10.3390/pathogens12020225.
367. Ashajyothi, M.; Mahadevakumar, S.; Venkatesh, Y.N.; Sarma, P.V.S.R.N.; Danteswari, C.; Balamurugan, A.; Prakash, G.; Khandelwal, V.; Tarasatyavathi, C.; Podile, A.R.; et al. Comprehensive Genomic Analysis of *Bacillus subtilis* and *Bacillus*

paralicheniformis Associated with the Pearl Millet Panicle Reveals Their Antimicrobial Potential against Important Plant Pathogens. *BMC Plant Biol* **2024**, *24*, 197, https://doi.org/10.1186/s12870-024-04881-4.

368. Li, B.; Yang, P.; Feng, Y.; Du, C.; Qi, G.; Zhao, X. Rhizospheric Microbiota of Suppressive Soil Protect Plants against *Fusarium solani* Infection. *Pest Manag Sci* **2024**, *80*, 4186–4198, https://doi.org/10.1002/ps.8122.

369. Cheffi, M.; Chenari Bouket, A.; Alenezi, F.N.; Luptakova, L.; Belka, M.; Vallat, A.; Rateb, M.E.; Tounsi, S.; Triki, M.A.; Belbahri, L. *Olea europaea* L. Root Endophyte *Bacillus velezensis* OEE1 Counteracts Oomycete and Fungal Harmful Pathogens and Harbours a Large Repertoire of Secreted and Volatile Metabolites and Beneficial Functional Genes. *Microorganisms* **2019**, *7*, 314, https://doi.org/10.3390/microorganisms7090314.

370. Mnif, I.; Hammami, I.; Triki, M.A.; Azabou, M.C.; Ellouze-Chaabouni, S.; Ghribi, D. Antifungal Efficiency of a Lipopeptide Biosurfactant Derived from *Bacillus subtilis* SPB1 versus the Phytopathogenic Fungus, *Fusarium solani*. *Environmental Science and Pollution Research* **2015**, *22*, 18137–18147, https://doi.org/10.1007/s11356-015-5005-6.

371. Duan, Y.; Zhao, L.; Jiang, W.; Chen, R.; Zhang, R.; Chen, X.; Yin, C.; Mao, Z. The Phlorizin-Degrading *Bacillus licheniformis* XNRB-3 Mediates Soil Microorganisms to Alleviate Apple Replant Disease. *Front Microbiol* **2022**, *13*, https://doi.org/10.3389/fmicb.2022.839484.

372. Chen, X.; Wang, Y.; Gao, Y.; Gao, T.; Zhang, D. Inhibitory Abilities of *Bacillus* Isolates and Their Culture Filtrates against the Gray Mold Caused by *Botrytis cinerea* on Postharvest Fruit. *Plant Pathol J* **2019**, *35*, 425–436, https://doi.org/10.5423/PPJ.OA.03.2019.0064.

373. Stoll, A.; Salvatierra-Martínez, R.; González, M.; Araya, M. The Role of Surfactin Production by *Bacillus velezensis* on Colonization, Biofilm Formation on Tomato Root and Leaf Surfaces and Subsequent Protection (ISR) against *Botrytis cinerea*. *Microorganisms* **2021**, *9*, 2251, https://doi.org/10.3390/microorganisms9112251.

374. Samaras, A.; Karaoglanidis, G.S.; Tzelepis, G. Insights into the Multitrophic Interactions between the Biocontrol Agent *Bacillus Subtilis* MBI 600, the Pathogen *Botrytis Cinerea* and Their Plant Host. *Microbiol Res* **2021**, *248*, 126752, https://doi.org/10.1016/j.micres.2021.126752.

375. Baptista, J.P.; Teixeira, G.M.; de Jesus, M.L.A.; Bertê, R.; Higashi, A.; Mosela, M.; da Silva, D.V.; de Oliveira, J.P.; Sanches, D.S.; Brancher, J.D.; et al. Antifungal Activity and Genomic Characterization of the Biocontrol Agent *Bacillus velezensis* CMRP 4489. *Sci Rep* **2022**, *12*, 17401, https://doi.org/10.1038/s41598-022-22380-0.

376. Balderas-Ruíz, K.A.; Gómez-Guerrero, C.I.; Trujillo-Roldán, M.A.; Valdez-Cruz, N.A.; Aranda-Ocampo, S.; Juárez, A.M.; Leyva, E.; Galindo, E.; Serrano-Carreón, L. *Bacillus velezensis* 83 Increases Productivity and Quality of Tomato (*Solanum lycopersicum* L.): Pre and Postharvest Assessment. *Curr Res Microb Sci* **2021**, *2*, 100076, https://doi.org/10.1016/j.crmicr.2021.100076.

377. Li, B.; Wan, J.; Sha, J.; Tian, M.; Wang, M.; Zhang, X.; Sun, W.; Mao, Y.; Min, J.; Qin, Y.; et al. Genomics Assisted Functional Characterization of *Bacillus velezensis* E as a Biocontrol and Growth Promoting Bacterium for Lily. *Front Microbiol* **2022**, *13*, https://doi.org/10.3389/fmicb.2022.976918.

378. Altimira, F.; Godoy, S.; Arias-Aravena, M.; Araya, B.; Montes, C.; Castro, J.F.; Dardón, E.; Montenegro, E.; Pineda, W.; Viteri, I.; et al. Genomic and Experimental Analysis of the Biostimulant and Antagonistic Properties of Phytopathogens of *Bacillus safensis* and *Bacillus siamensis*. *Microorganisms* **2022**, *10*, 670, https://doi.org/10.3390/microorganisms10040670.

379. Thomloudi, E.-E.; Tsalgatidou, P.C.; Baira, E.; Papadimitriou, K.; Venieraki, A.; Katinakis, P. Genomic and Metabolomic Insights into Secondary Metabolites of the Novel *Bacillus halotolerans* Hil4, an Endophyte with Promising Antagonistic Activity against Gray Mold and Plant Growth Promoting Potential. *Microorganisms* **2021**, *9*, 2508, https://doi.org/10.3390/microorganisms9122508.

380. Ahmad, Z.; Wu, J.; Chen, L.; Dong, W. Isolated *Bacillus subtilis* Strain 330-2 and Its Antagonistic Genes Identified by the Removing PCR. *Sci Rep* **2017**, *7*, 1777, https://doi.org/10.1038/s41598-017-01940-9.

381. Ji, S.; Tian, Y.; Li, J.; Xu, G.; Zhang, Y.; Chen, S.; Chen, Y.; Tang, X. Complete Genome Sequence of *Bacillus cereus* Z4, a Biocontrol Agent against Tobacco Black Shank, Isolated from the Western Pacific Ocen. *Mar Genomics* **2023**, *72*, 101071, https://doi.org/10.1016/j.margen.2023.101071.

382. Eltokhy, M.A.; Saad, B.T.; Eltayeb, W.N.; Yahia, I.S.; Aboshanab, K.M.; Ashour, M.S.E. Exploring the Nature of the Antimicrobial Metabolites Produced by *Paenibacillus ehimensis* Soil Isolate MZ921932 Using a Metagenomic Nanopore Sequencing Coupled with LC-Mass Analysis. *Antibiotics* **2021**, *11*, 12, https://doi.org/10.3390/antibiotics11010012.

383. Dang, T.; Loll, B.; Müller, S.; Skobalj, R.; Ebeling, J.; Bulatov, T.; Gensel, S.; Göbel, J.; Wahl, M.C.; Genersch, E.; et al. Molecular Basis of Antibiotic Self-Resistance in a Bee Larvae Pathogen. *Nat Commun* **2022**, *13*, 2349, https://doi.org/10.1038/s41467-022-29829-w.

384. Priyanto, J.A.; Prastya, M.E.; Astuti, R.I.; Kristiana, R. The Antibacterial and Antibiofilm Activities of the Endophytic Bacteria Associated with *Archidendron pauciflorum* against Multidrug-Resistant Strains. *Appl Biochem Biotechnol* **2023**, *195*, 6653–6674, https://doi.org/10.1007/s12010-023-04382-4.

385. Kukreti, A.; Kotasthane, A.S.; Tandon, A.L.; Nekkanti, A.; Prasannakumar, M.K.; Devanna, P.; Aravindaram, K.; Sreedevi, K.; Sushil, S.N.; Manjunatha, C. Hybrid de Novo Whole Genome Assembly of Lipopeptide Producing Novel *Bacillus thuringiensis* Strain NBAIR BtAr Exhibiting Antagonistic Activity against *Sclerotium rolfsii*. *Microb Pathog* **2024**, *195*, 106867, https://doi.org/10.1016/j.micpath.2024.106867.

386. Mokhtar, N.F.K.; Hashim, A.M.; Hanish, I.; Zulkarnain, A.; Raja Nhari, R.M.H.; Abdul Sani, A.A.; Abbasiliasi, S.; Ariff, A.; Mustafa, S.; Rahim, R.A. The Discovery of New Antilisterial Proteins From *Paenibacillus polymyxa* Kp10 via Genome Mining and Mass Spectrometry. *Front Microbiol* **2020**, *11*, https://doi.org/10.3389/fmicb.2020.00960.
